# Whole‐Cell P450 Biocatalysis Using Engineered *Escherichia coli* with Fine‐Tuned Heme Biosynthesis

**DOI:** 10.1002/advs.202205580

**Published:** 2022-12-16

**Authors:** Baodong Hu, Haibo Yu, Jingwen Zhou, Jianghua Li, Jian Chen, Guocheng Du, Sang Yup Lee, Xinrui Zhao

**Affiliations:** ^1^ Key Laboratory of Industrial Biotechnology Ministry of Education School of Biotechnology Jiangnan University 1800 Lihu Road Wuxi Jiangsu 214122 China; ^2^ Science Center for Future Foods Jiangnan University 1800 Lihu Road Wuxi Jiangsu 214122 China; ^3^ Jiangsu Province Engineering Research Center of Food Synthetic Biotechnology Jiangnan University 1800 Lihu Road Wuxi Jiangsu 214122 China; ^4^ Engineering Research Center of Ministry of Education on Food Synthetic Biotechnology Jiangnan University 1800 Lihu Road Wuxi Jiangsu 214122 China; ^5^ Key Laboratory of Carbohydrate Chemistry and Biotechnology Ministry of Education Jiangnan University 1800 Lihu Road Wuxi Jiangsu 214122 China; ^6^ Metabolic and Biomolecular Engineering National Research Laboratory Department of Chemical and Biomolecular Engineering (BK21 Plus Program) BioProcess Engineering Research Center Bioinformatics Research Center, and Institute for the BioCentury Korea Advanced Institute of Science and Technology (KAIST) Daejeon Yuseong‐gu 34141 Republic of Korea

**Keywords:** chemical intermediates, cytochrome P450 enzymes, drugs, *Escherichia coli*, fine‐tuned heme biosynthesis system, natural products, whole‐cell biocatalysts

## Abstract

By exploiting versatile P450 enzymes, whole‐cell biocatalysis can be performed to synthesize valuable compounds in *Escherichia coli*. However, the insufficient supply of heme limits the whole‐cell P450 biocatalytic activity. Here a strategy for improving intracellular heme biosynthesis to enhance the catalytic efficiencies of P450s is reported. After comparing the effects of improving heme transport and biosynthesis on P450 activities, intracellular heme biosynthesis is optimized through the integrated expression of necessary synthetic genes at proper ratios and the assembly of rate‐limiting enzymes using DNA‐guided scaffolds. The intracellular heme level is fine‐tuned by the combined use of mutated heme‐sensitive biosensors and small regulatory RNA systems. The catalytic efficiencies of three different P450s, BM3, sca‐2, and CYP105D7, are enhanced through fine‐tuning heme biosynthesis for the synthesis of hydroquinone, pravastatin, and 7,3′,4′‐trihydroxyisoflavone as example products of chemical intermediate, drug, and natural product, respectively. This strategy of fine‐tuned heme biosynthesis will be generally useful for developing whole‐cell biocatalysts involving hemoproteins.

## Graphical abstract

Here, the development of an engineered *E. coli* strain capable of improved heme supply suitable for whole‐cell P450 biocatalysts is reported. The intracellular heme level is fine‐tuned by the combined use of mutated heme‐sensitive biosensors and small regulatory RNA systems. This strategy of fine‐tuned heme biosynthesis will be generally useful for developing whole‐cell biocatalysts involving hemoproteins.

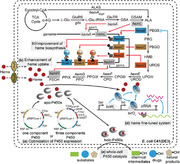

## 1 Introduction

Cytochrome P450 enzymes (P450s, CYP) belong to a superfamily of hemoproteins^[^ ^1^ ^]^ that catalyze various reactions, including hydroxylation, dimerization, and epoxidation.^[^ ^2^, ^3^ ^]^ Importantly, these reactions are carried out using diverse compounds such as terpenoids, fatty acids, alkaloids, and steroids as substrates.^[^ ^4^ ^]^ Due to their unique site‐, regio‐, and stereo‐selectivities, P450s have been explored as reliable and environmentally‐friendly biocatalysts to synthesize valuable natural products and drugs.^[^ ^5^, ^6^ ^]^ *Escherichia coli* has been frequently applied to express P450 genes to provide high yields of target proteins to satisfy the increasing demands for large‐scale biosynthesis of valuable chemicals. However, it is not convenient to utilize purified P450s for bioconversion reactions because of the complex purification processes and the expensive requirement for NAD(P)H cofactors.^[^ ^7^ ^]^ Hence, engineered *E. coli* strains heterologously expressing P450 genes have been developed as promising whole‐cell biocatalysts that synthesize target compounds by providing crucial precursors, cofactors, and suitable environments for multistep reactions.^[^ ^8^ ^]^

**Figure 1:**
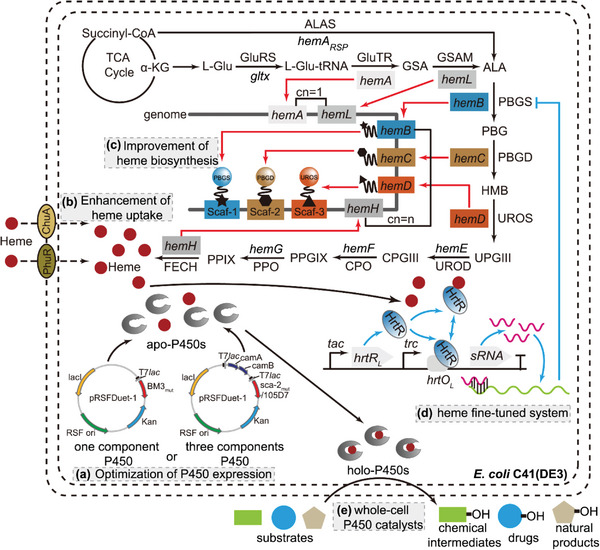
Engineering strategies for the efficient whole *E. coli* cell P450 biocatalysts through fine‐tuning heme supply. a) Optimization of the expression of P450 genes using the high‐copy number plasmid pRSFDuet‐1. b) Enhancement of heme uptake by introducing heterologous heme importers. ChuA is a heme receptor in the outer membrane of *E. coli* Nissle 1917, while PhuR is a heme receptor in the outer membrane of *Pseudomonas aeruginosa*. c) Improvement of heme biosynthesis. The red arrows indicate the amplified genes in the heme synthetic pathway. The pentacle, rhomb, and triangle indicate the zinc‐finger proteins ADB1, ADB2, and ADB3, respectively. d) Dynamic regulation of heme biosynthesis by the fine‐tuned system. The blue arrows indicate the regulation of heme biosynthesis by intracellular heme level. HrtR is the heme regulator from *Lactococcus lactis*, while *hrtO_L_* is the binding site of HrtR. e) Representative biocatalysis using the efficient whole‐cell P450 catalysts. Abbreviations are: *α*‐KG, *α*‐ketoglutarate; l‐Glu, l‐glutamate; GluRS, glutamyl‐tRNA synthetase; l‐Glu‐tRNA, l‐glutamate‐tRNA; GluTR, glutamyl‐tRNA reductase; GSA, glutamate‐1‐semialdehyde; GSAM, glutamate‐1‐semialdehyde 2,1‐aminomutase; ALA, 5‐aminolevulinic acid; ALAS, 5‐aminolevulinate synthase; PBG, porphobilinogen; PBGS, porphobilinogen synthase; HMB, 1‐hydroxymethylbilane; PBGD, porphobilinogen deaminase; UPGIII, uroporphyrinogen‐III; UROS, uroporphyrinogen‐III synthase; CPGIII, coproporphyrinogen‐III; UROD, uroporphyrinogen‐III decarboxylase; PPGIX, protoporphyrinogen‐IX; CPO, coproporphyrinogen‐III oxidase; PPIX, protoporphyrin‐IX; PPO, protoporphyrinogen oxidase; FECH, ferrochelatase; cn, copy number; Scaf, DNA‐guided scaffold.

Although P450 genes can be overexpressed in *E. coli*, the insufficient supply of heme is a bottleneck in preparing highly active whole‐cell biocatalysts. In *E. coli*, 5‐aminolevulinic acid (ALA), the precursor of heme, can be synthesized through the heterologous C4 pathway involving ALA synthetase (ALAS) or the endogenous C5 pathway involving glutamyl‐tRNA synthetase (GluRS), glutamyl‐tRNA reductase (GluTR), and glutamate‐1‐semialdehyde 2,1‐aminomutase (GSAM). ALA is subsequently converted to heme through a series of reactions catalyzed by porphobilinogen synthase (PBGS), porphobilinogen deaminase (PBGD), uroporphyrinogen III synthase (UROS), uroporphyrinogen III decarboxylase (UROD), coproporphyrinogen III oxidase (CPO), protoporphyrinogen oxidase (PPO), and ferrochelatase (FECH) (**Figure** **1**).^[^ ^9^ ^]^ Due to the multiple limiting reaction steps catalyzed by GluRS, ALAS, PBGS, PBGD, UROS, and FECH in the heme biosynthetic pathway,^[^ ^10^ ^]^ it is challenging to provide sufficient heme for the production of highly active P450s in *E. coli*. Furthermore, common *E. coli* strains do not possess a heme importer, making it difficult to supplement heme extracellularly.^[^ ^11^ ^]^

Exogenous supplementation of ALA or heme was attempted to enhance the intracellular supply of heme in *E. coli*. Although the activities of P450s were improved by adding ALA (0.5–1.0 mm),^[^ ^12^ ^]^ this approach increased the cost of cultivation by over 60%, presenting challenges in cost‐effective industrial production.^[^ ^13^ ^]^ Instead of ALA, heme supplementation can be attempted after heterologously expressing the genes encoding heme importer from pathogenic strains such as *Plesiomonas shigelloides* and *E. coli* O157:H7^[^ ^14^, ^15^ ^]^ in common *E. coli* hosts or using apathogenic *E. coli* Nissle 1917 strain as a host.^[^ ^16^ ^]^ However, heme supplementation has never been utilized to highly express active P450 enzymes.

For these reasons, enhanced in vivo biosynthesis of appropriate heme levels is thought to be the best strategy for producing highly active P450s. However, previous studies mainly focused on producing heme as the final product^[^ ^10^, ^17^ ^]^ or individually overexpressing the GluRS, ALAS, or FECH gene to improve the catalytic efficiencies of P450s.^[^ ^18^, ^19^, ^20^ ^]^ Consequently, the suitable expression strategies for these genes and the synergistic effect of multiple rate‐limiting enzymes (PBGS, PBGD, and UROS) have been neglected.

As free heme is toxic to cells^[^ ^21^ ^]^ and the excessive expression of genes for heme synthesis will consume undesirably much cellular resources, the balance between the expression of P450 genes and the genes involved in heme synthesis is essential to develop high‐performance whole‐cell biocatalysts. The heme‐responsive biosensor (HrtR regulator and its binding site *hrtO* from *Lactococcus lactis*) has been used to design an *E. coli* whole‐cell biosensor for detecting heme in urine.^[^ ^22^ ^]^ Additionally, heme‐responsive biosensors and CRISPRi have been used to regulate the transcription of several heme biosynthesis genes (*hemB*, *hemC*, and *hemH*) in *E. coli*.^[^ ^23^ ^]^ However, this system should be modified to provide sufficient heme in the cells overexpressing P450 genes. The sensitivity to leaky suppression within the CRISPRi logic gate and the retroactivity effect due to the shared pool of dCas proteins limit the performance of CRISPRi.^[^ ^24^ ^]^ Unfortunately, even with the fusion of an AAV degradation tag to dCas9,^[^ ^25^ ^]^ CRISPRi is unsuitable for the dynamic regulation of the heme biosynthetic pathway because the shortest regulatory time is over 6 h.^[^ ^23^ ^]^ Furthermore, using the dCas9 gene will induce abnormal morphologies and off‐target effects in *E. coli*.^[^ ^26^, ^27^ ^]^ Thus, developing a new regulatory system is necessary to fine‐tune the heme supply and consequently develop whole‐cell P450 biocatalysts.

Here we report the development of an engineered *E. coli* strain capable of improved heme supply suitable for whole‐cell P450 biocatalysts (Figure 1). By comparing the effects of enhancing heme transport and biosynthesis on the catalytic efficiencies of P450s, the necessary heme biosynthetic genes were expressed at proper ratios and several rate‐limiting enzymes were assembled by DNA‐guided scaffolds. A fine‐tuned intracellular heme biosynthetic system was constructed using the mutated heme‐sensitive biosensor and small regulatory RNA systems. Then, the self‐sufficient P450 enzyme (BM3_mut_) and three‐component P450s (sca‐2_mut_ and CYP105D7) were employed as representative P450s in the whole‐cell biocatalysis to produce hydroquinone, pravastatin, and 7,3′,4′‐trihydroxyisoflavone as examples of chemical intermediates, drugs, and natural products, respectively.

## 2 Results

### 2.1 Selecting a Suitable Expression System for Whole‐Cell P450s Biocatalysts

**Figure 2:**
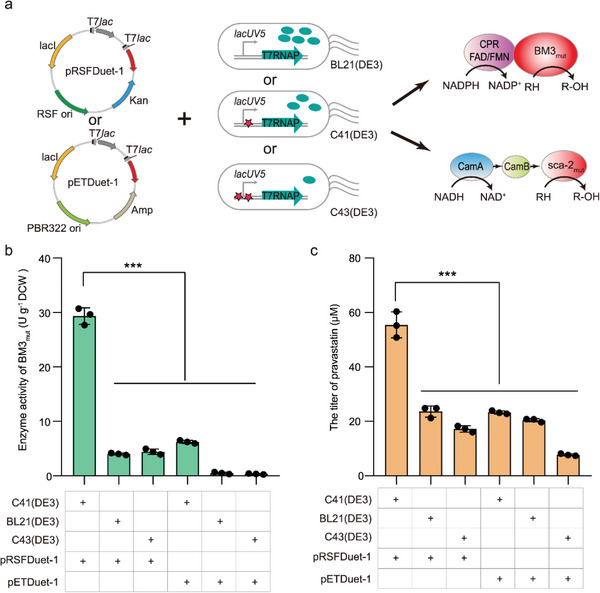
Selecting a suitable expression system for developing whole‐cell P450 biocatalysts. a) Different strains and plasmids were examined to develop whole‐cell self‐sufficient P450 BM3_mut_ and three‐component P450sca‐2_mut_ biocatalysts. b) The activity of BM3_mut_ biocatalyst using 7‐ethoxycoumarin as a substrate. Catalytic conditions for BM3_mut_: 10 OD_600_ mL^−1^ of cells in potassium phosphate buffer (pH 8.0, 100 mm, 0.5 mL), 0.8 mm of 7‐ethoxycoumarin, 28 °C, 220 rpm, 1 h. c) The titer of pravastatin produced by sca‐2_mut_ biocatalyst. Catalytic conditions for sca‐2_mut_: 30 OD_600_ mL^−1^ of cells in potassium phosphate buffer (pH 8.0, 100 mm, 10% v/v glycerol, 2 mL), 0.73 mm of mevastatin, 30 °C, 220 rpm, 12 h. Data presented as mean values ± SD from three independent biological replicates (*n* = 3). Black circles represent individual data points. Two‐tailed‐Student's t‐test evaluated significance. ***p < 0.001.

To obtain an optimal expression system for P450 genes, three host strains, *E. coli* BL21(DE3), C41(DE3), and C43(DE3), and plasmids of different copy numbers, a high‐copy number plasmid pRSFDuet‐1 and a medium‐copy number plasmid pETDuet‐1, were examined. The self‐sufficient P450 BM3_mut_ and the three‐component P450 sca‐2_mut_ and its matching ferredoxin reductase CamA and ferredoxin CamB were chosen as representative P450s (**Figures** 1a and **2**a). Although higher expression levels of P450s were obtained using the high‐copy number plasmid pRSFDuet‐1 (Figure S1, Supporting Information), the whole‐cell catalytic activities were significantly different among the three utilized *E. coli* strains. Due to the moderate expression level of T7 RNA polymerase,^[^ ^28^ ^]^ the C41(DE3) strain was more appropriate for the folding of P450s compared with the BL21(DE3) and C43(DE3) strains. The highest activities of BM3_mut_ (29.4 ± 1.5 U g^−1^ DCW) and sca‐2_mut_ (55.5 ± 4.8 µm) were obtained in the C41‐pRSF‐BM3_mut_ and C41‐pRSF‐sca‐2_mut_ strains, respectively (Figure 2b,c). Thus, the C41(DE3) strain and pRSFDuet‐1 plasmid were selected to develop efficient whole‐cell P450 biocatalysts.

### 2.2 Improving Heme Supply by Introducing Heterologous Heme Importers

**Figure 3:**
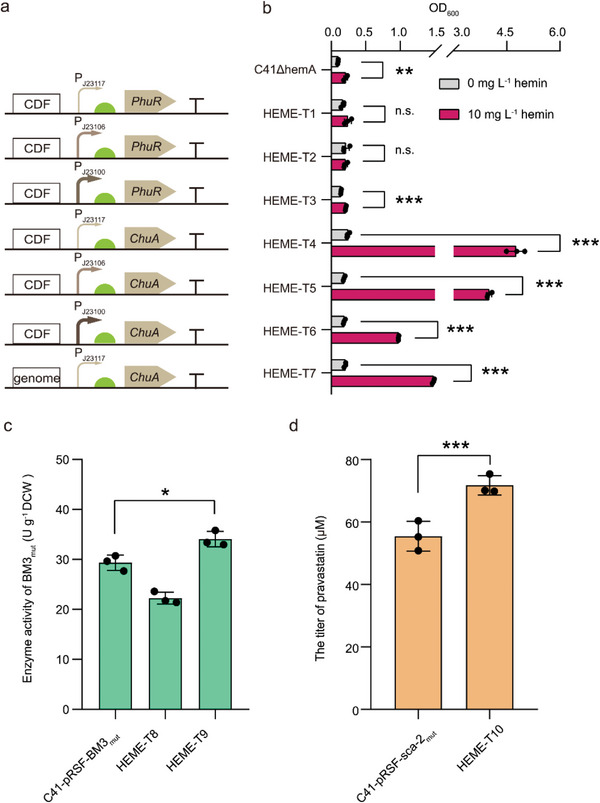
Improving heme supply by introducing heterologous heme importers. a) Expression of the *phuR* gene from *P. aeruginosa* and the *chuA* gene from *E. coli* Nissle 1917 under the control of constitutive promoters in HEME‐T1 to HEME‐T7 strains. P_J23100_, P_J23116_, and P_J23117_ are strong, moderate, and weak promoters, respectively. b) Growth of HEME‐T1 to HEME‐T7 strains after introducing heterologous heme importers. c) The effect of ChuA under the control of constitutive promoter P_J23117_ (HEME‐T8 strain) and inducible T7 promoter (HEME‐T9 strain) on the whole‐cell activity of P450 BM3_mut_. Catalytic conditions for BM3_mut_: 10 OD_600_ mL^−1^ of cells in potassium phosphate buffer (pH 8.0, 100 mm, 0.5 mL), 0.8 mm of 7‐ethoxycoumarin, 28 °C, 220 rpm, 1 h. d) The effect of ChuA under the control of inducible T7 promoter (HEME‐T10 strain) on the whole‐cell activity of P450 sca‐2_mut_. Catalytic conditions for sca‐2_mut_: 30 OD_600_ mL^−1^ of cells in potassium phosphate buffer (pH 8.0, 100 mm, 10% v/v glycerol, 2 mL), 0.73 mm of mevastatin, 30 °C, 220 rpm, 12 h. Data presented as mean values ± SD from three independent biological replicates (*n* = 3). Black circles represent individual data points. Two‐tailed‐Student's t‐test evaluated significance and *p* > 0.05 presents no significance (n.s.). *p < 0.05, **p < 0.01, ***p < 0.001.

After 1.0 ± 0.0 µm of purified BM3_mut_ and 0.8 ± 0.0 µm of purified sca‐2_mut_ were produced using the optimized expression system, more intracellular heme was required to prepare efficient whole‐cell biocatalysts. Hence, the enhancement of heme uptake in the C41(DE3) strain was investigated. The efficiencies of heme uptake were evaluated by the growth improvement of the C41ΔhemA strain, in which the *hemA* gene encoding the essential enzyme (GluTR) responsible for the synthesis of ALA and heme was deleted. The deletion of the *hemA* gene resulted in a lethal phenotype which could be rescued by supplementing ALA, but not by adding exogenous heme.^[^ ^29^ ^]^ To screen an efficient heme transporter for *E. coli* C41(DE3), the genes encoding heme receptor in the outer membrane from *P. aeruginosa* (*phuR*)^[^ ^30^ ^]^ and *E. coli* Nissle 1917 (*chuA*)^[^ ^11^ ^]^ were constitutively expressed in the C41ΔhemA strain using the medium‐copy‐number plasmid pCDFDuet‐1 with P_J23100_ (strong), P_J23106_ (moderate), or P_J23117_ (weak) promoter, respectively (HEME‐T1 to HEME‐T6 strains) (**Figures** 1b and **3**a). Compared with the C41ΔhemA strain expressing *phuR* gene (HEME‐T1 to HEME‐T3 strains), the growth of the C41ΔhemA strain (HEME‐T4 to HEME‐T6 strains) was significantly improved when the *chuA* gene was expressed together with 10 mg L^−1^ hemin supplementation (Figure 3b). Since the function of PhuR and ChuA are dependent on the formation of a complex with TonB,^[^ ^31^ ^]^ the weak heme import activity of PhuR is mainly due to the significant difference between TonB in C41ΔhemA and *P. aeruginosa* (sequence similarity: 31.8%). Hence, the ChuA from *E. coli* Nissle 1917 was selected to construct the heme import system.

In addition, the weak promoter (P_J23117_) was found to be more suitable than the stronger promoter (P_J23100_) for the expression of the *chuA* gene (Figure 3b). Next, the integrated expression of the *chuA* gene (HEME‐T7 strain) was investigated to reduce the metabolic burden. However, compared with the HEME‐T4 strain (C41ΔhemA strain harboring pCDF‐P_J23117_‐ChuA), the capacity for heme uptake was reduced in the HEME‐T7 strain (Figure 3b). The genetically encoded ratiometric fluorescent sensors^[^ ^32^, ^33^ ^]^ detected the capacity of heme uptake, and 2.5 nm of hemin was imported into the cells through ChuA in the HEME‐T4 strain when 10 mg L^−1^ hemin was supplemented (Figure S2, Supporting Information).

Thus, the *chuA* gene expressed under the control of the weak promoter (P_J23117_) using plasmid pCDFDuet‐1 was selected to determine the effect of ChuA on the whole‐cell activity of BM3_mut_. The plasmids pCDF‐P_J23117_‐ChuA and pRSF‐BM3_mut_ were co‐expressed in the C41(DE3) strain to generate the HEME‐T8 strain. The whole‐cell activity of the HEME‐T8 strain decreased to 22.2 ± 1.2 U g^−1^ DCW when 10 mg L^−1^ hemin was supplemented (Figure 3c), suggesting that the weak constitutive expression of the *chuA* gene still negatively impacted the whole‐cell activity of BM3_mut_. Thus, an inducible T7 promoter was selected to express the *chuA* gene (HEME‐T9 strain), increasing the whole‐cell activity by 16.1% compared with the control strain C41‐pRSF‐BM3_mut_ (Figure 3c). The *chuA* gene under the control of the T7 promoter was used to improve the catalytic efficiency of HEME‐T10 strain (harboring pRSF‐sca‐2_mut_‐CAB and pCDF‐T7‐ChuA), increasing the whole‐cell activity by 29.4% compared with the control strain C41‐pRSF‐sca‐2_mut_ (Figure 3d). Taken together, the increased intracellular heme supply by enhancing heme uptake (transporting 2.5 nm hemin) was beneficial for enhancing the activities of whole‐cell P450 biocatalysts to some extent, but not satisfactorily high enough.

### 2.3 Improving Heme Supply by Enhancing the Endogenous Biosynthetic Pathway

**Figure 4:**
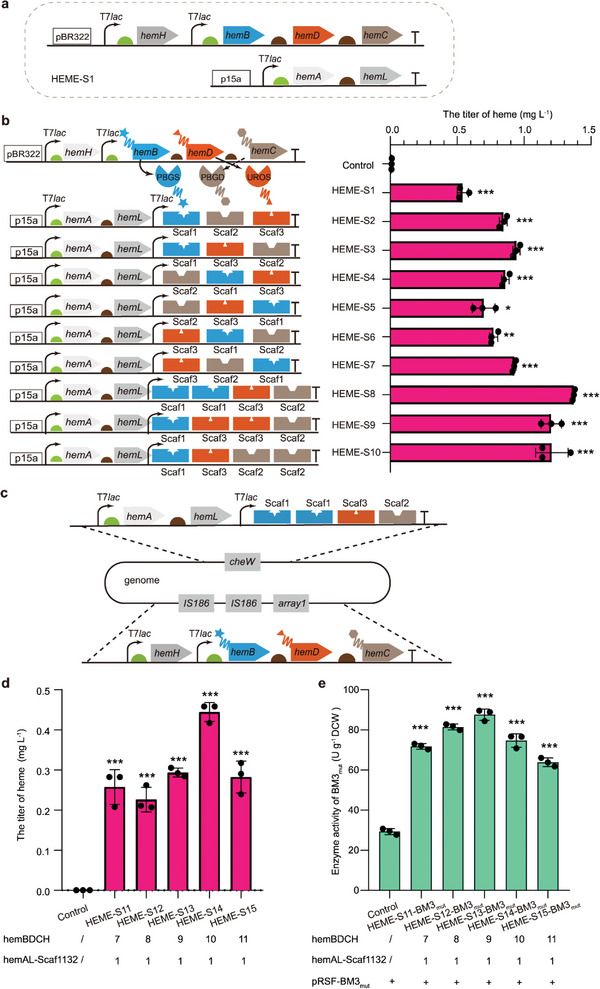
Improving heme supply by enhancing endogenous biosynthetic pathway. a) Overexpression of *hemA*, *hemL*, *hemB*, *hemC*, *hemD*, and *hemH* genes enhances heme biosynthesis. Light green semicircle and brown semicircle indicate different ribosome binding sites. b) The effect of PBGS, PBGD, and UROS assembly by DNA‐guided scaffolds on the synthesis of intracellular heme in the HEME‐S2 to HEME‐S10 strains. The HEME‐S2 to HEME‐S7 strains contain the binding sites in different orders and the HEME‐S8 to HEME‐S10 strains contain different ratios of binding sites of scaffolds. The C41(DE3) strain (the concentration of heme: 0.01 mg L^−1^) was used as a control strain. c) Chromosome integrated expression of *hemA*, *hemL*, *hemB*, *hemC*, *hemD*, and *hemH* genes at proper ratios by CRISPR‐associated transposases. d) Synthesis of intracellular heme in the HEME‐S11 to HEME‐S15 strains. The HEME‐S11 to HEME‐S15 strains have one copy of pT7‐*hemA*‐*hemL*‐pT7‐*scaffold1‐scaffold1‐scaffold3‐scaffold2* integrated in the chromosome, and different copies of pT7‐*hemH*‐pT7‐*ADB1‐hemB‐ADB3‐hemD‐ADB2‐hemC* (HEME‐S11, 7 copies; HEME‐S12, 8 copies; HEME‐S13, 9 copies; HEME‐S14, 10 copies; HEME‐S15, 11 copies). Again, the C41(DE3) strain was used as a control strain. e) The effect of recombinant *E. coli* strains with the integrated expression of necessary heme biosynthesis genes on the whole‐cell activity of BM3_mut_. The C41‐pRSF‐BM3_mut_ strain was used as a control strain. Catalytic conditions for BM3_mut_: 10 OD_600_ mL^−1^ of cells in potassium phosphate buffer (pH 8.0, 100 mm, 0.5 mL), 0.8 mm of 7‐ethoxycoumarin, 28 °C, 220 rpm, 1 h. Data presented as mean values ± SD from three independent biological replicates (*n* = 3). Black circles represent individual data points. Two‐tailed‐Student's t‐test evaluated significance. *p < 0.05, **p < 0.01, ***p < 0.001. Scaf, DNA‐guided scaffold.

Due to the weak effect of enhancing heme uptake on intracellular heme supply (Figure 3c), the enhancement of endogenous heme biosynthetic pathway was pursued instead (Figure 1c). Based on the previous studies on the main rate‐limiting enzymes in the heme biosynthetic pathway in *E. coli*, the expression of *hemB*, *hemC*, *hemD*, and *hemH* should be enhanced.^[^ ^10^ ^]^ As the high‐copy‐number plasmid pRSFDuet‐1 was used to express P450 genes, these four genes (*hemB*, *hemC*, *hemD*, and *hemH*) were overexpressed using the medium‐copy‐number plasmid pETDuet‐1 (**Figure** **4**a). Additionally, the synthesis of ALA (GluTR encoded by *hemA* and GSAM encoded by *hemL*) was also slightly increased by overexpressing *hemA* and *hemL* using the low‐copy‐number plasmid pACYCDuet‐1 (Figure 4a). Compared with the negligible heme concentration (0.01 ± 0.00 mg L^−1^) in the C41(DE3) strain, 0.5 ± 0.0 mg L^−1^ of heme was synthesized in the HEME‐S1 strain harboring pET‐hemBDCH and pACYC‐hemAL. However, this heme concentration was relatively low compared with those reported previously (6.6 mg L^−1^) (Figure 4b).^[^ ^10^ ^]^

As PBGS is feedback inhibited by the intermediate of heme (protoporphyrinogen IX)^[^ ^34^ ^]^ and the product of PBGD (hydroxymethylbilane, HMB) can be spontaneously transformed to a byproduct uroporphyrinogen I,^[^ ^35^ ^]^ DNA‐guided scaffolds were employed to assemble three key cascade enzymes (PBGS, PBGD, and UROS) to further enhance heme synthesis. PBGS, PBGD, and UROS were fused with the zinc finger proteins ADB1, ADB2, and ADB3 and fixed to the corresponding sequences (scaffold1, scaffold2, and scaffold3, respectively) in the DNA‐guided scaffolds. In addition, the orders of binding sites were optimized for assembly^[^ ^36^ ^]^ to construct the HEME‐S2 to HEME‐S7 strains (Figure 4b). The results demonstrated that heme was synthesized to a higher concentration (0.9 ± 0.0 mg L^−1^) when the binding sites were arranged as scaffold1‐scaffold3‐scaffold2 in the HEME‐S3 strain (Figure 4b). In addition, the different ratios of scaffold1, scaffold3, and scaffold2 (2:1:1; 1:2:1 and 1:1:2) were examined in the HEME‐S8 to HEME‐S10 strains (Figure 4b). The highest concentration of heme (1.4 ± 0.0 mg L^−1^) was observed in the HEME‐S8 strain (Figure 4b). Then, the effect of improving heme synthesis on the whole‐cell activity of BM3_mut_ was examined. However, compared with the control strain C41‐pRSF‐BM3_mut_, the whole‐cell activity of the HEME‐S8‐BM3_mut_ strain was 51.7% lower (Figure S3, Supporting Information). Furthermore, the results of SDS‐PAGE showed that the expression of the BM3_mut_ gene was significantly affected by the episomal overexpression of the key rate‐limiting enzymes in the heme synthetic pathway (Figure S3, Supporting Information).

To relieve the adverse effect on the expression of the BM3_mut_ gene, the fragment of pT7‐*hemA*‐*hemL*‐pT7‐*scaffold1‐scaffold1‐scaffold3‐scaffold2* was integrated into the *cheW* locus in the C41(DE3) strain (Figure 4c). As the expression of *hemA*‐*hemL* and *hemH*‐*hemB*‐*hemC*‐*hemD* genes should be maintained at a proper ratio for heme synthesis,^[^ ^10^ ^]^ the fragment of pT7‐*hemH*‐pT7‐*ADB1‐hemB‐ADB3‐hemD‐ADB2‐hemC* was randomly inserted into the genome using CRISPR‐associated transposases. Subsequently, the strains with different integrated copies of *hemH*‐*hemB*‐*hemC*‐*hemD* genes were selected (HEME‐S11, 7 copies; HEME‐S12, 8 copies; HEME‐S13, 9 copies; HEME‐S14, 10 copies; HEME‐S15, 11 copies) (Figure 4c and Figure S4, Supporting Information). Although the intracellular concentration of heme was reduced (0.2–0.4 mg L^−1^) when *hemA*‐*hemL* and *hemH*‐*hemB*‐*hemC*‐*hemD* genes were integrated into and expressed from the chromosome (Figure 4d), the expression of the BM3_mut_ gene could be nearly recovered (Figure S3, Supporting Information). Additionally, the highest whole‐cell activity increased ‐fold in the HEME‐S13‐BM3_mut_ strain compared with the control strain C41‐pRSF‐BM3_mut_ (Figure 4e). Furthermore, the whole‐cell activity of BM3_mut_ exhibited a trend of increasing first and then decreasing with the further increase of intracellular heme concentration (Figure 4d,e). These results suggest that the intracellular heme supply should be maintained at an optimal level to maximize the activities of P450s.

### 2.4 Dynamically Regulating Heme Synthesis by the Optimized Fine‐Tuned System

**Figure 5:**
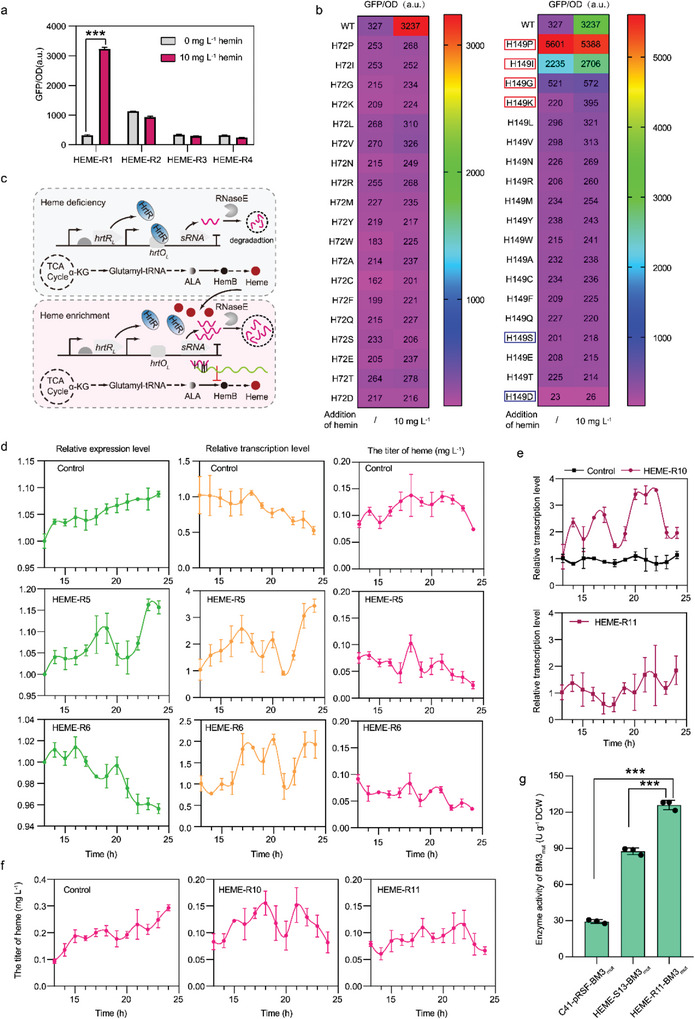
Dynamically regulating heme biosynthesis by an optimized fine‐tuned system. a) The effect of heme monitoring systems from different sources in the C41(DE3) strain. The HEME‐R1 strain contains HrtR_L_ regulator and its binding site *hrtO_L_* from *L. lactis*. The HEME‐R2 strain contains FhtR regulator and its binding site *hrtO_E_* from *E. faecalis*. The HEME‐R3 strain contains HssS_S_ heme sensor, HssR_S_ regulator, and the binding site *hrtO_S_* from *S. aureus*. The HEME‐R4 strain contains HssS_B_ heme sensor, HssR_B_ regulator, and the binding site *hrtO_B_* from *B. anthracis*. The *eGFP* gene was expressed under the control of the *trc‐hrtO* hybrid promoter. b) The intensity heatmap of EGFP regulated by HrtR saturation mutant library. Red box, mutants used to construct heme fine‐tuned system in this study; blue box, the better mutants used to construct the heme dynamic regulation system for ALA production in the previous study.^[^ ^23^ ^]^ c) A fine‐tuned heme biosynthesis system by combining heme biosensor with small regulatory RNA systems. d) Regulation of the expression and transcription level of the *eGFP* gene, and the concentration of heme in recombination strains. The expression levels and transcription levels of *eGFP* were normalized by the expression level and transcription level at 13 h, respectively. The C41(DE3)‐P_J23101_‐EGFP strain was used as a control strain. e) Regulation of *hemB* at the transcriptional level by the fine‐tuned heme biosynthesis system. The transcription levels of *hemB* were normalized by the transcription level at 13 h. The HEME‐S13 strain was used as a control strain. f) The concentration of heme in the recombination strains containing the fine‐tuned heme biosynthesis system. The HEME‐S13 strain was used as a control strain. g) The effect of fine‐tuned heme biosynthesis system on the whole‐cell activity of BM3_mut_. Catalytic conditions for BM3_mut_: 10 OD_600_ mL^−1^ of cells in potassium phosphate buffer (pH 8.0, 100 mm, 0.5 mL), 0.8 mm of 7‐ethoxycoumarin, 28 °C, 220 rpm, 1 h. Data presented as mean values ± SD from three independent biological replicates (*n* = 3). Black circles represent individual data points. Two‐tailed‐Student's t‐test evaluated significance. ***p < 0.001.

To achieve optimal heme supply, it is necessary to develop a system for the fine‐tuned synthesis of heme, including biosensor and transcriptional switcher (Figure 1d). To date, only the heme monitoring system from *L. lactis* (HrtR regulator and its binding site *hrtO_L_*) was used to regulate the level of heme in *E. coli*.^[^ ^37^ ^]^ However, there are many other heme monitoring systems in bacteria, including one‐component systems (FhtR regulator and its binding site *hrtO_E_* from *Enterococcus faecalis*)^[^ ^38^ ^]^ and two‐component systems (HssS_S_ heme sensor, HssR_S_ regulator and its binding site *hrtO_S_* from *Staphylococcus aureus* or HssS_B_ heme sensor, HssR_B_ regulator and its binding site *hrtO_B_* from *Bacillus anthracis*).^[^ ^39^, ^40^ ^]^ All these regulators were expressed in the C41(DE3) strain harboring pCDF‐P_J23117_‐ChuA to examine their usefulness in developing fine‐tuned heme biosynthesis systems (**Figure** **5**a). Though the detection of fluorescence intensity of EGFP expressed under the control of the *trc*‐*hrtO* hybrid promoter in the HEME‐R1 to HEME‐R4 strains, the significant changes in fluorescence intensity (9.9‐fold) could only be observed in the HEME‐R1 strain harboring the heme monitoring system from *L. lactis* after being supplemented with 10 mg L^−1^ hemin (Figure 5a). This indicates that the HrtR regulator and its binding site *hrtO_L_* are the most suitable biosensor for developing the fine‐tuned heme biosynthesis system in *E. coli*.

Next, the sensitivity of HrtR was optimized because the HrtR‐based biosensor has never been used in the heme‐supply enhancing strains. To obtain different mutants of HrtR for fine‐tuning heme biosynthesis, two crucial heme‐binding residues in HrtR (His‐72 and His‐149) were selected for saturation mutagenesis.^[^ ^37^ ^]^ The results showed that all mutants at His‐72 were insensitive to heme (Figure 5b), indicating that the substitution of His‐72 rendered HrtR more resistant to dissociation from the *hrtO_L_* site. Among the mutants at His‐149, the fluorescence intensity increased (1.8‐fold) in the HEME‐R1/H149K strain when supplemented with 10 mg L^−1^ hemin. However, the extent of the increase was significantly lower than that in the HEME‐R1 strain (9.9‐fold) (Figure 5b). These results suggest that HrtR_H149K_ can respond to the intracellular heme level, but its sensitivity was lower than that of wild‐type HrtR. In addition, the fluorescence intensity of the HEME‐R1/H149I, HEME‐R1/H149P, and HEME‐R1/H149G strains was increased by 6.8‐, 17.1‐, and 1.6‐folds, respectively, compared with the HEME‐R1 strain with no hemin supplementation (Figure 5b). These results suggest that HrtR_H149I_, HrtR_H149P_, and HrtR_H149G_ were easier to dissociate from the *hrtO_L_* site. The sensitivities of HrtR_H149I_, HrtR_H149P_, and HrtR_H149G_ were higher than that of wild‐type HrtR at lower intracellular heme levels. Hence, the wild‐type HrtR and its variants (HrtR_H149K_, HrtR_H149I_, HrtR_H149P_, and HrtR_H149G_) were used to construct the fine‐tuned regulatory system for heme synthesis.

Besides the heme biosensor, the transcriptional switcher is another crucial component to develop a fine‐tuned heme biosynthesis system. Thus, we developed a heme biosensor combined with a small regulatory RNA system to avoid the adverse effects of expressing dCas9 gene on cell morphology and to shorten the long regulatory time of CRISPRi (Figure 5c). The effect of small regulatory RNA system was evaluated by employing *eGFP* gene expressed under a constitutive P_J23101_ promoter as a reporter (pET‐P_J23101_‐EGFP) and transcribing sRNA_EGFP_ (targeting *eGFP* gene) under the control of the *trc*‐*hrtO* hybrid promoter. Plasmids pET‐P_J23101_‐EGFP and pACYC‐sRNA_EGFP_‐R1–R5 were co‐expressed in the C41(DE3) strain to construct the HEME‐R5 to HEME‐R9 strains, respectively. ALA (200 mg L^−1^) was added in the medium at the time of inoculation to mimic heme homeostasis during fermentation.

At the early growth stage when the intracellular titer of heme was low,^[^ ^23^ ^]^ the *eGFP* gene was continuously expressed because the transcription of sRNA_EGFP_ was repressed by the binding of constitutively expressed HrtR to the *HrtO_L_* site. Along with the biosynthesis of heme using the supplemented ALA and utilization of heme for cell growth, the fluorescence intensity of EGFP exhibited periodic changes during 13–24 h in the HEME‐R5 strain harboring pET‐P_J23101_‐EGFP and pACYC‐sRNA_EGFP_‐R1: HrtR and HEME‐R6 strain harboring pET‐P_J23101_‐EGFP and pACYC‐sRNA_EGFP_‐R2: HrtR_H149K_ (Figure S5, Supporting Information, and Figure 5d). These results indicate that the intracellular heme can reversibly interact with HrtR or HrtR_H149K_ to periodically derepress the transcription of sRNA_EGFP_, resulting in the fluctuating expression of *eGFP*. Notably, the fluctuating period of fluorescence intensity was between 4–5 h, significantly shorter than the period (longer than 10 h)^[^ ^23^ ^]^ observed using CRISPRi regulation. Thus, the expression of the *eGFP* gene was more efficiently regulated using sRNA_EGFP_ and HrtR or HrtR_H149K_.

The changes in *eGFP* transcription in the HEME‐R5 and HEME‐R6 strains were also examined by RT‐PCR. Using the C41(DE3)‐P_J23101_‐EGFP strain as control and normalizing the transcription levels of *eGFP* in the HEME‐R5 and HEME‐R6 strains by the transcription level at 13 h, the transcriptional fluctuations were observed in both strains from 13 to 24 h (Figure 5d). Similar to the fluctuating period in the expression of *eGFP* gene, the regulatory periods of transcription were 2–3 h in the HEME‐R5 and HEME‐R6 strains, which was again shorter than the period (longer than 6 h)^[^ ^23^ ^]^ regulated by CRISPRi system. After the successful regulation of *eGFP* transcription, the fine‐tuned regulation of *hemB* was investigated in the HEME‐S13 strain capable of best heme biosynthesis. Plasmids pACYC‐sRNA_HemB_‐HrtR and pACYC‐sRNA_HemB_‐HrtR_H149K_ were transformed into the HEME‐S13 strain to construct the HEME‐R10 and HEME‐R11 strains. As in the *eGFP* experiments, the fluctuations in the transcription of *hemB* appeared from 13 to 24 h based on the regulation of heme biosynthesis in the HEME‐R10 and HEME‐R11 strains. Additionally, the regulatory periods of *hemB* (2–3 h) were nearly the same as that of *eGFP* (Figure 5e). However, all the transcriptional levels of *hemB* between 13–24 h were higher than the transcriptional value at 13 h in the HEME‐R10 strain, while the transcription of *hemB* fluctuated above and below the transcriptional value at 13 h in the HEME‐R11 strain from 13–24 h. Thus, the intracellular heme supply was better controlled in the HEME‐R11 strain due to the fine‐tuned regulation of sRNA_HemB_ and HrtR_H149K_ (Figure 5f). Furthermore, using 7‐ethoxycoumarin as a substrate, the whole‐cell enzyme activity of the HEME‐R11‐BM3_mut_ strain increased to 125.9 ± 3.9 U g^−1^ DCW, which was 1.4‐fold higher than that (87.6 ± 2.8 U g^−1^ DCW) obtained with the HEME‐S13‐BM3_mut_ strain and 4.3‐fold higher than that (29.4 ± 1.5 U g^−1^ DCW) obtained with the C41(DE3)‐pRSF‐BM3_mut_ strain (Figure 5g). Thus, the HEME‐R11 strain was selected as the best platform strain for whole‐cell P450 biocatalysis.

### 2.5 The Applications of Fine‐Tuned Heme Biosynthesis System for Whole‐Cell P450 Biocatalysis

**Figure 6:**
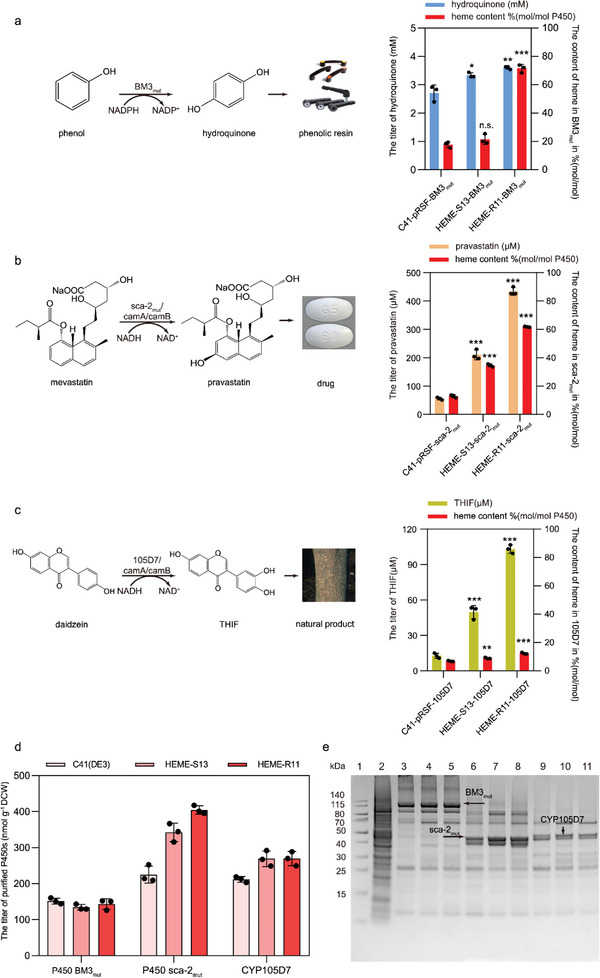
Applications of engineered *E. coli* strains with fine‐tuned heme biosynthesis for efficient whole‐cell P450 biocatalysis. a) The synthesis of chemical intermediate (hydroquinone) using whole‐cell BM3_mut_ biocatalyst. Catalytic conditions for P450 BM3_mut_: 10 OD_600_ mL^−1^ of cells in potassium phosphate buffer (pH 8.0, 100 mm, 0.05 g mL^−1^ glucose, 2 mL), 10 mm of phenol, 30 °C, 220 rpm, 1 h (Selection of reaction time based on Figure S7a, Supporting Information). b) The synthesis of a cholesterol‐lowering drug (pravastatin) using whole‐cell sca‐2_mut_ biocatalyst. Catalytic conditions for P450 sca‐2_mut_: 30 OD_600_ mL^−1^ of cells in potassium phosphate buffer (pH 8.0, 100 mm, 10% v/v glycerol, 2 mL), 0.73 mm of mevastatin, 30 °C, 220 rpm, 12 h (selection of reaction time based on Figure S7b, Supporting Information). c) The synthesis of natural product (THIF, 7,3′,4′‐trihydroxyisoflavone) using whole‐cell CYP105D7 biocatalyst. Catalytic conditions for CYP105D7: 30 OD_600_ mL^−1^ of cells in potassium phosphate buffer (pH 8.0, 100 mm, 10% v/v glycerol, 2 mL), 0.40 mm of daidzein, 30 °C, 220 rpm, 12 h (selection of reaction time based on Figure S7c, Supporting Information). d) The titer of purified P450s expressed in the original C41(DE3) strain, the HEME‐S13 strain, and the HEME‐R11 strain. e) SDS‐PAGE analysis of P450 BM3_mut_, P450 sca‐2_mut_, and CYP105D7 expressed in various recombinant *E. coli* strains. Lane 1: protein standard marker, Lane 2: C41(DE3) strain, Lane 3: C41‐pRSF‐BM3_mut_ strain, Lane 4: HEME‐S13‐BM3_mut_ strain, Lane 5: HEME‐R11‐BM3_mut_ strain, Lane 6: C41‐pRSF‐sca‐2_mut_ strain, Lane 7: HEME‐S13‐sca‐2_mut_ strain, Lane 8: HEME‐R11‐sca‐2_mut_ strain, Lane 9: C41‐pRSF‐105D7 strain, Lane 10: HEME‐S13‐105D7 strain, Lane 11: HEME‐R11‐105D7 strain. Data presented as mean values ± SD from three independent biological replicates (*n* = 3). The heme content in P450s (mol heme/mol P450) indicates the ratio of holo‐P450 in the total purified P450 enzyme. Black circles represent individual data points. Two‐tailed‐Student's t‐test evaluated significance and *p* > 0.05 presents no significance (n.s.). *p < 0.05, **p < 0.01, ***p < 0.001.

Having developed the suitable expression system for P450s together with the enhanced and fine‐tuned heme biosynthesis system, whole‐cell P450 biocatalysts were prepared to synthesize chemical building blocks, drugs, or natural products (Figure 1e). The catalytic performances of whole‐cell P450 biocatalysts were evaluated using the original C41(DE3) strain, the HEME‐S13 strain with enhanced heme biosynthesis, and the HEME‐R11 strain with fine‐tuned heme biosynthesis. The gene encoding the variant of self‐sufficient P450 BM3_mut_ was first expressed using pRSFDuet‐1. The activity of P450 BM3_mut_ was examined for different induction times, and the whole‐cell P450 BM3_mut_ biocatalyst was obtained after 20 h of induced expression (Figure S6a, Supporting Information). A total of 3.6 ± 0.1 mm of hydroquinone, an important chemical and pharmaceutical intermediate of antioxidants and polymers,^[^ ^41^ ^]^ was produced using phenol as a substrate by the HEME‐R11‐BM3_mut_ strain, which was 1.1‐fold and 1.4‐fold higher than that (3.3 ± 0.1 mm) produced by the HEME‐S13‐BM3_mut_ strain and that (2.7 ± 0.3 mm) by the C41‐pRSF‐BM3_mut_ strain, respectively (**Figure** **6**a). The expression levels of BM3_mut_ were similar in the C41(DE3) strain, the HEME‐S13 strain, and the HEME‐R11 strain (Figure 6d,e). To determine the reason for the enhanced yield, the ratio of holo‐BM3_mut_ (BM3 binding with heme) to the total intracellular BM3_mut_ was calculated. The result showed that the ratio of holo‐BM3_mut_ (mol heme/mol BM3_mut_) increased from 17.7% in the C41‐pRSF‐BM3_mut_ strain to 21.5% in the HEME‐S13‐BM3_mut_ strain and to 71.5% in the HEME‐R11‐BM3_mut_ strain (Figure 6a), contributing to the higher amount of active holo‐BM3_mut_ in the cells and the improving the efficiency of whole‐cell BM3_mut_ catalyst.

Besides BM3_mut_, a three‐component P450 catalytic system (sca‐2_mut_ with ferredoxin reductase CamA and ferredoxin CamB) was constructed in the HEME‐R11‐sca‐2_mut_ strain. The activity of sca‐2_mut_ was examined for different induction times, and the whole‐cell sca‐2_mut_ biocatalyst was obtained after 20 h of induced expression (Figure S6b, Supporting Information). The ratio of holo‐sca‐2_mut_ (mol heme/mol sca‐2_mut_) increased from 12.9% in the C41‐pRSF‐sca‐2_mut_ strain to 34.8% in the HEME‐S13‐sca‐2_mut_ strain and to 61.8% in the HEME‐R11‐sca‐2_mut_ strain (Figure 6b). In addition, the titer of purified sca‐2_mut_ increased by 52.1% in the HEME‐S13‐sca‐2_mut_ strain (342.2 ± 25.5 nmol g^−1^ DCW) and 79.7% in the HEME‐R11‐sca‐2_mut_ strain (404.5 ± 11.4 nmol g^−1^ DCW) (Figure 6d,e). The expression level and the heme incorporation of sca‐2_mut_ were improved in the HEME‐R11 strain. Using the HEME‐R11‐sca‐2_mut_ strain, the whole‐cell conversion efficiency (68.4%) of pravastatin, an essential cholesterol‐lowering drug, was achieved using mevastatin as a substrate (Figure 6b), which was 7.9‐fold higher than that (8.7%) obtained with the C41‐pRSF‐sca‐2_mut_ control strain and 1.3‐fold higher than the previously reported conversion efficiency in *E. coli* (53.9%).^[^ ^42^ ^]^

Furthermore, another three‐component P450 catalytic system (CYP105D7 with ferredoxin reductase CamA and ferredoxin CamB) was constructed in the HEME‐R11‐105D7 strain to synthesize natural product 7,3′,4′‐trihydroxyisoflavone (THIF) with anti‐oxidant, anti‐inflammatory and anti‐carcinogenic activities using daidzein as a substrate. The whole‐cell 105D7 biocatalyst was obtained after 20 h of induced expression (Figure S6c, Supporting Information). As a result, the ratio of holo‐105D7 (mol heme/mol 105D7) increased from 6.8% in the C41‐pRSF‐105D7 strain to 8.8% in the HEME‐S13‐105D7 strain and 12.2% in the HEME‐R11‐105D7 strain (Figure 6c). The titer of purified CYP105D7 increased by 27.3% and 27.3% in the HEME‐S13‐105D7 strain (269.3 ± 21.7 nmol g^−1^ DCW) and the HEME‐R11‐105D7 strain (269.3 ± 19.6 nmol g^−1^ DCW), respectively (Figure 6d,e). The expression level and the heme incorporation of CYP105D7 were improved in the HEME‐R11 strain. Furthermore, the highest whole‐cell conversion efficiency of THIF reached 26.2% in the HEME‐R11‐105D7 strain (Figure 6c), which was 8.1‐fold higher than that (3.2%) obtained with the C41‐pRSF‐105D7 control strain and significantly higher than the previously reported conversion efficiency in *E. coli* (1%).^[^ ^43^ ^]^

## 3 Discussion

In recent years, whole‐cell P450 catalysts have attracted increasing attention for their diverse applications in synthesizing chemical compounds^[^ ^3^ ^]^ and natural products.^[^ ^1^ ^]^ However, most studies focused on the discovery and mining of novel P450s,^[^ ^44^ ^]^ the improvement of P450 catalytic properties,^[^ ^45^ ^]^ and enhancing expression levels in microbial hosts.^[^ ^46^ ^]^ For most enzymes, a sufficient supply of essential cofactors is vital for their catalytic efficiencies.^[^ ^47^ ^]^ In the cases of P450s, the apoenzymes will completely lose their activities and are prone to aggregation or degradation when the level of intracellular heme is insufficient.^[^ ^18^ ^]^ Thus, increasing the heme supply to an optimal level is essential to enhance the efficiencies of whole‐cell P450 catalysts. Although the enhancement of heme transport or biosynthesis has previously been attempted,^[^ ^15^, ^19^ ^]^ their effects on the whole‐cell P450 biocatalysts have never been compared. In this work, we report that the effect of enhancing heme transport is rather limited and the improvement of heme biosynthesis is more effective to increase the overall heme supply.

As there are significant differences in the biosynthetic pathways of heme, the feasible strategies for enhancing heme biosynthesis vary considerably in different microorganisms.^[^ ^48^, ^49^ ^]^ In our previous work, all genes involved in heme biosynthesis were overexpressed to achieve the highest titer of heme in *E. coli*.^[^ ^10^ ^]^ However, this strategy is not suitable for whole‐cell P450 biocatalysis as it causes an excessive metabolic burden to the host. Based on the rate‐limiting steps identified in previous studies,^[^ ^10^, ^50^ ^]^ heme biosynthesis was efficiently enhanced by the chromosomally integrated expression of necessary biosynthesis genes (*hemA*, *hemL*, *hemB*, *hemC*, *hemD*, and *hemH*) at proper ratios and the assembly of rate‐limiting enzymes (HemB, HemC, and HemD) by DNA‐guided scaffolds. In addition, heme biosynthesis was controlled in real‐time by a fine‐tuned regulatory system to balance the expression of P450 genes. Although the heme‐sensitive biosensor (mutated HrtR_H149D_ or HrtR_H149S_ regulator and its binding site *hrtO* from *L. lactis*) and CRISPRi have previously been used to regulate the transcription of *hemB*,^[^ ^23^ ^]^ these systems are not suitable for enhancing heme biosynthesis because the sensitivities of HrtR_H149D_ and HrtR_H149S_ regulators are low (Figure 5b) and the regulatory period of transcription of CRISPRi is long. Thus, a new mutant of HrtR (HrtR_H149K_) was selected, and *hemB* transcription^[^ ^23^ ^]^ was regulated by sRNA which allows a shorter regulatory period.

Compared to the original C41(DE3) strain, more ratio of active P450s were available in the HEME‐R11 strain with fine‐tuned heme biosynthesis, resulting in improved catalytic performance (Figure 6). In addition, the expressional levels of three‐component P450 sca‐2_mut_ and CYP105D7 were enhanced in the HEME‐R11 strain (Figure 6d,e). Therefore, the efficient catalytic performances of BM3_mut_, sca‐2_mut_, and CYP105D7 were obtained in the HEME‐R11 strain compared with the previous reported (Table S1, Supporting Information). The catalytic efficiency of BM3_mut_ biocatalyst obtained in the HEME‐R11 strain was increased by 4.3‐fold than that obtained in C41(DE3) strain when using 7‐ethoxycoumarin as a substrate. When using the preferred phenol as a substrate, the catalytic efficiency of BM3_mut_ biocatalyst increased by 1.4‐fold in the HEME‐R11 strain. It indicates that there is a more enhanced catalytic efficiency when using the non‐preferred substrate.

In addition to P450s, the insufficient supply of heme is a common bottleneck for synthesizing other active hemoproteins because heme plays vital roles in their folding, assembly, and function.^[^ ^51^ ^]^ These hemoproteins have been widely applied in the fields of food and medicine, including food processing (soy hemoglobin and porcine myoglobin as coloring and flavoring agents),^[^ ^52^, ^53^ ^]^ food preservation (lactoperoxidase as a bioavailable bacteriostatic agent),^[^ ^54^ ^]^ emergency medicine (human hemoglobin as an acellular oxygen carrier),^[^ ^55^ ^]^ and medical diagnosis (horseradish peroxidase as a chemiluminescent sensor).^[^ ^56^ ^]^ Importantly, hemoglobin and myoglobin have become indispensable components in plant‐based and cell‐based alternative meats.^[^ ^57^ ^]^ Thus, the strategies reported in this paper to develop a fine‐tuned heme biosynthesis system will be useful for developing other whole‐cell biocatalysts involving hemoproteins and also for efficiently producing highly active hemoproteins.

## 4 Experimental Section

### Materials

Primer STAR HS DNA polymerase (Takara, Dalian, China) was used for PCR. Restriction endonucleases and a DNA purification kit were purchased from Thermo Scientific (Waltham, USA). Plasmid and genomic DNA were extracted using the Plasmid Miniprep Purification kit (Sangon Biotech, Shanghai, China) and the TIANGEN pre kit (Tiangen, Beijing, China), respectively. Oligonucleotide synthesis and sequence analyses were performed by Sangon Biotech (Shanghai, China). Hemin and 5‐aminolevulinic acid hydrochloride (ALA) were obtained from Sigma‐Aldrich (St. Louis, MO, USA). Phenol, hydroquinone, mevastatin, pravastatin sodium, daidzein, and 7,3′,4′‐trihydroxyisoflavone were obtained from Macklin (Shanghai, China). Other chemicals obtained were of the highest commercial grade available from Sangon Biotech (Shanghai, China).

### Genetic Manipulation for the Construction of Strains and Plasmids

The plasmids and primers used in this study were listed in Tables S2 and S3, Supporting Information, respectively. *E. coli* DH5*α* strain was used as the DNA cloning host. The *E. coli* BL21(DE3), C41(DE3), and C43(DE3) strains were used to prepare whole‐cell biocatalysts. All strains used in this study were listed in Table S4, Supporting Information. The heterologous gene sequences used in this study were listed in Notes, Supporting Information.

To construct expression system for P450s, BM3_A82F/A328F_ (named BM3_mut_, NCBI Accession Number: P14779.2) from *Bacillus megaterium*,^[^ ^41^ ^]^ sca‐2_G52S/T85F/F89I/T119S/P159A/V194N/D269E/T323A/N363Y/E370V_ (named sca‐2_mut_, NCBI Accession Number: D30815.1) from *Streptomyces carbophilus*,^[^ ^42^ ^]^ CYP105D7 (NCBI Accession Number: BAC75180.2) from *Streptomyces avermitilis*,^[^ ^43^ ^]^ ferredoxin reductase (CamA, NCBI Accession Number: BAA00413.1), and ferredoxin (CamB, NCBI Accession Number: BAA00414.1) from *Pseudomonas putida* ^[^ ^58^ ^]^ were codon‐optimized and synthesized by GenScript (Nanjing, China). *BM3_mut_* and *sca‐2_mut_* were individually subcloned into the *Nde* I*/Xho* I of pRSFDuet‐1 and pETDuet‐1 to construct plasmids pRSF‐BM3_mut_, pET‐BM3_mut_, pRSF‐sca‐2_mut_, and pET‐sca‐2_mut_. To construct sca‐2_mut_ catalytic system, *camA‐camB* genes were subcloned into the *Nco* I*/Sal* I of pRSF‐sca‐2_mut_ and pET‐sca‐2_mut_ to generate plasmids pRSF‐sca‐2_mut_‐CamA‐CamB and pET‐sca‐2_mut_‐CamA‐CamB, respectively. To construct 105D7 catalytic system, *105D7* and *camA‐camB* genes were subcloned into the *Nde* I*/Xho* I and *Nco* I*/Sal* I of pRSFDuet‐1, respectively, to generate plasmid pRSF‐105D7‐CamA‐CamB.

To construct heme import system, the genes *chuA* (NCBI Accession Number: WP_000089583.1) from *E. coli* Nissle 1917 and *phuR* (NCBI Accession Number: AAC13289.1) from *P. aeruginosa* were amplified using the primers ChuA‐F/ChuA‐R and PhuR‐F/PhuR‐R from the genomic DNA of *E. coli* Nissle 1917 and *P. aeruginosa*, respectively. The amplified products were subcloned into the *Nde* I*/Xho* I of plasmid pCDFDuet‐1 to generate plasmids pCDF‐T7‐ChuA and pCDF‐T7‐PhuR. The constitutive expression of heme import system (plasmids pCDF‐P_J23100_‐ChuA, pCDF‐P_J23116_‐ChuA, pCDF‐P_J23117_‐ChuA, pCDF‐P_J23100_‐PhuR, pCDF‐P_J23116_‐PhuR, and pCDF‐P_J23117_‐PhuR) was obtained by replacing T7*lac* promoters with the constitutive promoters (P_J23100_, P_J23116_, and P_J23117_) using the primers (Table S2, Supporting Information) by Gibson assembly.^[^ ^59^ ^]^

To examine the efficiency of heme uptake, C41ΔhemA strain was constructed by deleting the *hemA* gene in C41(DE3) strain using the one‐step gene inactivation method^[^ ^60^ ^]^ and a final concentration of 100 mg L^−1^ ALA was added to the medium to maintain the growth of the C41ΔhemA strain. To obtain the integrated expression of *chuA* gene (HEME‐T7 strain), gene *P_J23117_‐chuA* was integrated into the *adhE* locus of strain C41ΔhemA chromosome using the modified CRISPR/Cas9 system.^[^ ^61^ ^]^

To construct the genetically encoded ratiometric fluorescent sensors HS1, *mKATE2* and *eGFP* genes were amplified using primers mKATE2‐F/mKATE2‐R and EGFP‐mKATE2‐F/EGFP‐mKATE2‐R, respectively. The amplified products were fused by overlap extension PCR^[^ ^62^ ^]^ and the resulting products were cloned into *Nco* I*/BamH* I of pACYCDuet‐1 to generate plasmid pACYC‐mKATE2‐EGFP. Cyt *b_562_* gene was obtained by PCR using primers Cyt b_562_‐F/Cyt b_562_‐R with genomic DNA of *E. coli* C41(DE3) as the template and the backbone fragment was obtained by PCR using primers HS1‐F/HS1‐R with plasmid pACYC‐mKATE2‐EGFP as the template. The resulting PCR products were assembled using Gibson assembly to generate plasmid HS1. To construct the moderate affinity heme sensor HS1‐M7A, the fragments were obtained by PCR using primers HS1‐M7A‐F/HS1‐M7A‐R with plasmid HS1 as the template, and the resulting PCR products were assembled using Gibson assembly. To construct the heme sensor HS1‐M7A/H102A, which cannot bind heme and serve as a control, the fragments were obtained by PCR using primers HS1‐M7A‐H102A‐F/HS1‐M7A‐H102A‐R with plasmid HS1‐M7A as a template, and the resulting PCR products were assembled using Gibson assembly.

To enhance the endogenous biosynthetic pathway of heme, feedback‐resistant *E. coli hemA^fbr^‐hemL* was amplified using primers HemAL‐F/HemAL‐R from the plasmid pCDF‐hemAL,^[^ ^10^ ^]^ and then subcloned into *Nco* I*/Not* I of plasmid pACYCDuet‐1 to generate the plasmid pACYC‐hemAL. Next, *hemB*, *hemD*, and *hemC* genes were amplified using primers HemB‐F1/HemB‐R, HemD‐F1/HemD‐R, and HemC‐F1/HemC‐R from the genomic DNA of *E. coli* C41(DE3), respectively. The amplified products (*hemB*, *hemD*, and *hemC*) were fused by overlap extension PCR^[^ ^62^ ^]^ and subsequently subcloned into the *Nde* I*/Xho* I of plasmid pETDuet‐1 to generate plasmid pET‐hemBDC. Then, *hemH* gene was amplified using primers HemH‐F/HemH‐R from the genomic DNA of *E. coli* C41(DE3) and subcloned into the *Nco* I/*BamH* I of plasmid pET‐hemBDC to generate plasmid pET‐hemBDCH.

To assemble three key cascade enzymes (PBGS, PBGD, and UROS) by DNA‐guided scaffolds for the enhancement of heme synthesis, the zinc‐finger proteins ADB1 (RSNR‐RDHT‐VSTR‐QSNI), ADB2 (VSSR‐RSHR‐RSNR‐CSNR), and ADB3 (QSSR‐RSHR‐RHHR‐QTHQ) were used to construct plasmid pET‐ADB1‐hemB‐ADB3‐hemD‐ADB2‐hemC‐hemH (plasmid pET‐ADB‐hemBDC‐hemH).^[^ ^36^ ^]^ For amplifying *ADB1‐hemB*, the genomic DNA of *E. coli* C41(DE3) was used as a template and primers HemB‐F2/HemB‐R were used for the first round of amplification. The first round of PCR product was used as a template and primers HemB‐F3/HemB‐R were used for the second round of amplification. The primers HemC‐F2/HemC‐R, HemC‐F3/HemC‐R were used for amplifying *ADB2‐hemC* fragment and primers HemD‐F2/HemD‐R, HemD‐F3/HemD‐R were used for amplifying *ADB3‐hemD* fragment by the same approach. The amplified products *ADB1‐hemB*, *ADB2‐hemC*, and *ADB3‐hemD* were fused by overlap extension PCR and used as a template. *ADB1‐hemB*/*ADB3‐hemD*/*ADB2‐hemC* was amplified using primers HemB‐F/HemC‐R, and followed by digestion with restriction endonucleases *Nde* I*/Xho* I, and then cloned into plasmid pET‐hemBDCH to generate plasmid pET‐ADB‐hemBDC‐hemH.

The artificial DNA scaffold1(5′‐CAAGCTAGGGAG‐3′), DNA scaffold2 (5′‐GACGAGGGGGTG‐3′), and DNA scaffold3 (5′‐GAAGGGGGGGTA‐3′), which were respectively recognized by ADB1, ADB2, and ADB3, were subcloned into the *Nde* I*/Xho* I of plasmid pACYC‐hemAL to generate plasmid pACYC‐hemAL‐Scaf123 by Gibson assembly.^[^ ^59^ ^]^ The plasmids pACYC‐hemAL‐Scaf132, pACYC‐hemAL‐Scaf213, pACYC‐hemAL‐Scaf231, pACYC‐hemAL‐Scaf312, pACYC‐hemAL‐Scaf321, pACYC‐hemAL‐Scaf1132, pACYC‐hemAL‐Scaf1332, and pACYC‐hemAL‐Scaf1322 were constructed by Gibson assembly using primers listed in Table S2, Supporting Information.

To enhance plasmid‐free heme synthesis, the fragment of pT7‐*hemH*‐pT7‐*ADB1‐hemB‐ADB3‐hemD‐ADB2‐hemC* were multicopy chromosomal integrated using the CRISPR‐associated transposases (MUCICAT).^[^ ^63^, ^64^ ^]^ At first, plasmids pRE57I‐ADB‐hemBDC‐hemH, pTnsABC, and pQCascade were co‐transformed into *E. coli* C41(DE3) by electro‐transformation, and the transformants were obtained by incubation on triple antibiotic LB‐agar plate (50 µg mL^−1^ kanamycin, 100 µg mL^−1^ ampicillin, and 100 µg mL^−1^ streptomycin) for 16 h at 37 °C. In the following, the transformants were induced by incubation on the triple antibiotic LB‐agar plate containing different concentrations of anhydrotetracycline (100 ng mL^−1^ and 1000 ng mL^−1^) for 16 h at 37 °C,^[^ ^64^ ^]^ and the copy numbers of pT7‐*hemH*‐pT7‐*ADB1‐hemB‐ADB3‐hemD‐ADB2‐hemC* in obtained colonies were verified by colony PCR. The obtained strains were plasmid cured using plasmid pCutamp as previously reported.^[^ ^63^ ^]^ Next, the fragment of pT7‐*hemA*‐*hemL*‐pT7‐*scaffold1‐scaffold1‐scaffold3‐scaffold2* was integrated into the *cheW* locus of the obtained strains using the modified CRISPR/Cas9 system.^[^ ^61^ ^]^ The plasmids pTetQCas‐8+IS186 (MC_0101250), pRE57I‐GNAc (MC_0101251), pTnsABC (MC_0101189), and pCutamp (MC_0101104) were obtained from Molecular Cloud.

To construct heme sensor in *E. coli* strain, *hrtR* (NCBI Accession Number: CAL97228.1) from *L. lactis*, *fhtR* (NCBI Accession Number: WP_002381263.1) from *E. faecalis*, *hssR_S_* (NCBI Accession Number: WP_000249491.1) and *hssS_S_* (NCBI Accession Number: WP_000477329.1) from *S. aureus*, and *hssR_B_* (NCBI Accession Number: WP_000781966.1) and *hssS_B_* (NCBI Accession Number: WP_000839504.1) from *B. anthracis* were chosen as candidates and synthesized by GenScript (Nanjing, China) after codon optimization. To construct plasmid pACYC‐R1 (containing HrtR regulator and its binding site *hrtO_L_* from *L. lactis*), the backbone was obtained by PCR using primers ACYC‐F/ACYC‐R with plasmid pACYCDuet‐1 as a template, and the *hrtR* fragment was obtained by PCR using primers HrtR‐F/HrtR‐R with synthetic *hrtR* gene as a template. The *eGFP* gene expressed under the control of *trc*‐*hrtO_L_* hybrid promoter was obtained by PCR using primers (EGFP‐F/EGFP‐R, EGFP‐F1/EGFP‐R, EGFP‐F2/EGFP‐R). These three gene fragments were subsequently assembled together by Gibson assembly to generate plasmid pACYC‐R1.^[^ ^59^ ^]^ To construct plasmid pACYC‐R2 (containing FrtR regulator and its binding site *hrtO_E_* from *E. faecalis*), the backbone was generated by PCR using primers pACYC‐R1‐F/pACYC‐R1‐R with plasmid pACYC‐R1 as a template, and the *fhtR* gene fragment was generated by PCR using primers FhrR‐F1/FhrR‐R1 and FhrR‐F2/FhrR‐R2 with synthetic *frtR* gene as a template. Next, these two gene fragments were assembled by Gibson assembly. Plasmids pACYC‐R3 (containing HssS_S_ heme sensor, HssR_S_ regulator, and its binding site *hrtO_S_* from *S. aureus*) and pACYC‐R4 (containing HssS_B_ heme sensor, HssR_B_ regulator and its binding site *hrtO_B_* from *B. anthracis*) were synthesized by GenScript (Nanjing, China).

To construct the saturation mutant library of HrtR (histidine 72 and histidine 149), the fragments were obtained using the relevant primers (Table S2, Supporting Information) with the plasmid pACYC‐R1 as a template, and then the respective PCR products were assembled using Gibson assembly.^[^ ^59^ ^]^

To construct reporter plasmid pET‐P_J23101_‐EGFP, *eGFP* was amplified using primers T7_EGFP‐F/T7_EGFP‐R and subcloned into the *Nde* I*/Xho* I of plasmid pETDuet‐1 to generate plasmid pET‐T7‐EGFP. T7*lac* promoter was replaced by constitutive promoters P_J23101_ with primers P_J23101__EGFP‐F/P_J23101__EGFP‐R. To construct plasmid pACYC‐sRNA_EGFP_‐R1, the *HrtR* gene was generated by PCR using primers ACYC_HrtR‐F1/ACYC_HrtR‐R1 with plasmid pACYC‐R1 as a template, and *sRNA‐eGFP‐micC* fragment was generated by PCR using primers micC‐F1/micC‐R1with the *sRNA‐eGFP‐micC* gene synthesized by GenScript (Nanjing, China) as a template.^[^ ^65^ ^]^ These two fragments were assembled with Gibson assembly to generate plasmid pACYC‐sRNA_EGFP_‐R1. To construct plasmids pACYC‐sRNA_EGFP_‐R2 to pACYC‐sRNA_EGFP_‐R5, primers H149K‐F/H149K‐R, H149I‐F/H149I‐R, H149P‐F/H149P‐R, and H149G‐F/H149G‐R were used for PCR using plasmid pACYC‐sRNA_EGFP_‐R1 as a template, and then the respective PCR products were assembled using Gibson assembly.

To construct plasmid pACYC‐sRNA_HemB_‐HrtR, two fragments were obtained by PCR using primers ACYC_HrtR‐F2/ACYC_HrtR‐R2 and micC‐F2/micC‐R2, respectively, with plasmid pACYC‐sRNA_EGFP_‐R1 and *sRNA‐HemB‐micC* gene as the templates. Subsequently, these two fragments were assembled by Gibson assembly. To construct plasmid pACYC‐sRNA_HemB_‐HrtR_H149K_, the fragments were obtained by PCR using the primers H149K‐F/H149K‐R with the plasmid pACYC‐sRNA_HemB_‐HrtR as a template and subsequently assembled using Gibson assembly.

### Media and Culture Conditions

Luria‐Bertani (LB) medium (10 g L^−1^ tryptone, 5 g L^−1^ yeast extract, and 10 g L^−1^ NaCl, pH 7.0) was used for routine cloning and seed culture. Terrific Broth (TB) medium (12 g L^−1^ tryptone, 24 g L^−1^ yeast extract, 0.4% v/v glycerol, 0.017 m KH_2_PO_4_, and 0.072 m K_2_HPO_4_) was used to prepare whole‐cell biocatalysts. MR medium (pH 7.0) containing 6.67 g L^−1^ of KH_2_PO_4_, 4 g L^−1^ of (NH_4_)_2_HPO4, 0.8 g L^−1^ of MgSO_4_ 7H_2_O, 0.8 L^−1^ g of citric acid and 5 mL of trace metal solution (0.5 mol of HCl, 2 g L^−1^ of CaCl_2_, 2.2 g L^−1^ of ZnSO_4_ 7H_2_O, 0.5 g L^−1^ of MnSO_4_ 4H_2_O, 1 g L^−1^ of CuSO_4_ 5H_2_O, 0.1 g L^−1^ of (NH_4_)_6_Mo_7_O_24_ 4H_2_O, 0.02 g L^−1^ of Na_2_B_4_O_7_ 10H_2_O and 10 g L^−1^ of FeSO_4_ 7H_2_O) was used for heme production.^[^ ^10^ ^]^ Antibiotics were added as supplements when necessary: kanamycin, 50 µg mL^−1^; ampicillin, 100 µg mL^−1^; streptomycin/spectinomycin, 100 µg mL^−1^; chloramphenicol, 34 µg mL^−1^; and apramycin, 50 µg mL^−1^. Seed cultures were obtained by inoculating colonies of the recombination strain on LB agar plates and then cultivating 5 mL cultures of LB and appropriate antibiotics in 50 mL test tubes in a rotary shaker for 12 h at 37 °C with shaking at 220 rpm. The amount of inoculum was 2% for the fermentation experiments.

The heme import system plasmids were transformed into the C41ΔhemA strain to generate the recombination strains and a final concentration of 100 mg L^−1^ ALA was added to the medium to maintain the growth of the recombination strains. The performance of the heme import system (HEME‐T1 to HEME‐T7 strains) was examined using 24 deep‐well plates containing 2 mL LB medium supplemented with appropriate antibiotics in triplicate (100 µg mL^−1^ streptomycin for HEME‐T1 to HEME‐T6 strains). A final concentration of 10 mg L^−1^ hemin (1 g L^−1^ in DMSO) was added to the medium, while cultures without additives were set as controls. Cell growth (OD_600_) of HEME‐T1 to HEME‐T7 strains was measured after 24 h of incubation at 37 °C.

The performance of heme production in HEME‐S1 to HEME‐S13 strains was examined by conducting flask cultivations in triplicate. A volume of 1 mL of the seed culture was transferred to 250 mL shaking flasks containing 50 mL MR medium supplemented with 100 µg mL^−1^ ampicillin and 34 µg mL^−1^ chloramphenicol. Cultures were then incubated at 37 °C until the OD_600_ reached 0.6‐0.8, and induced with 1 mm *β*‐D‐1‐thiogalactopyranoside (IPTG), followed by further incubation at 30 °C for 24 h. A 1 mL culture sample was then harvested by centrifugation at 14 000 rpm for 10 min followed, and the pellet was collected. The pellet was resuspended into 1 m NaOH and ultrasonicated. The supernatant was then collected by centrifugation at 14 000 rpm for 20 min and used to examine the concentration of heme by HPLC.

The performance of the recombination strains harboring plasmid pCDF‐P_J23117_‐ChuA and heme sensor plasmids were examined using 24 deep‐well plates containing 2 mL LB medium supplemented with 100 µg mL^−1^ streptomycin and 34 µg mL^−1^ chloramphenicol in triplicate. A final concentration of 10 mg L^−1^ hemin (1 g L^−1^ in DMSO) was added to the medium, while cultures without additives were set as controls. The fluorescence intensity of the recombination strains was measured after 24 h of incubation at 37 °C.

The performance of HEME‐R5 to HEME‐R9 strains was examined using triplicates of 96‐well plates containing 200 µL of LB medium supplemented with 100 µg mL^−1^ ampicillin and 34 µg mL^−1^ chloramphenicol. ALA (100 g L^−1^ in ddH_2_O) was added to the culture to give the final concentration of 200 mg L^−1^ to mimic the status of heme homeostasis during fermentation. Cell growth and fluorescence intensity were monitored during culture at 37 °C for 24 h.

The transcription levels of *eGFP* (C41(DE3)‐P_J23101_‐EGFP, HEME‐R5, and HEME‐R6 strains) and *hemB* (HEME‐S13, HEME‐R10, and HEME‐R11 strains) were quantified by conducting flask cultivations in triplicate. For the fermentation of C41(DE3)‐P_J23101_‐EGFP, HEME‐R5, and HEME‐R6 strains, 1 mL of the seed culture was transferred to 250 mL shaking flasks containing 50 mL of LB medium and 200 mg L^−1^ ALA supplemented with 100 µg mL^−1^ ampicillin and 34 µg mL^−1^ chloramphenicol (only 100 µg mL^−1^ampicillin for C41(DE3)‐P_J23101_‐EGFP strain). For the fermentation of HEME‐S13, HEME‐R10, and HEME‐R11 strains, 1 mL of the seed culture was transferred to 250 mL shaking flasks containing 50 mL of MR medium (34 µg mL^−1^ chloramphenicol for HEME‐R10 and HEME‐R11 strains). Cells were cultured at 37 °C until the OD_600_ reached 0.6–0.8 and then induced with 1 mm *β*‐D‐1‐thiogalactopyranoside (IPTG), followed by incubation at 30 °C for 24 h. During the 13–24 h fermentation, samples were collected for quantitative real‐time PCR and determination of intracellular heme by HPLC‐MS.

### Cell Growth and Fluorescence Detection

Cell growth was detected by measuring the optical density at 600 nm (OD_600_) with a spectrophotometer (UVmini‐1240, Shimadzu Corporation, Japan). The EGFP fluorescence signal (excitation, 488 nm; emission, 523 nm) and mKATE2 fluorescence signal (excitation, 588 nm; emission, 620 nm) were measured in 96‐well microtiter plates using a Cytation Microplate Reader (BioTek). The relative fluorescence intensity was calculated with normalization at OD_600_, and all experiments were performed in triplicate.

### Preparation of Whole‐Cell Biocatalysts

To prepare P450BM3_mut_/P450sca‐2_mut_/CYP105D7 whole‐cell biocatalysts, 1 mL of the seed culture was transferred to 250 mL shaking flasks containing 50 mL TB medium supplemented with appropriate antibiotics and was cultured at 37 °C until the OD_600_ reached 0.6–0.8. Cultures were then induced with 1 mm *β*‐D‐1‐thiogalactopyranoside (IPTG) and incubated at 25 °C for 20 h. After cultivation, the cells were harvested by centrifugation at 8000 rpm for 10 min, washed twice with potassium phosphate buffer (100 mm, pH 8.0), and then used for the subsequent biotransformation experiments.

To convert phenol to hydroquinone using P450BM3_mut_ biocatalysts, resting cell pellets were resuspended in potassium phosphate buffer (100 mm, pH 8.0) containing 0.05 g mL^−1^ glucose. Substrate phenol (0.5 m in potassium phosphate buffer) was added to a 2 mL cell suspension to give the final concentration of 10 mm, and reactions were conducted at 30 °C and 220 rpm for 1 h using 24‐well plates. Then, 200 µL of the reaction solution was mixed with 800 µL of methanol, followed by centrifugation at 14 000 rpm for 20 min. The supernatant was used to detect the production of hydroquinone by HPLC analysis and all experiments were performed in triplicate.

To convert mevastatin to pravastatin using P450sca‐2_mut_ biocatalysts, resting cell pellets were resuspended in potassium phosphate buffer (100 mm, pH 8.0) containing 10% (v/v) glycerol. Mevastatin (14 g L^−1^ in 96% ethanol‐water) was prepared by the saponification of lactone in 96% ethanol‐water and 0.1 m NaOH at 50 °C for 2 h with stirring. And the pH value of the substrate was adjusted to 7.0 with 0.1 m HCl.^[^ ^66^ ^]^ Then, 40 µL of the prepared mevastatin sodium was added to 2 mL cell suspensions for reaction. Reactions were conducted at 30 °C and 220 rpm for 12 h using 24‐well plates. Next, 1 mL of the reaction solution was centrifugated at 14 000 rpm for 20 min, and the supernatant was used to detect the production of pravastatin by HPLC. All experiments were performed in triplicate.

To convert daidzein to 7,3′,4′‐trihydroxyisoflavone using CYP105D7 biocatalysts, resting cell pellets were resuspended in potassium phosphate buffer (100 mm, pH 8.0) containing 10% (v/v) glycerol. The daidzein substrate (5 g L^−1^ in DMSO: MeOH = 1:1, v/v) was added to 2 mL of the cell suspension to give a final concentration of 100 mg L^−1^. Reactions were conducted at 30 °C and 220 rpm for 12 h in 24‐well plates. Then, 500 µL of the reaction solution was extracted thrice with 500 µL of ethyl acetate. The products were dried, dissolved in methanol, and then used to detect the production of 7,3′,4′‐trihydroxyisoflavone by HPLC analysis. All experiments were performed in triplicate.

### Protein Purification and Hemochrome Binding Assay

The collected cell pellets were resuspended in wash buffer (20 mL; 100 mm potassium phosphate buffer, pH 8.0, 500 mm NaCl, 20 mm imidazole), and cells were ultrasonicated on ice using an ultrasonic processor (shanghai, China) under the following conditions: power, 38%; 2 s on /3 s off; 5 min. Cell debris was removed by centrifugation at 12 000 rpm for 10 min at 4 °C. Purified protein was obtained by eluting the sample with elution buffer (1 mL; 100 mm potassium phosphate buffer, pH 8.0, 500 mm NaCl, 200 mm imidazole) using Ni‐NTA His‐Binding‐resin (PointBio, Shanghai, China). Following elution, the buffer of obtained enzyme was replaced with potassium phosphate buffer (100 mm, pH 8.0) using an Amicon Ultra 30 K centrifugal filter (Merck). Protein concentration was measured using the Bradford protein Assay Kit (Beyotime Biotech, Shanghai, China). Finally, the heme contents in purified P450 enzymes were measured using a hemochrome binding assay.^[^ ^67^ ^]^

### Determination of Expression Levels of P450s in C41(DE3) Strain, the HEME‐S13 Strain, and the HEME‐11 Strain

The expression levels of P450s (BM3_mut_, sca‐2_mut_, and CYP105D7) were measured in the original C41(DE3) strain, the HEME‐S13 strain with enhanced heme biosynthesis, and the HEME‐R11 strain with fine‐tuned heme biosynthesis. BeaverBeads His‐tag Protein Purification Kit (BeaverBeads, Beaver, Suzhou, China) was used to obtain the purified P450s. At first, 4 mL of collected cell pellets were resuspended in Binding Buffer (4 mL; 100 mm potassium phosphate buffer, pH 8.0, 500 mm NaCl, 5 mm imidazole), and cells were ultrasonicated on ice as before. Cell debris was removed by centrifugation at 12 000 rpm for 10 min at 4 °C. Then, the soluble enzymes were absorbed by 0.5 mL nickel magnetic beads (BeaverBeads, Beaver, Suzhou, China) for 30 min at room temperature. Then the His‐P450s beads were washed three times with the washing buffer (4 mL; 100 mm potassium phosphate buffer, pH 8.0, 500 mm NaCl, 50 mm imidazole). Subsequently, the purified P450s were finally eluted with elution buffer (0.5 mL; 100 mm potassium phosphate buffer, pH 8.0, 500 mm NaCl, 500 mm imidazole). The purified P450s obtained were used for the determination of protein concentration and SDS‐PAGE analysis.

### Determination of Enzyme Activity for BM3

After cultivation, a total of 500 µL of cells was collected by centrifugation at 10 000 rpm for 5 min. The supernatant was discarded, and the cell pellets were resuspended in 500 µL potassium phosphate buffer (100 mm, pH 8.0). 7‐ethoxycoumarin (20 mm in DMSO) was added to the cell suspension at a final concentration of 0.8 mm, and the tube was incubated on a rotary shaker at 220 rpm and 28 °C for 60 min. The supernatant was collected by centrifugation (10 000 rpm, 5 min), and the fluorescence intensity of 7‐hydroxycoumarin was measured using a fluorescent plate reader with an emission wavelength of 458 nm and an excitation wavelength of 398 nm.^[^ ^68^ ^]^ The titer of products was calculated based on the fluorescence intensity and the fluorescence intensity of standard samples of known concentrations. Enzyme activity (U g^−1^ DCW) was defined as µmols of 7‐hydroxycoumarin produced by per g dry cell weight of whole‐cell biocatalyst per minute.

### HPLC/HPLC‐MS Determination

Phenol, hydroquinone, mevastatin, pravastatin sodium, daidzein, and 7,3′,4′‐trihydroxyisoflavone were quantified using HPLC (Shimadzu, Kyoto, Japan) equipped with a ZORBAX Eclipse XDB‐C18 (5 µm, 4.6 × 250 mm, Agilent, USA) and an ultraviolet/VIS detector, SPD‐20A (Shimadzu, Kyoto, Japan). Methanol/acetonitrile and water containing 0.1% trifluoroacetic acid were used as the mobile phase. For analysis of phenol and hydroquinone,^[^ ^41^ ^]^ the parameters were set as follows: a flow rate of 0.6 mL min^−1^; methanol ratio of 45%; column temperature of 35 °C, and monitored absorption wavelengths of 220 nm. For analysis of mevastatin and pravastatin sodium,^[^ ^69^ ^]^ the parameters were set as follows: a flow rate of 0.8 mL min^−1^; methanol ratio of 75%; column temperature of 30 °C, and monitored absorption wavelengths of 237 nm. For analysis of daidzein and 7,3′,4′‐trihydroxyisoflavone,^[^ ^43^ ^]^ the parameters were set as follows: a flow rate of 0.8 mL min^−1^; acetonitrile ratio of 35%; column temperature of 30 °C, and monitored absorption wavelengths of 250 nm.

For analysis of heme by HPLC,^[^ ^10^ ^]^ methanol and water containing 0.1% trifluoroacetic acid were used as the mobile phase. The parameters were set as follows: a flow rate of 0.8 mL min^−1^; methanol ratio of 30% at the first 1 min, 100% at 20 min, 100% at 35 min, and 30% at 37 min; column temperature of 40 °C and monitored absorption wavelengths of 400 nm.

For analysis of heme by HPLC‐MS, 1 µL of samples were loaded onto an Agilent ZORBAX Eclipse Plus C18 column (2.1 × 50 mm, 1.8 µm), separated using Agilent 1290 Infinity II LC system, and quantified by Agilent 6495C triple quadrupole mass spectrometer (Agilent, USA) equipped with electrospray ionization (ESI) interface in the positive ion mode. Elution was performed with mobile A consisting of 10 mm ammonium formate containing 0.1% formic acid and acetonitrile containing 0.1% formic acid as mobile phase B. The rate was set as 0.4 mL min^−1^ and the solvent gradient was adopted as follows for a total run time of 3.2 min: 0.0–0.1 min, isocratic at 10% B; 0.1–0.8 min, 10% to 100% B; 0.8–1.0 min, 100% B; 1.0–2.4 min, 100% B to 60% B; 2.4–2.8 min, 60% B to 10% B; 2.8–3.2 10% B. Mass transition, *m/z* 616.2 → 557.2 was selected to monitor heme. The capillary voltage was set at 2.5 kV. Nitrogen was used as the drying gas at a flow rate of 14 L min^−1^ at 200 °C. The nebulizer pressure was set at 24 psi.

### Quantitative Real‐Time PCR (RT‐PCR)

Total RNA was immediately extracted using the RNAprep Pure Cell/Bacteria Kit (Tiangen, Beijing, China) according to the manufacturer's instructions. The RNA centration was quantified by the 260/280 ratios using a Nanodrop ND‐2000 spectrophotometer (Thermo Scientific, Wilmington, DE, USA). cDNA was synthesized via reverse transcription using a PrimeScript RT reagent Kit (TaKaRa, Dalian, China) following the supplier guidelines. The synthesized cDNA was diluted 20 times prior to the RT‐PCR. The RT‐PCR was conducted in a LightCycler 480 II Real‐time PCR instrument (Roche Diagnostics, Mannheim, Germany) using an SYBR Premix Ex Taq Kit (TaKaRa, Dalian, China) with 20.0 µL reaction consisting of 10.0 µL TB Green *Premix Ex Taq II*, 0.4 µL PCR forward primer, 0.4 µL PCR reverse primer, 1.0 µL DNA template, and 8.2 µL ddH_2_O. The parameters were: pre‐incubation at 95 °C for 30 s; 50 cycles of amplification step at 95 °C for 5 s, 55 °C for 30 s, and 72 °C for 30 s; cooling at 50 °C for 30 s. The gene‐specific primers designed for RT‐PCR using Beacon Designer 7.9 were listed in Table S2, Supporting Information. The *hcaT* (HcaT MFS transporter) gene was used as the reference gene.^[^ ^70^ ^]^ The transcription level at 13 h of target gene was used for normalization and the results were analyzed using the 2^−ΔΔCt^ method.^[^ ^71^ ^]^ All experiments were performed with three biological replicates.

### Statistical Analysis

All experiments were independently carried out at least three times, and the data were displayed as mean values ± standard deviation (SD). Two‐tailed‐Student's t‐test performed statistical data analysis in Excel (Microsoft Office 365). *P* values of <0.05 were considered statistically significant, and statistical significance was indicated as *P < 0.05, **P < 0.01, and ***P < 0.001.

## Conflict of Interest

The authors declare no conflict of interest.

## Author Contributions

B.D.H., H.B.Y., J.W.Z., J.H.L., J.C., G.C.D., S.Y.L., and X.R.Z. designed the project. B.D.H. performed experiments, analyzed the data, and drafted the manuscript. S.Y.L. and X.R.Z. wrote and revised the manuscript.

## Supporting information

Supporting Information

Supporting Information

## Data Availability

The data that support the findings of this study are available in the supplementary material of this article.
