# Supplementary material for: Whole‐Cell P450 Biocatalysis Using Engineered Escherichia coli with Fine‐Tuned Heme Biosynthesis
Source: Adv Sci (Weinh). 2022 Dec 16;10(6):2205580. doi: 10.1002/advs.202205580 (PMC9951570; doi:10.1002/advs.202205580)
Supplement: Supplementary file 1 — Supporting Information [file ADVS-10-2205580-s001.pdf]

Supplementary Information

Whole-cell P450 biocatalysis using engineered *Escherichia coli* with fine-tuned heme biosynthesis

Baodong Hu [1,2,3,4], *Haibo Yu* [1,2,3,4], *Jingwen Zhou* [1,2,3,4], *Jianghua Li* [1,2,3,4], *Jian Chen* [1,2,3,4], *Guocheng Du* [1,2,3,4,5], *Sang Yup Lee*[6], *Xinrui Zhao* [1,2,3,4*]

[1] Key Laboratory of Industrial Biotechnology, Ministry of Education, School of Biotechnology, Jiangnan University, 1800 Lihu Road, Wuxi, Jiangsu 214122, China;

[2] Science Center for Future Foods, Jiangnan University, 1800 Lihu Road, Wuxi, Jiangsu 214122, China;

[3] Jiangsu Province Engineering Research Center of Food Synthetic Biotechnology, Jiangnan University, 1800 Lihu Road, Wuxi, Jiangsu 214122, China;

[4] Engineering Research Center of Ministry of Education on Food Synthetic Biotechnology, Jiangnan University, 1800 Lihu Road, Wuxi, Jiangsu 214122, China;

[5] Key Laboratory of Carbohydrate Chemistry and Biotechnology, Ministry of Education, Jiangnan University, 1800 Lihu Road, Wuxi, Jiangsu 214122, China;

[6] Metabolic and Biomolecular Engineering National Research Laboratory, Department of Chemical and Biomolecular Engineering (BK21 Plus Program), BioProcess Engineering Research Center, Bioinformatics Research Center, and Institute for the BioCentury, Korea Advanced Institute of Science and Technology (KAIST), Yuseong-gu, Republic of Korea

[*] Correspondence: zhaoxinrui@jiangnan.edu.cn

Contents

Supplementary Methods 1

Supplementary Figures 1-7

Supplementary Tables 1-4

Supplementary Notes 1-16

Supplementary Methods

1 Determination of heme uptake using the genetically encoded ratiometric fluorescent sensors.

The performance of heme uptake in the HEME-T4 strain was detected by the genetically encoded ratiometric fluorescent sensors HS1.[1] The high affinity sensor HS1, binding heme using Met and His axial ligands of the cytochrome domain, exhibits dissociation constants of 3 nM for ferric heme ($K_D^{Fe(III)}$) and 1 nM for ferrous heme ($K_D^{Fe(II)}$).[2] The moderate affinity heme sensor HS1-M7A, has the axial methionine ligand mutated to alanine, and exhibits dissociation constants of 2 μM for ferric heme ($K_D^{Fe(III)}$) and 25 nM for ferrous heme ($K_D^{Fe(II)}$).[2] The double mutant HS1-M7A/H102A,[2] has both the methionine and histidine axial ligands mutated to alanine, cannot bind heme and serves as a control for determining heme-independent changes to sensor fluorescence ratios. The intracellular heme can be calculated based on the eGFP/mKATE2 fluorescence ratios in wild-type HS1 and HS1-M7A compared to the non-binding HS1-M7A/H102A.[2,3]

The genetically encoded ratiometric fluorescent sensors HS1, HS1-M7A, and HS1-M7A/H102A plasmids transformed into the HEME-T4 strain, respectively, to generate the HEME-T4-HS1, HEME-T4-HS1$_{M7A}$, and HEME-T4-HS1$_{M7A/H102A}$ strains. Then, these three strains were examined using 24 deep-well plates containing 2 mL LB medium supplemented with 100 μg mL^{-1} streptomycin and 34 μg mL^{-1} chloramphenicol in triplicate. A final concentration of 10 mg L^{-1} hemin (1 g L^{-1} in DMSO) was added to the medium, while cultures without additives were set as

controls. After cultivating 24 h at 37℃, the EGFP fluorescence signal (excitation, 488 nm; emission, 523 nm) and mKATE2 fluorescence signal (excitation, 588 nm; emission, 620 nm) were measured in 96-well microtiter plates using a Cytation Microplate Reader (BioTek).

Supplementary Figures

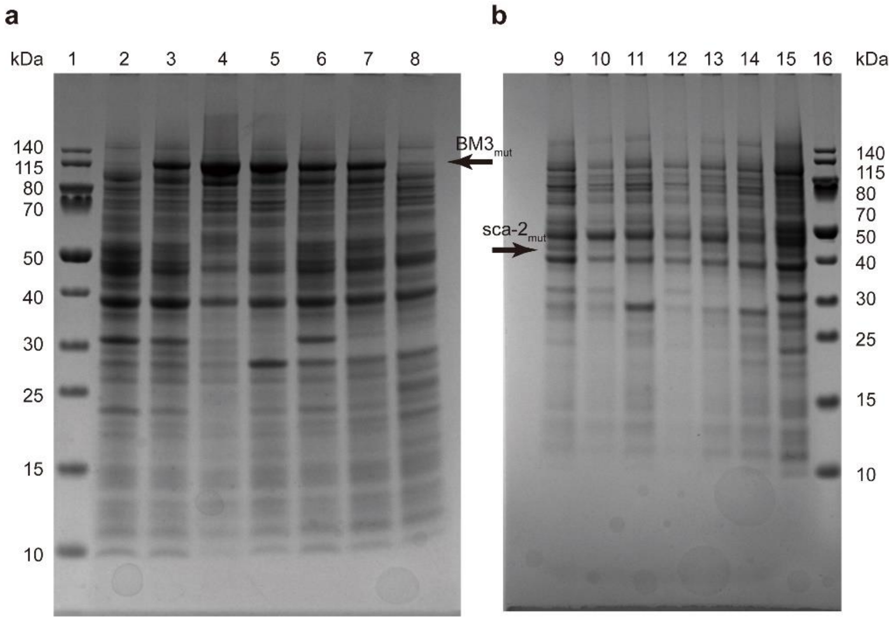

Figure S1 SDS-PAGE analysis of P450s in various expression systems. a) $BM3_{mut}$ enzyme in supernatants of various expression systems. Lane 1: protein standard marker; Lane 2: C41-pRSFDuet-1; Lane 3: C41-pRSF-$BM3_{mut}$; Lane 4: B21-pRSF-$BM3_{mut}$; Lane 5: C43-pRSF-$BM3_{mut}$; Lane 6: C41-pET-$BM3_{mut}$; Lane 7: B21-pET-$BM3_{mut}$; Lane 8: B21-pET-$BM3_{mut}$. b) sca-2_{mut} enzyme in supernatants of various expression system. Lane 9: C41-pRSF-sca-2_{mut}; Lane 10: B21-pRSF-sca-2_{mut}; Lane 11: C43-pRSF-sca-2_{mut}; Lane 12: C41-pET-sca-2_{mut}; Lane 13: B21-pET-sca-2_{mut}; Lane 14: C43-pET-sca-2_{mut}; Lane 15: C41-pRSFDuet-1. Lane 16: protein standard marker.

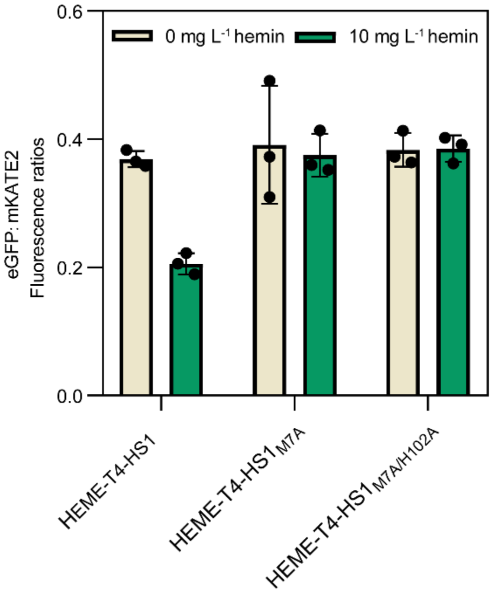

Figure S2 Determination of heme uptake in the HEME-T4 strain by the genetically encoded ratiometric fluorescent sensors. The HEME-T4-HS1 strain contains the high affinity sensor HS1, the HEME-T4-HS1$_{M7A}$ strain contains the moderate affinity heme sensor HS1-M7A, and the HEME-T4-HS1$_{M7A/H102A}$ strain contains the double mutant HS1-M7A/H102A using as a control. Data presented as mean values ± SD from three independent biological replicates (*n*=3). Black circles represent individual data points.

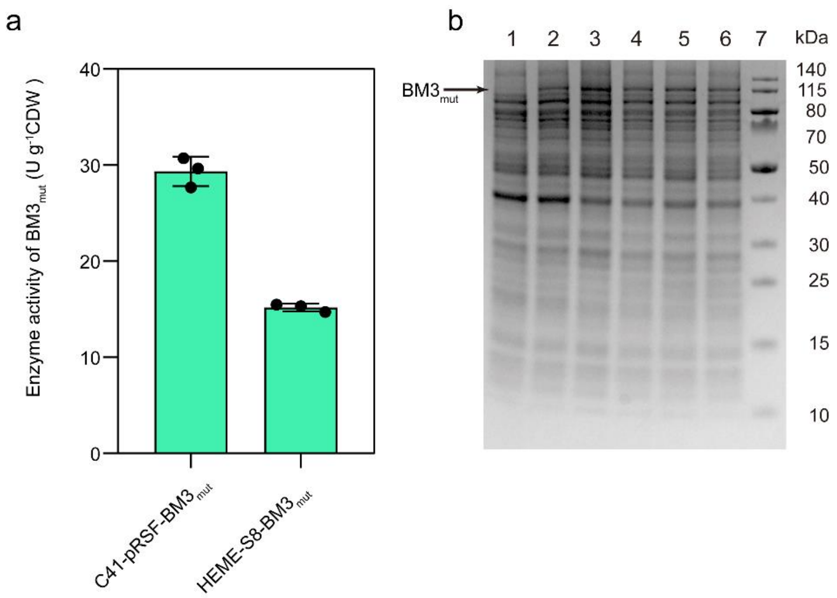

Figure S3 Effect of recombinant *E. coli* strains on the whole-cell activity and expression levels of $BM3_{mut}$. a) Effect of recombinant *E. coli* strains with overexpressing of the necessary synthetic genes on the whole-cell activity of $BM3_{mut}$. The activity of $BM3_{mut}$ biocatalyst using 7-ethoxycoumarin as a substrate. Catalytic conditions for $BM3_{mut}$: 10 OD_{600} of cells in potassium phosphate buffer (pH 8.0, 100 mM, 0.5 mL), 0.8 mM of 7-ethoxycoumarin, 28°C, 220 rpm, 1 h. Data presented as mean values ± SD from three independent biological replicates (n=3). Black circles represent individual data points. b) SDS-PAGE analysis of $BM3_{mut}$ expressed in various recombinant *E. coli* strains. Lane 1: HEME-S8-$BM3_{mut}$, Lane 2: C41-pRSF-$BM3_{mut}$, Lane 3: HEME-S13-$BM3_{mut}$, Lane 4: HEME-R10-$BM3_{mut}$ (harboring plasmid pACYC-$sRNA_{HemB}$-HrtR), Lane 5: HEME-R11-$BM3_{mut}$ (harboring plasmid pACYC-$sRNA_{HemB}$-$HrtR_{H149K}$), Lane 6: HEME-R12-$BM3_{mut}$ (harboring plasmid pACYC-$sRNA_{HemB}$-$HrtR_{H149I}$), Lane 7: protein standard marker.

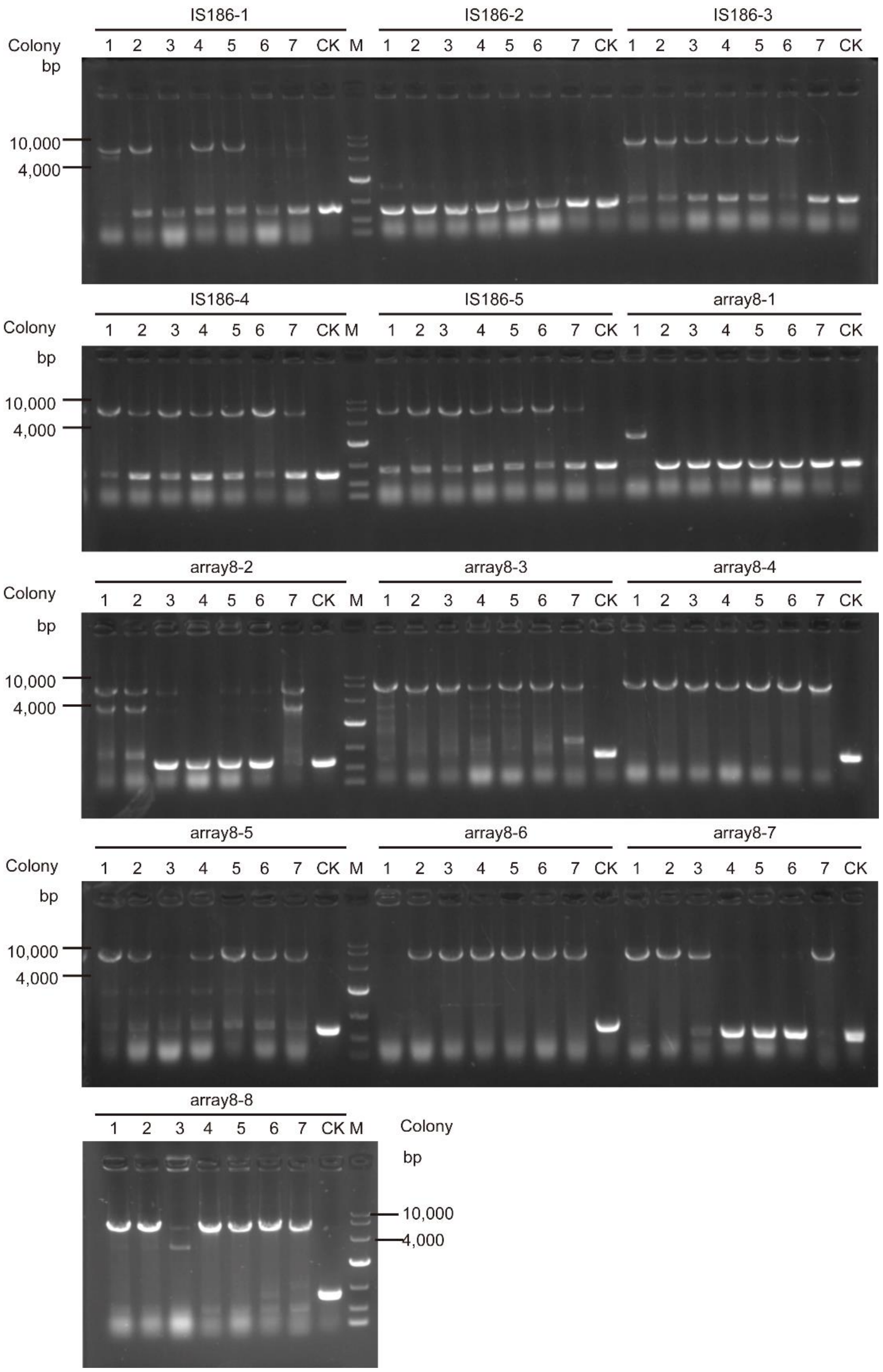

Figure S4 Integration of HemBCDH cargo into IS186 and array8 loci in the C41(DE3) strain based on the CRISPR-associated transposases system. Colony 1, 2, 3, 4 and 6 indicated strains HEME-S11, HEME-S12, HEME-S13, HEME-S14 and HEME-S15, respectively.

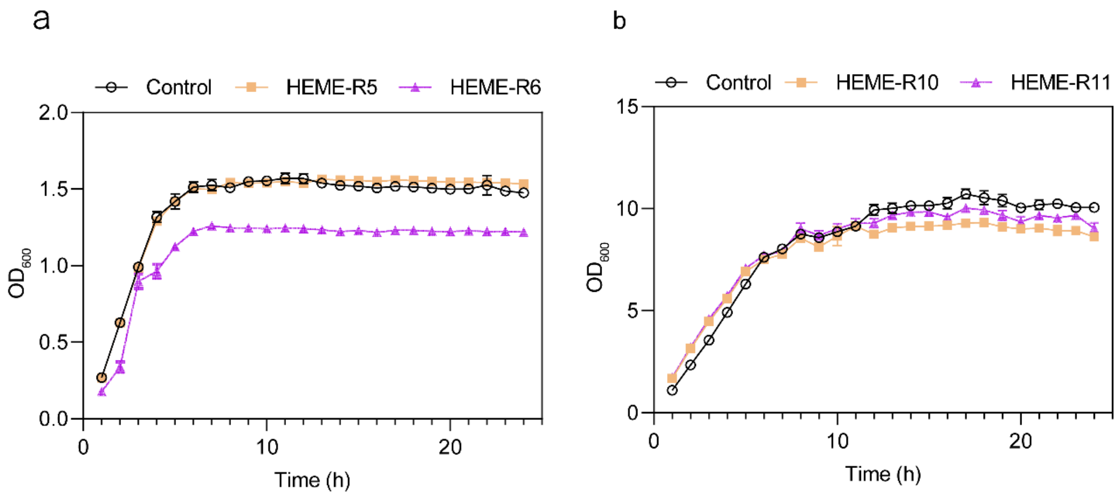

Figure S5 The growth behavior of recombination strains with heme fine-tuned systems. a) The C41(DE3)-P_{J23101}-EGFP strain was used as a control. The control C41(DE3)-P_{J23101}-EGFP strain, the HEME-R5 strain, and the HEME-R6 strain were cultured using the triplicates of 96-well plates and the growth of three strains were measured in 96-well microtiter plates using a Cytation Microplate Microplate Reader (BioTek). b) The HEME-S13 strain was used as a control. The HEME-S13 strain, the HEME-R10 strain, and the HEME-R11 strain were cultured using 250 mL shaking flaking and the cell growth of these three strains were measured using a spectrophotometer (UVmini-1240, Shimadzu Corporation, Japan). Data presented as mean values ± SD from three independent biological replicates (*n*=3). Black circles represent individual data points.

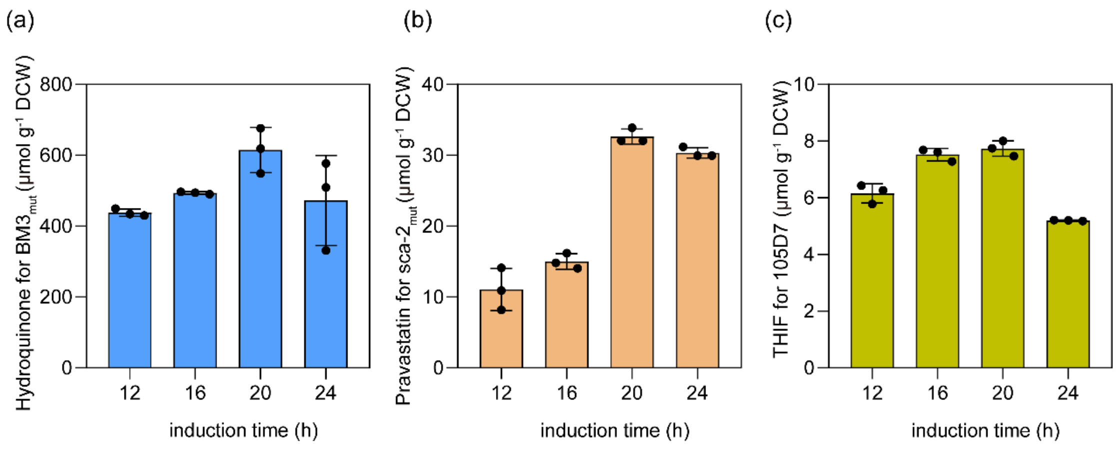

Figure S6 Effect of different induction times on the whole-cell activities of P450 $BM3_{mut}$, P450 sca-2_{mut}, and CYP105D7 in the HEME-R11 strain. a) Catalytic conditions for P450 $BM3_{mut}$: 10 OD_{600} mL^{-1} of cells in potassium phosphate buffer (pH 8.0, 100 mM, 0.05 g mL^{-1} glucose, 2 mL), 10 mM of phenol, 30°C, 220 rpm, 1 h. b) Catalytic conditions for P450 sca-2_{mut}: 30 OD_{600} mL^{-1} of cells in potassium phosphate buffer (pH 8.0, 100 mM, 10% v/v glycerol, 2 mL), 0.73 mM of mevastatin, 30°C, 220 rpm, 12 h. c) Catalytic conditions for CYP105D7: 30 OD_{600} mL^{-1} of cells in potassium phosphate buffer (pH 8.0, 100 mM, 10% v/v glycerol, 2 mL), 0.40 mM of daidzein, 30°C, 220 rpm, 12 h. Data presented as mean values ± SD from three independent biological replicates (*n*=3). Black circles represent individual data points.

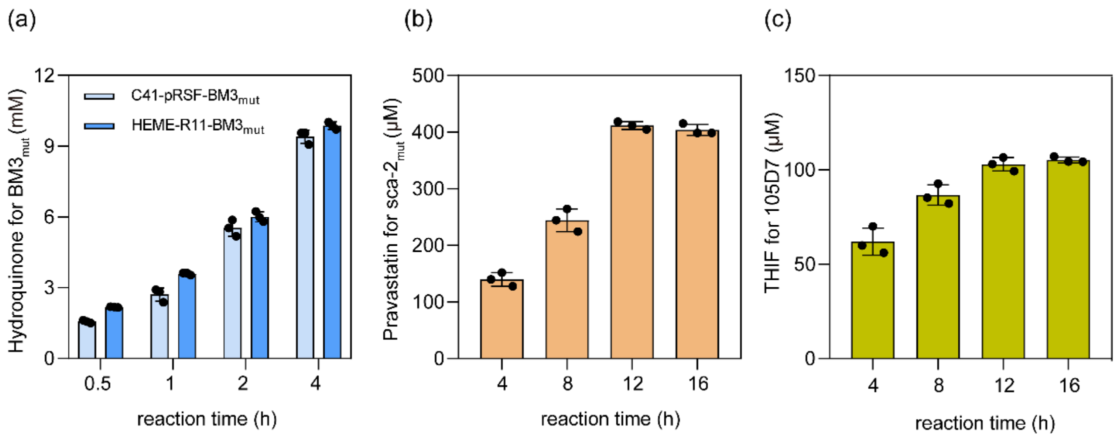

Figure S7 Effect of reaction time on the whole-cell biocatalysis of P450 BM3$_{mut}$, P450 sca-2$_{mut}$, and CYP105D7. a) The C41-pRSF-BM3$_{mut}$ strain and the HEME-R11-BM3$_{mut}$ strain were used for whole-cell biocatalysis. Catalytic conditions for P450 BM3$_{mut}$: 10 OD$_{600}$ mL^{-1} of cells in potassium phosphate buffer (pH 8.0, 100 mM, 0.05 g mL^{-1} glucose, 2 mL), 10 mM of phenol, 30°C, 220 rpm, 1 h. b) The HEME-R11-sca-2$_{mut}$ strain was used for whole-cell biocatalysis. Catalytic conditions for P450 sca-2$_{mut}$: 30 OD$_{600}$ mL^{-1} of cells in potassium phosphate buffer (pH 8.0, 100 mM, 10% v/v glycerol, 2 mL), 0.73 mM of mevastatin, 30°C, 220 rpm, 12 h. c) The HEME-R11-105D7 strain was used for whole-cell biocatalysis. Catalytic conditions for CYP105D7: 30 OD$_{600}$ mL^{-1} of cells in potassium phosphate buffer (pH 8.0, 100 mM, 10% v/v glycerol, 2 mL), 0.40 mM of daidzein, 30°C, 220 rpm, 12 h. Data presented as mean values ± SD from three independent biological replicates (*n*=3). Black circles represent individual data points.

Supplementary Tables

Supplementary Table 1. Comparison of the efficiency of whole-cell catalysis using the HEME-R11 strain with previously reported results.

Comparison of whole-cell catalysis using the HEME-R11 strain

Catalysts	Hosts	Culture conditions	Substrates	Reaction conditions	Conversion rates	References
BM3(A82F/A328F)	*E. coli* BL21(DE3)	TB medium	phenol (10 mM)	30°C, 200 rpm, 5 h	99%	[4]
BM3(A82F/A328F)	HEME-R11	TB medium	phenol (10 mM)	30°C, 220 rpm, 4 h	99%	This study
sca-2_{mut}	*E. coli* BL21(DE3)	LB medium with 1 mM ALA	mevastatin (0.7 mg/mL)	30°C, 250 rpm, 21 h	53.9%	[5]
sca-2_{mut}	HEME-R11	TB medium	mevastatin (0.28 mg/mL)	30°C, 220 rpm, 12 h	68.4%	This study
CYP105D7	*E. coli* BL21(DE3)	LB medium	daidzein (500 μM)	28°C, 280 rpm, 6 h	1%	[6]
CYP105D7	*Streptomyces avermitilis* MA4680	R2YE medium	daidzein (100 μM)	28°C, 280 rpm, 72 h	24%	[6]
Artificial CYP105D7	*E. coli* BL21(DE3)	LB medium with 0.25 mM ALA	daidzein (100 μM)	37°C, 200 rpm, 24 h	2.1%	[7]
Artificial CYP105D7	*Streptomyces avermitilis* MA4680	R2YE medium	daidzein (100 μM)	28°C, 200 rpm, 72 h	34.6%	[7]
CYP105D7	HEME-R11	TB medium	daidzein (394 μM)	30°C, 220 rpm, 12 h	26.2%	This study

Supplementary Table 2. Plasmids used in the study. Abbreviations: Ap, ampicillin; Km, kanamycin; Cm, chloramphenicol; Sm, streptomycin/spectinomycin; Am, apramycin; R, resistance.

Plasmids	Description	Source
pKD46	Recombineering vector, pSC101*ts ori*, P_{BAD}, λ Red system (*gam*, *exo*, *bet*); Ap^R	[8]
pECmulox	Knockout template vector, *lox66-cat-lox77*; Ap^R, Cm^R	[8]
pEcCas	*repA101*(Ts) *kan* P_{cas}-*cas9* P_{araB}-*Red lacI*^{q} P_{rhaB}-sgRNA-*pMB1*, *sacB*; Km^R	[9]
pTargetF	pMB1 *aadA* sgRNA-*pMB1*; Sm^R	[10]
pCDF-hemAL	pCDFDuet-1 derivative, *E. coli hemA*^{fbr} and *hemL*; Sm^R	[8]
pTnsABC	pCOLADuet-1 harboring TnsA, TnsB and TnsC from *V. cholerae* strain HE-45; Km^R	[11]
pTetQCas-8+IS186	pCDFDuet-1 harboring TniQ, Cas8, Cas7 and Cas6 from *V. cholerae* strain HE-45; crRNA was replaced with CRISPR array8+IS186; Sm^R	[11]
pRE57I-GNAc	pUC57mini harboring RE-GlcNAc-LE; Ap^R	[11]
pCutamp	The plasmid harboring SpCas9, rhamnose induction system, SacB and sgRNA targeting the Amp promoter; Am^R	[11]
pRSFDuet-1	Expression vector, RSF1030 *ori*, double T7 *lac* promoters; Km^R	Novagen
pETDuet-1	Expression vector, ColE1 *ori*, double T7 *lac* promoters; Ap^R	Novagen
pCDFDuet-1	Expression vector, CloDF13 *ori*, double T7 *lac* promoters; Sm^R	Novagen
pACYCDuet-1	Expression vector, p15A *ori*, double T7 *lac* promoters; Cm^R	Novagen
pRSF-BM3$_{mut}$	pRSFDuet-1derivative, *Bacillus megaterium* BM3 (A82F/A328F); Km^R	This study
pET-BM3$_{mut}$	pETDuet-1derivative, *Bacillus megaterium* BM3 (A82F/A328F); Ap^R	This study
pRSF-105D7-CAB	pRSFDuet-1derivative, *Streptomyces avermitilis* CYP105D7, *Pseudomonas putida camA* and *camB*; Km^R	This study
pRSF-sca-2$_{mut}$-CAB	pRSFDuet-1derivative, *Streptomyces carbophilus sca-2*$_{mut}$, *Pseudomonas putida camA and camB*; Km^R	This study
pET-sca-2$_{mut}$-CAB	pETDuet-1derivative, *Streptomyces carbophilus sca-2*$_{mut}$, *Pseudomonas putida camA and camB*; Ap^R	This study
pCDF-T7-ChuA	pCDFDuet-1 derivative, *E. coli* Nissle 1917 *chuA*; Sm^R	This study
pCDF-T7-PhuR	pCDFDuet-1 derivative, *Pseudomonas aeruginosa*	This study

	phuR; Sm^R	
pCDF-P_{J23100}-ChuA	pCDF-T7-ChuA derivative, P_{J23100} promoter; Sm^R	This study
pCDF-P_{J23116}-ChuA	pCDF-T7-ChuA derivative, P_{J23116} promoter; Sm^R	This study
pCDF-P_{J23117}-ChuA	pCDF-T7-ChuA derivative, P_{J23117} promoter; Sm^R	This study
pCDF-P_{J23100}-PhuR	pCDF-T7-PhuR derivative, P_{J23100} promoter; Sm^R	This study
pCDF-P_{J23116}-PhuR	pCDF-T7-PhuR derivative, P_{J23116} promoter; Sm^R	This study
pCDF-P_{J23117}-PhuR	pCDF-T7-PhuR derivative, P_{J23117} promoter; Sm^R	This study
HS1	pACYCDuet-1 derivative, *mKATE2*, *eGFP*, Cyt b_{562}; Cm^R	This study
HS1-M7A	HS1 derivative, Cyt $b_{562\ M7A}$; Cm^R	This study
HS1-M7A/H102A	HS1 derivative, Cyt $b_{562\ M7A/H102A}$; Cm^R	This study
pACYC-hemAL	pACYCDuet-1 derivative, *E. coli hemA^fbr^* and *hemL*; Cm^R	This study
pET-hemBDCH	pETDuet-1 derivative, *E. coli hemH*, *hemB*, *hemD*, *hemC*; Ap^R	This study
pACYC-hemAL-Scaf123	pACYCDuet-1 derivative, *E. coli* *hemA*^{fbr} and *hemL*, *scaffold1-scaffold2-scaffold3*; Cm^R	This study
pACYC-hemAL-Scaf132	pACYCDuet-1 derivative, *E. coli* *hemA*^{fbr} and *hemL*, *scaffold1-scaffold3-scaffold2*; Cm^R	This study
pACYC-hemAL-Scaf213	pACYCDuet-1 derivative, *E. coli* *hemA*^{fbr} and *hemL*, *scaffold2-scaffold1-scaffold3*; Cm^R	This study
pACYC-hemAL-Scaf231	pACYCDuet-1 derivative, *E. coli* *hemA*^{fbr} and *hemL*, *scaffold2-scaffold3-scaffold1*; Cm^R	This study
pACYC-hemAL-Scaf312	pACYCDuet-1 derivative, *E. coli* *hemA*^{fbr} and *hemL*, *scaffold3-scaffold1-scaffold2*; Cm^R	This study
pACYC-hemAL-Scaf321	pACYCDuet-1 derivative, *E. coli* *hemA*^{fbr} and *hemL*, *scaffold3-scaffold2-scaffold1*; Cm^R	This study
pACYC-hemAL-Scaf1132	pACYCDuet-1 derivative, *E. coli* *hemA*^{fbr} and *hemL*, *scaffold1-scaffold1-scaffold3-scaffold2*; Cm^R	This study
pACYC-hemAL-Scaf1332	pACYCDuet-1 derivative, *E. coli* *hemA*^{fbr} and *hemL*, *scaffold1-scaffold3-scaffold3-scaffold2*; Cm^R	This study
pACYC-hemAL-Scaf1322	pACYCDuet-1 derivative, *E. coli* *hemA*^{fbr} and *hemL*, *scaffold1-scaffold3-scaffold2-scaffold2*; Cm^R	This study
pET-ADB-hemBDC-hemH	pETDuet-1 derivative, *E. coli ADB1-hemB*, *ADB3-hemD*, *ADB2-hemC*, *hemH*; Ap^R	This study
pRE57I-ADB-BDC-hemH	pRE57I-GNAc derivative, the GlcNAc cassette was replaced with *pT7-hemH-pT7-ADB1-hemB-ADB3-hemD-ADB2-hemC*	This study

	cassette; Ap^{R}	
pACYC-R1	pACYCDuet-1 derivative, *Lactococcus lactis hrtR*, $hrtO_{L}$, *eGFP*; Cm^{R}	This study
pACYC-R2	pACYCDuet-1 derivative, *Enterococcus faecalis fhtR*, $hrtO_{E}$, *eGFP*; Cm^{R}	This study
pACYC-R3	pACYCDuet-1 derivative, *Staphylococcus aureus* $hssR_{S}$, $hssS_{S}$, $hrtO_{S}$, *eGFP*; Cm^{R}	This study
pACYC-R4	pACYCDuet-1 derivative, *Bacillus anthracis* $hssR_{B}$, $hssS_{B}$, $hrtO_{B}$, *eGFP*; Cm^{R}	This study
pACYC-R1/H149 R	pACYC-R1 derivative, HrtR$_{H149K}$; Cm^{R}	This study
pACYC-R1/H149 K	pACYC-R1 derivative, HrtR$_{H149I}$; Cm^{R}	This study
pACYC-R1/H149 I	pACYC-R1 derivative, HrtR$_{H149I}$; Cm^{R}	This study
pACYC-R1/H149 F	pACYC-R1 derivative, HrtR$_{H149F}$; Cm^{R}	This study
pACYC-R1H149 L	pACYC-R1 derivative, HrtR$_{H149L}$; Cm^{R}	This study
pACYC-R1/H149 W	pACYC-R1 derivative, HrtR$_{H149W}$; Cm^{R}	This study
pACYC-R1/H149 A	pACYC-R1 derivative, HrtR$_{H149A}$; Cm^{R}	This study
pACYC-R1/H149 M	pACYC-R1 derivative, HrtR$_{H149M}$; Cm^{R}	This study
pACYC-R1/H149 P	pACYC-R1 derivative, HrtR$_{H149P}$; Cm^{R}	This study
pACYC-R1/H149 C	pACYC-R1 derivative, HrtR$_{H149C}$; Cm^{R}	This study
pACYC-R1/H149 N	pACYC-R1 derivative, HrtR$_{H149N}$; Cm^{R}	This study
pACYC-R1/H149 V	pACYC-R1 derivative, HrtR$_{H149V}$; Cm^{R}	This study
pACYC-R1/H149 G	pACYC-R1 derivative, HrtR$_{H149G}$; Cm^{R}	This study
pACYC-R1/H149 Q	pACYC-R1 derivative, HrtR$_{H149Q}$; Cm^{R}	This study
pACYC-R1/H149 Y	pACYC-R1 derivative, HrtR$_{H149Y}$; Cm^{R}	This study
pACYC-R1/H149 E	pACYC-R1 derivative, HrtR$_{H149E}$; Cm^{R}	This study
pACYC-R1/H149 T	pACYC-R1 derivative, HrtR$_{H149T}$; Cm^{R}	This study
pACYC-R1/H149	pACYC-R1 derivative, HrtR$_{H149S}$; Cm^{R}	This study

S		
pACYC-R1/H149D	pACYC-R1 derivative, $HrtR_{H149D}$; Cm^R	This study
pACYC-R1/H149I	pACYC-R1 derivative, $HrtR_{H149I}$; Cm^R	This study
pACYC-R1/H149F	pACYC-R1 derivative, $HrtR_{H149F}$; Cm^R	This study
pACYC-R1H149L	pACYC-R1 derivative, $HrtR_{H149L}$; Cm^R	This study
pACYC-R1/H149W	pACYC-R1 derivative, $HrtR_{H149W}$; Cm^R	This study
pACYC-R1/H149A	pACYC-R1 derivative, $HrtR_{H149A}$; Cm^R	This study
pACYC-R1/H149M	pACYC-R1 derivative, $HrtR_{H149M}$; Cm^R	This study
pACYC-R1/H72R	pACYC-R1 derivative, $HrtR_{H72R}$; Cm^R	This study
pACYC-R1/H72K	pACYC-R1 derivative, $HrtR_{H72K}$; Cm^R	This study
pACYC-R1/H72I	pACYC-R1 derivative, $HrtR_{H72I}$; Cm^R	This study
pACYC-R1/H72F	pACYC-R1 derivative, $HrtR_{H72F}$; Cm^R	This study
pACYC-R1/H72L	pACYC-R1 derivative, $HrtR_{H72L}$; Cm^R	This study
pACYC-R1/H72W	pACYC-R1 derivative, $HrtR_{H72W}$; Cm^R	This study
pACYC-R1/H72A	pACYC-R1 derivative, $HrtR_{H72A}$; Cm^R	This study
pACYC-R1/H72M	pACYC-R1 derivative, $HrtR_{H72M}$; Cm^R	This study
pACYC-R1/H72P	pACYC-R1 derivative, $HrtR_{H72P}$; Cm^R	This study
pACYC-R1/H72C	pACYC-R1 derivative, $HrtR_{H72C}$; Cm^R	This study
pACYC-R1/H72N	pACYC-R1 derivative, $HrtR_{H72N}$; Cm^R	This study
pACYC-R1/H72V	pACYC-R1 derivative, $HrtR_{H72V}$; Cm^R	This study
pACYC-R1/H72G	pACYC-R1 derivative, $HrtR_{H72G}$; Cm^R	This study
pACYC-R1/H72Q	pACYC-R1 derivative, $HrtR_{H72Q}$; Cm^R	This study
pACYC-R1/H72Y	pACYC-R1 derivative, $HrtR_{H72Y}$; Cm^R	This study
pACYC-R1/H72E	pACYC-R1 derivative, $HrtR_{H72E}$; Cm^R	This study
pACYC-R1/H72T	pACYC-R1 derivative, $HrtR_{H72T}$; Cm^R	This study
pACYC-R1/H72S	pACYC-R1 derivative, $HrtR_{H72S}$; Cm^R	This study
pACYC-R1/H72D	pACYC-R1 derivative, $HrtR_{H72D}$; Cm^R	This study
pACYC-R1/H72R	pACYC-R1 derivative, $HrtR_{H72R}$; Cm^R	This study
pACYC-R1/H72K	pACYC-R1 derivative, $HrtR_{H72K}$; Cm^R	This study
pACYC-R1/H72I	pACYC-R1 derivative, $HrtR_{H72I}$; Cm^R	This study
pACYC-R1/H72F	pACYC-R1 derivative, $HrtR_{H72F}$; Cm^R	This study
pET-T7-EGFP	pETDuet-1 derivative, *eGFP*; Ap^R	This study
pET-P_{J23101}-EGFP	pET-EGFP derivative, P_{J23101} promoter; Ap^R	This study

pACYC-sRNA$_{EGFP}$-R1	pACYC-R1 derivative, *eGFP* was replaced with *sRNA-eGFP-micC*; Cm^R	This study
pACYC-sRNA$_{EGFP}$-R2	pACYC-sRNA$_{EGFP}$-R1 derivative, HrtR$_{H149K}$; Cm^R	This study
pACYC-sRNA$_{EGFP}$-R3	pACYC-sRNA$_{EGFP}$-R1 derivative, HrtR$_{H149I}$; Cm^R	This study
pACYC-sRNA$_{EGFP}$-R4	pACYC-sRNA$_{EGFP}$-R1 derivative, HrtR$_{H149P}$; Cm^R	This study
pACYC-sRNA$_{EGFP}$-R5	pACYC-sRNA$_{EGFP}$-R1 derivative, HrtR$_{H149G}$; Cm^R	This study
pACYC-sRNA$_{HemB}$-HrtR	pACYC-R1 derivative, *eGFP* was replaced with *sRNA-HemB-micC*; Cm^R	This study
pACYC-sRNA$_{HemB}$-HrtR$_{H149K}$	pACYC-sRNA$_{HemB}$-HrtR derivative, HrtR$_{H149K}$; Cm^R	This study

Supplementary Table 3. The primers used for the constructed of plasmids

Primers	Sequence (5'-3') [a]
ChuA-F	GTATAAGAAGGAGATATACATATGTCACGTCCGCAATTTACCTCGT
ChuA-R	AGCGGTTTCTTTACCAGACTCGAGTTACCATTGATAACTCACGAAAATTTTTCCGTTACG
PhuR-F	GTATAAGAAGGAGATATACATATGCCGCTCTCCCCGCCC
PhuR-R	GTTTCTTTACCAGACTCGAGTTAGATGTCCCAGACCAGGTTGACCGCG
P$_{J23100}$-ChuA-F	TAGGTACAGTGCTAGCCCTGTAGAAATAATTTTGTTTAACTTTAATAAGGAGATATACCATGGGCTCACGTCCGCAATTTACCTCGT
P$_{J23100}$-ChuA-R	GGGCTAGCACTGTACCTAGGACTGAGCTAGCCGTCAAATTTCCTAATGCAGGAGTCGCATAAGG
P$_{J23116}$-ChuA-F	TAGGGACTATGCTAGCCCTGTAGAAATAATTTTGTTTAACTTTAATAAGGAGATATACCATGGGCTCACGTCCGCAATTTACCTCGT
P$_{J23116}$-ChuA-R	GGCTAGCATAGTCCCTAGGACTGAGCTAGCTGTCAAATTTCCTAATGCAGGAGTCGCATAAGG
P$_{J23117}$-ChuA-F	TAGGGATTGTGCTAGCCCTGTAGAAATAATTTTGTTTAACTTTAATAAGGAGATATACCATGGGCTCACGTCCGCAATTTACCTCGT
P$_{J23117}$-ChuA-R	GGGCTAGCACAATCCCTAGGACTGAGCTAGCTGTCAAATTTCCTAATGCAGGAGTCGCATAAGG
P$_{J23100}$-PhuR-F	TAGGTACAGTGCTAGCCCTGTAGAAATAATTTTGTTTAACTTTAATAAGGAGATATACCAT
P$_{J23100}$-PhuR-R	GGGCTAGCACTGTACCTAGGACTGAGCTAGCCGTCAAATTTCCTAATGCAGGAGTCGCATAAGG
P$_{J23116}$-PhuR-F	TAGGGACTATGCTAGCCCTGTAGAAATAATTTTGTTTAACTTTAATAAGGAGATATACCAT

P_{J23116}-PhuR-R	GGCTAGCATAGTCCCTAGGACTGAGCTAGCTGTCAAATTTCCTAATGCAGGAGTCGCATAAGG
P_{J23117}-PhuR-F	TAGGGATTGTGCTAGCCCTGTAGAAATAATTTTGTTTAACTTTAATAAGGAGATATACCAT
P_{J23117}-PhuR-R	GGGCTAGCACAATCCCTAGGACTGAGCTAGCTGTCAAATTTCCTAATGCAGGAGTCGCATAAGG
mKATE2-F	AATAAGGAGATATACCATGGGCCACATGGTTTCTGAACTGATCAAAGAAAACATGCACA
mKATE2-R	TTACCCATGCTGCCCCCACGGTGACCCAGTTTAGACGGCA
EGFP-mKATE2-F	TCACCGTGGGGGCAGCATGGGTAAGGGAGAAGAACTTTTCACTG
EGFP-mKATE2-R	CCGAGCTCGAATTCGGATCCTTATTTGTATAGTTCATCCATGCCATGTGTAATCCC
Cyt b_{562}-F	GTGAAGGTGATGCAACATACGGTGGCTCTGCTGATCTTGAAGACAATATGGAAACCCT
Cyt b_{562}-R	TTTAAGGGTAAGTTTTCCACCACGATACTTCTGGTGATAGGCGTTGC
HS1-F	GGTGGAAAACTTACCCTTAAATTTATTTGCACTACT
HS1-R	GTATGTTGCATCACCTTCACCCTCT
HS1-M7A-F	TTGAAGACAATGCGGAAACCCTCAACGACAATTTAAAAGTGATCGA
HS1-M7A-R	AGGGTTTCCGCATTGTCTTCAAGATCAGCAGAGCCAC
HS1-M7A-H102A-F	ACGCCTATGCGCAGAAGTATCGTGGTGGAAAACTTACCCT
HS1-M7A-H102A-R	ATACTTCTGCGCATAGGCGTTGCGGGTCGT
QC-HemA-F	ATGCAAGCAGACTAACCCTATCAACGTTGGTATTATTTCCCGCAGACATGGACACTATAGAACGCGGCCG
QC-HemA-R	ACCCTGTAAAAAAAGAAAATGATGTACTGCTACTCCAGCCCGAGGCTGTCCCGCATAGGCCACTAGTGGA
HemAL-F	CTTTAATAAGGAGATATACCATGGGTACCAAGAAGCTTTTAGCACT
HemAL-R	CTTAAGCATTATGCGGCCGC
HemH-F	GAAGGAGATATACCATGGGCCGTCAGACTAAAACCGGTATCCTGC
HemH-R	GCCGAGCTCGAATTCGGATCCTTAGCGATACGCGGCAACAAGAT
HemB-F1	AGTATAAGAAGGAGATATACATATGACAGACTTAATCCAACGCCCTCGT
HemB-F2	ACCCGTCAGAGCAATATTGGTGGTGGTGGTTCTATGACAGACTTAATCCAACGCCCTCGT
HemB-F3	ATGCGTAGCAATCGTCGTGATCATACCGTGAGCACCCGTCAGAGCAATATTGG
HemB-F	AGTATAAGAAGGAGATATACATATGCGTAGCAATCGTCGTGAT

	CAT
HemB-R	CCTCCTTTTATTTAACGCAGAATCTTCTTCTCAGCCAAATCCAT
HemD-F1	GGCTGAGAAGAAGATTCTGCGTTAAATAAAAGGAGGAAAATA TATGAGTATCCTGGTCACCCGCC
HemD-F2	CAGCCGTCGTAGCCATCGTCGTAGCCACCGTCAGACCCATCAG GGTGGTGGTGGTTCTATGAGTATCCTGGTCACCCGCC
HemD-F3	GGCTGAGAAGAAGATTCTGCGTTAAATAAAAGGAGGAAAATA TATGCAGAGCAGCCGTCGTAGCCATCGT
HemD-R	CCTCCTTTTATTTATTGTAATGCCCGTAAAAGCGCATCGT
HemC-F1	CGCTTTTACGGGCATTACAATAAATAAAAGGAGGAAAATATAT GTTAGACAATGTTTTAAGAATTGCCACACGC
HemC-F2	TAGCCATCGTCGTAGCAATCGCTGCAGCAATCGTGGTGGTGGT GGTTCTATGTTAGACAATGTTTTAAGAATTGCCACACGC
HemC-F3	CGCTTTTACGGGCATTACAATAAATAAAAGGAGGAAAATATAT GGTGAGCAGCCGTCGTAGCCATCGTCGTAGCAATCGCT
HemC-R	GTTTCTTTACCAGACTCGAGTCATGCCGGAGCGTCTCCGTTAT
Scaffold123-F	CATAAGACGAGGGGGTGTATTCACGGAAGGGGGGGTACACTC GAGTCTGGTAAAGAAACCGCT
Scaffold123-R	AATACACCCCCTCGTCTTATGATTCTCCCTAGCTTGTAAGATCT GCCATATGTATATCTCCTTCTTATACT
Scaffold132-F	CATAAGAAGGGGGGGTATATTCACGGACGAGGGGGTGCACTC GAGTCTGGTAAAGAAACCGCT
Scaffold132-R	AATATACCCCCCCTTCTTATGATTCTCCCTAGCTTGTAAGATCT GCCATATGTATATCTCCTTCTTATACT
Scaffold213-F	CATAACAAGCTAGGGAGTATTCACGGAAGGGGGGGTACACTC GAGTCTGGTAAAGAAACCGCT
Scaffold213-R	AATACTCCCTAGCTTGTTATGATTCACCCCCTCGTCTAAGATCT GCCATATGTATATCTCCTTCTTATACT
Scaffold231-F	CATAAGAAGGGGGGGTATATTCACGCAAGCTAGGGAGCACTC GAGTCTGGTAAAGAAACCGCT
Scaffold231-R	AATATACCCCCCCTTCTTATGATTCACCCCCTCGTCTAAGATCT GCCATATGTATATCTCCTTCTTATACT
Scaffold312-F	CATAACAAGCTAGGGAGTATTCACGGACGAGGGGGTGCACTCG AGTCTGGTAAAGAAACCGCT
Scaffold312-R	AATACTCCCTAGCTTGTTATGATTTACCCCCCCTTCTAAGATCT GCCATATGTATATCTCCTTCTTATACT
Scaffold321-F	CATAAGACGAGGGGGTGTATTCACGCAAGCTAGGGAGCACTCG AGTCTGGTAAAGAAACCGCT
Scaffold321-R	AATACACCCCCTCGTCTTATGATTTACCCCCCCTTCTAAGATCT GCCATATGTATATCTCCTTCTTATACT
Scaffold1132-F	AATCATAAGAAGGGGGGGTATATTCACGGACGAGGGGGTGCA CTCGAGTCTGGTAAAGAAACCGCT
Scaffold1132-R	TACCCCCCCTTCTTATGATTCTCCCTAGCTTGTTATGATTCTCCC TAGCTTGTAAGATCTGCCATATGTATATCTCCTTCTTATACT

Scaffold11332-F	CATAAGAAGGGGGGTATATTCACGGAAGGGGGGTAAATCA TAAGACGAGGGGGTGCACTCGAGTCTGGTAAAGAAACCGCT
Scaffold11332-R	AATATACCCCCCTTCTTATGATTCTCCCTAGCTTGTAAGATCT GCCATATGTATATCTCCTTCTTATACT
Scaffold11322-F	CATAAGAAGGGGGGTATATTCACGGACGAGGGGGTGCAATC ATAAGACGAGGGGGTGACTCGAGTCTGGTAAAGAAACCGCT
Scaffold11322-R	AATATACCCCCCTTCTTATGATTCTCCCTAGCTTGTAAGATCT GCCATATGTATATCTCCTTCTTATACT
sgRNA-adhE-F	GGATCAGGTTGATGTCTGGGGTTTTAGAGCTAGAAATAGCAAG TT
sgRNA-adhE-R	CCCAGACATCAACCTGATCCACTAGTATTATACCTAGGACTGA GC
Up-adhE-F	ATTGGCAGCCAATACTTACTGGCAT
Up-adhE-R	AAGGGAGAGCGTCGAGATCCAAAGCGAAAAAATCCGCTTAAT CAGTAGC
Down-adhE-F	GGGGTTTTTTGCTGAAACCTCAGGCATAATGCTCTCCTGATAAT GTTAAACTT
Down-adhE-R	CGCTGTCTGATAACTGGTCATGCTG
Cp-adhE-F	GGATCTCGACGCTCTCCCTT
Cp-adhE-R	CACGTAATCAGTACCCAGAAGTGAGT
sgRNA-cheY-F	CATATTGGGCATGTTCCAGTGTTTTAGAGCTAGAAATAGCAAG TT
sgRNA-cheY-R	ACTGGAACATGCCCAATATGACTAGTATTATACCTAGGACTGA GC
Up-cheY-F	GACATCCATCATCCGCTTAATGACCT
Up-cheY-R	TTTGAGAAACTGGGCATGTGAGGATG
Down-cheY-F	GGGGTTTTTTGCTGAAACCTCAGGCATTTCACACTCCTGATTTA AATACGTATCG
Down-cheY-R	ATATGCCGCCTGGTTTCACC
Cp-cheY-F	TTTGCTGGTATCGACCTGAGGTCC
Cp-cheY-R	ACTGAGGCAATTCGTCACGTACT
IS186-F	CCACTGTAATGATAGGCAATTCAGGT
IS186-1R	ATGAAGCAGCATAAGGCGATGAT
IS186-2R	GAAGCGCAACAGGCATCTG
IS186-3R	ATCGTGAGAATGAGATGTTTGCTGT
IS186-4R	GGATGCTGCAAATCCCACAG
IS186-5R	CAACCTGAGGCTTACGTTCG
Array1-1F	CAACTGGTCACGCTGATCGAC
Array1-1R	CTGTTCAACCTGGTCCAGCC
Array1-2F	GCCATGCTAACTCGTCCAAACAAT
Array1-2R	GCGTATGAAGAGCGGTGAGC
Array1-3F	GTGGAATTACTGGCAGCGAC
Array1-3R	CGGTGGTATATTCCCTTCTGATGA
Array1-4F	CTGCATGTACGTGCCTTCCTC

Array1-4R	GCTACCGAAGGGACTATTCCTCT
Array1-5F	CAGAGATGGAAATTACCCTGCAAGC
Array1-5R	TTGCGTTTGCCGTTCAACAACGG
Array1-6F	GGTGCAAGTATCATCACCACTTCGT
Array1-6R	GTCGATTACGAGACGTTGATGAAGGT
Array1-7F	CTGGTATCGTAAAATGGTTCAACGCT
Array1-7R	ACATACTCGTTGGAAATCAACACAG
Array1-8F	TCTTCCGCATTACCCTGGTGCA
Array1-8R	CGTACACTGAACTCTCTGGCTCACT
ACYC-F	ATGGATGAACTATACAAATAACTCGAGTCTGGTAAAGAAACCGCTG
ACYC-R	TTTTCCACACATTATACGAGCCGATGATTAATTGCCAAGCCTGGAATTTCCTAATGCAGGAGTCGCATAAGG
HrtR-F	GCTCGTATAATGTGTGGAAAAGGAGATATACCATGCCCAAATCTACATATTTTAGTCTATCAGACG
HrtR-R	AGAGCAGAATGCCAGAACCGGCGTTACTTTTTGCTCTCGGTCACGTAGTTC
EGFP-F	ATGGGTAAGGGAGAAGAACTTTTCACT
EGFP-F1	TAATGAATCTGATGACACAGTGTCATCCTCTAGAGATTAAAGAGGAGAAATACTAGATGGGTAAGGGAGAAGAACTTTTCACT
EGFP-F2	CGCCGGTTCTGGCATTCTGCTCTGAGCTGTTGACAATTAATCATCCGGCTCGTATAATGAATCTGATGACACAGTGTCATCCTCT
EGFP-R	TTATTTGTATAGTTCATCCATGCCATGTGTAATCCC
FhtR-F1	ATCATCGGCTCGTATAATGTGTGGAAAAGGAGATATACCATGGAAAAACCCAATAAAAGGAAGGCT
FhtR-F2	TCCAGGCTTGGCAATTAATCATCGGCTCGTATAATGT
FhtR-R1	GCCGGATGATTAATTGTCAACAGCTCAGAGCAGAATGCCAGAACCGGCGTTATTTATGGCTTTTGAGAACGCCGTGC
FhtR-R2	ATTATCAATCGATAAATCAATAATTATTTATCGATTGATAACAGATTCATTATACGAGCCGGATGATTAATTGTCAACAGCT
pACYC-R1-F	TTGATTTATCGATTGATAATCCTCTAGAGATTAAAGAGGAGAAATACTAGATGGGT
pACYC-R1-R	GCCGATGATTAATTGCCAAGCCTGGA
H149R-F	**CGT**AATCTGGTCTATCGCATGTTCAGCGAAAACTGG
H149R-R	ATGCGATAGACCAGATT**ACG**AATAACGCTCTTCAGCACTTGGG
H149K-F	**AAA**AATCTGGTCTATCGCATGTTCAGCGAAAACTGG
H149K-R	ATGCGATAGACCAGATT**TTT**AATAACGCTCTTCAGCACTTGGG
H149I-F	**ATT**AATCTGGTCTATCGCATGTTCAGCGAAAACTGG
H149I-R	ATGCGATAGACCAGATT**AAT**AATAACGCTCTTCAGCACTTGGG
H149F-F	**TTT**AATCTGGTCTATCGCATGTTCAGCGAAAACTGG
H149F-R	ATGCGATAGACCAGATT**AAA**AATAACGCTCTTCAGCACTTGGG
H149L-F	**CTG**AATCTGGTCTATCGCATGTTCAGCGAAAACTGG
H149L-R	ATGCGATAGACCAGATT**CAG**AATAACGCTCTTCAGCACTTGGG
H149W-F	**TGG**AATCTGGTCTATCGCATGTTCAGCGAAAACTGG

H149W-R	ATGCGATAGACCAGATT**CCA**AATAACGCTCTTCAGCACTTGGG
H149A-F	**GCG**AATCTGGTCTATCGCATGTTCAGCGAAAACTGG
H149A-R	ATGCGATAGACCAGATT**CGC**AATAACGCTCTTCAGCACTTGGG
H149M-F	**ATG**AATCTGGTCTATCGCATGTTCAGCGAAAACTGG
H149M-R	ATGCGATAGACCAGATT**CAT**AATAACGCTCTTCAGCACTTGGG
H149P-F	**CCG**AATCTGGTCTATCGCATGTTCAGCGAAAACTGG
H149P-R	ATGCGATAGACCAGATT**CGG**AATAACGCTCTTCAGCACTTGGG
H149C-F	**TGC**AATCTGGTCTATCGCATGTTCAGCGAAAACTGG
H149C-R	ATGCGATAGACCAGATT**GCA**AATAACGCTCTTCAGCACTTGGG
H149N-F	**AAT**AATCTGGTCTATCGCATGTTCAGCGAAAACTGG
H149N-R	ATGCGATAGACCAGATT**ATT**AATAACGCTCTTCAGCACTTGGG
H149V-F	**GTG**AATCTGGTCTATCGCATGTTCAGCGAAAACTGG
H149V-R	ATGCGATAGACCAGATT**CAC**AATAACGCTCTTCAGCACTTGGG
H149G-F	**GGC**AATCTGGTCTATCGCATGTTCAGCGAAAACTGG
H149G-R	ATGCGATAGACCAGATT**GCC**AATAACGCTCTTCAGCACTTGGG
H149Q-F	**CAG**AATCTGGTCTATCGCATGTTCAGCGAAAACTGG
H149Q-R	ATGCGATAGACCAGATT**CTG**AATAACGCTCTTCAGCACTTGGG
H149Y-F	**TAT**AATCTGGTCTATCGCATGTTCAGCGAAAACTGG
H149Y-R	ATGCGATAGACCAGATT**ATA**AATAACGCTCTTCAGCACTTGGG
H149E-F	**GAA**AATCTGGTCTATCGCATGTTCAGCGAAAACTGG
H149E-R	ATGCGATAGACCAGATT**TTC**AATAACGCTCTTCAGCACTTGGG
H149T-F	**ACC**AATCTGGTCTATCGCATGTTCAGCGAAAACTGG
H149T-R	ATGCGATAGACCAGATT**GGT**AATAACGCTCTTCAGCACTTGGG
H149S-F	**AGT**AATCTGGTCTATCGCATGTTCAGCGAAAACTGG
H149S-R	ATGCGATAGACCAGATT**ACT**AATAACGCTCTTCAGCACTTGGG
H149D-F	**GAT**AATCTGGTCTATCGCATGTTCAGCGAAAACTGG
H149D-R	ATGCGATAGACCAGATT**ATC**AATAACGCTCTTCAGCACTTGGG
H72-R	AATTTCAACCGTTTCTTGGGACAGAAT
H72R-F	AAGAAACGGTTGAAATT**CGT**GATCTGTTTTTCAACCTGTTGAA GGAGT
H72K-F	AAGAAACGGTTGAAATT**AAA**GATCTGTTTTTCAACCTGTTGAA GGAGT
H72I-F	AAGAAACGGTTGAAATT**ATT**GATCTGTTTTTCAACCTGTTGAA GGAGT
H72F-F	AAGAAACGGTTGAAATT**TTT**GATCTGTTTTTCAACCTGTTGAAG GAGT
H72L-F	AAGAAACGGTTGAAATT**CTG**GATCTGTTTTTCAACCTGTTGAA GGAGT
H72W-F	AAGAAACGGTTGAAATT**TGG**GATCTGTTTTTCAACCTGTTGAA GGAGT
H72A-F	AAGAAACGGTTGAAATT**GCG**GATCTGTTTTTCAACCTGTTGAA GGAGT
H72M-F	AAGAAACGGTTGAAATT**ATG**GATCTGTTTTTCAACCTGTTGAA GGAGT

H72P-F	AAGAAACGGTTGAAATT**CCG**GATCTGTTTTTCAACCTGTTGAAGGAGT
H72C-F	AAGAAACGGTTGAAATT**TGC**GATCTGTTTTTCAACCTGTTGAAGGAGT
H72N-F	AAGAAACGGTTGAAATT**AAT**GATCTGTTTTTCAACCTGTTGAAGGAGT
H72V-F	AAGAAACGGTTGAAATT**GTG**GATCTGTTTTTCAACCTGTTGAAGGAGT
H72G-F	AAGAAACGGTTGAAATT**GGC**GATCTGTTTTTCAACCTGTTGAAGGAGT
H72Q-F	AAGAAACGGTTGAAATT**CAG**GATCTGTTTTTCAACCTGTTGAAGGAGT
H72Y-F	AAGAAACGGTTGAAATT**TAT**GATCTGTTTTTCAACCTGTTGAAGGAGT
H72E-F	AAGAAACGGTTGAAATT**GAA**GATCTGTTTTTCAACCTGTTGAAGGAGT
H72T-F	AAGAAACGGTTGAAATT**ACC**GATCTGTTTTTCAACCTGTTGAAGGAGT
H72S-F	AAGAAACGGTTGAAATT**AGT**GATCTGTTTTTCAACCTGTTGAAGGAGT
H72D-F	AAGAAACGGTTGAAATT**GAT**GATCTGTTTTTCAACCTGTTGAAGGAGT
T7_GFP-F	GTATAAGAAGGAGATATACATATGGGTAAGGGAGAAGAACTTTTCACTG
T7_GFP-R	CAGCGGTTTCTTTACCAGACTCGAGTTATTTGTATAGTTCATCCATGCCATGTGTAATCC
P_{J23101}_EGFP-F	TAGGTATTATGCTAGCATCTTAGTATATTAGTTAAGTATAAGAAGGAGATATACATATGG
P_{J23101}_EGFP-R	ATGCTAGCATAATACCTAGGACTGAGCTAGCTGTAAAATTTCGATTATGCGGCCGTGTAC
ACYC_HrtR-F1	CTCGAGTCTGGTAAAGAAACCGCTG
ACYC_HrtR-R1	TTTTCAATCTCTAGAGGATGACACTGTGTCAT
micC-F1	ATCCTCTAGAGATTGAAAAGTTCTTCTCCCTTACCCATTTTCTGTTGGGCCATTGCATT
micC-R1	GTTTCTTTACCAGACTCGAGAAAAAAAGCCCGGACGACTGT
ACYC_HrtR-F2	CTCGAGTCTGGTAAAGAAACCGCTG
ACYC_HrtR-R2	GCCCTAATCTCTAGAGGATGACACTGTGTCAT
micC-F2	ATCCTCTAGAGATTAGGGCGTTGGATTAAGTCTGTCATTTTCTGTTGGGCCATTGCATT
micC-R2	GTTTCTTTACCAGACTCGAGAAAAAAAGCCCGGACGACTGT
RT_hemB-F	GGTATTCGTTCCGTGATG
RT_hemB-R	CCGTGAGATGTGTATTCG
RT_EGFP-F	ACTTGTCACTACTCTTACTTATGG
RT_EGFP-R	TCCTTGAAGAAGATGGTCCTC

RT_hcaT-F	GGCATTATGGGAGCAACTAC
RT_hcaT-R	GGCGAGCAGCAATATAACG

[a] Amino acid mutation sites are shown in bold.

Supplementary Table 4. Strains used in the study.

Strains	Description	Source
DH5α	*E. coli* str. K-12 F^{-} *endA1 glnV44 thi-1 recA1 relA1 gyrA96 deoR nupG purB20* φ80d*lacZ*ΔM15 Δ(*lacZYA-argF*) U169 *hsdR17*($r_K^{-}m_K^{+}$) λ^{-}	Invitrogen
BL21(DE3)	*E. coli str*. B F^{-} *ompT gal dcm lon hsdS*$_B$($r_B^{-}m_B^{-}$) λ (DE3 [*lacI lacUV5-T7p07 ind1 sam7 nin5*]) [*malB*$^{+}$]$_{K-12}$(λ^{S})	Novagen
C41(DE3)	Derived from BL21(DE3) by selecting for resistance to OGCP overexpression	Novagen
C43(DE3)	Derived from C41(DE3) by selecting for resistance to Ecb overexpression	Novagen
B21-pRSF-BM3$_{mut}$	BL21(DE3) harboring plasmid pRSF-BM3$_{mut}$; Km^{R}	This study
C41-pRSF-BM3$_{mut}$	C41(DE3) harboring plasmid pRSF-BM3$_{mut}$; Km^{R}	This study
C43-pRSF-BM3$_{mut}$	C43(DE3) harboring plasmid pRSF-BM3$_{mut}$; Km^{R}	This study
B21-pET-BM3$_{mut}$	BL21(DE3) harboring plasmid pET-BM3$_{mut}$; Ap^{R}	This study
C41-pET-BM3$_{mut}$	C41(DE3) harboring plasmid pET-BM3$_{mut}$; Ap^{R}	This study
C43-pET-BM3$_{mut}$	C43(DE3) harboring plasmid pET-BM3$_{mut}$; Ap^{R}	This study
C41-pRSF-105D7	C41(DE3) harboring plasmid pRSF-105D7-CAB; Km^{R}	This study
B21-pRSF-sca-2$_{mut}$	BL21(DE3) harboring plasmid pRSF-sca-2$_{mut}$-CAB; Km^{R}	This study
C41-pRSF-sca-2$_{mut}$	C41(DE3) harboring plasmid pRSF-sca-2$_{mut}$-CAB; Km^{R}	This study
C43-pRSF-sca-2$_{mut}$	C43(DE3) harboring plasmid pRSF-sca-2$_{mut}$-CAB; Km^{R}	This study
B21-pET-sca-2$_{mut}$	BL21(DE3) harboring plasmid pET-sca-2$_{mut}$-CAB; Ap^{R}	This study
C41-pET-sca-2$_{mut}$	C41(DE3) harboring plasmid pET-sca-2$_{mut}$-CAB; Ap^{R}	This study
C43-pET-sca-2$_{mut}$	C43(DE3) harboring plasmid pET-sca-2$_{mut}$-CAB; Ap^{R}	This study
C41ΔhemA	C41(DE3) with deleting the *hemA*	This study
HEME-T1	C41ΔhemA harboring plasmid pCDF-P$_{J23117}$-PhuR; Sm^{R}	This study
HEME-T2	C41ΔhemA harboring plasmid pCDF-P$_{J23116}$-PhuR; Sm^{R}	This study
HEME-T3	C41ΔhemA harboring plasmid pCDF-P$_{J23100}$-PhuR; Sm^{R}	This study
HEME-T4	C41ΔhemA harboring plasmid pCDF-P$_{J23117}$-ChuA; Sm^{R}	This study
HEME-T5	C41ΔhemA harboring plasmid pCDF-P$_{J23116}$-ChuA; Sm^{R}	This study
HEME-T6	C41ΔhemA harboring plasmid pCDF-P$_{J23100}$-ChuA; Sm^{R}	This study
HEME-T7	*P$_{j23117}$-ChuA* integrated into the *adhE* locus of	This study

	C41ΔhemA chromosome	
HEME-T8	C41(DE3) harboring plasmids pCDF-P$_{J23117}$-ChuA, pRSF-BM3$_{mut}$; Km^R, Sm^R	This study
HEME-T9	C41(DE3) harboring plasmids pCDF-T7-ChuA, pRSF-BM3$_{mut}$; Km^R, Sm^R	This study
HEME-T10	C41(DE3) harboring plasmids pCDF-T7-ChuA, pRSF-sca-2$_{mut}$-CAB; Km^R, Sm^R	This study
HEME-T4-HS1	C41ΔhemA harboring plasmids pCDF-P$_{J23117}$-ChuA, HS1; Sm^R, Cm^R	This study
HEME-T4-HS1$_{M7A}$	C41ΔhemA harboring plasmids pCDF-P$_{J23117}$-ChuA, HS1-M7A; Sm^R, Cm^R	This study
HEME-T4-HS1$_{M7A/H102A}$	C41ΔhemA harboring plasmids pCDF-P$_{J23117}$-ChuA, HS1-M7A/H102A; Sm^R, Cm^R	This study
HEME-S1	C41(DE3) harboring plasmids pET-hemBDCH, pACYC-hemAL; Ap^R, Cm^R	This study
HEME-S2	C41(DE3) harboring plasmids pET-ADB-hemBDC-hemH, pACYC-hemAL-Sacf123; Ap^R, Cm^R	This study
HEME-S3	C41(DE3) harboring plasmids pET-ADB-hemBDC-hemH, pACYC-hemAL-Sacf132; Ap^R, Cm^R	This study
HEME-S4	C41(DE3) harboring plasmids pET-ADB-hemBDC-hemH, pACYC-hemAL-Sacf213; Ap^R, Cm^R	This study
HEME-S5	C41(DE3) harboring plasmids pET-ADB-hemBDC-hemH, pACYC-hemAL-Sacf231; Ap^R, Cm^R	This study
HEME-S6	C41(DE3) harboring plasmids pET-ADB-hemBDC-hemH, pACYC-hemAL-Sacf312; Ap^R, Cm^R	This study
HEME-S7	C41(DE3) harboring plasmids pET-ADB-hemBDC-hemH, pACYC-hemAL-Sacf321; Ap^R, Cm^R	This study
HEME-S8	C41(DE3) harboring plasmids pET-ADB-hemBDC-hemH, pACYC-hemAL-Sacf1132; Ap^R, Cm^R	This study
HEME-S9	C41(DE3) harboring plasmids pET-ADB-hemBDC-hemH, pACYC-hemAL-Sacf1132; Ap^R, Cm^R	This study
HEME-S10	C41(DE3) harboring plasmids pET-ADB-hemBDC-hemH, pACYC-hemAL-Sacf1322; Ap^R, Cm^R	This study
HEME-S8-BM3$_{mut}$	C41(DE3) harboring plasmids pET-hemH-ADB-BDC, pACYC-hemAL-Sacf1132, pRSF-BM3$_{mut}$; Ap^R, Cm^R,	This study

	Km^R	
HEME-S11	*hemA*^{fbr}-*hemL-scaffold1-scaffold1-scaffold3-scaffold2* integrated into the *cheY* locus and the fragment of pT7-*hemH*-pT7-*ADB1-hemB-ADB3-hemD-ADB2-hemC* multicopy chromosomal integrated in C41(DE3) (7 copies)	This study
HEME-S12	*hemA*^{fbr}-*hemL-scaffold1-scaffold1-scaffold3-scaffold2* integrated into the *cheY* locus and the fragment of pT7-*hemH*-pT7-*ADB1-hemB-ADB3-hemD-ADB2-hemC* multicopy chromosomal integrated in C41(DE3) (8 copies)	This study
HEME-S13	*hemA*^{fbr}-*hemL-scaffold1-scaffold1-scaffold3-scaffold2* integrated into the *cheY* locus and the fragment of pT7-*hemH*-pT7-*ADB1-hemB-ADB3-hemD-ADB2-hemC* multicopy chromosomal integrated in C41(DE3) (9 copies)	This study
HEME-S14	*hemA*^{fbr}-*hemL-scaffold1-scaffold1-scaffold3-scaffold2* integrated into the *cheY* locus and the fragment of pT7-*hemH*-pT7-*ADB1-hemB-ADB3-hemD-ADB2-hemC* multicopy chromosomal integrated in C41(DE3) (10 copies)	This study
HEME-S15	*hemA*^{fbr}-*hemL-scaffold1-scaffold1-scaffold3-scaffold2* integrated into the *cheY* locus and the fragment of pT7-*hemH*-pT7-*ADB1-hemB-ADB3-hemD-ADB2-hemC* multicopy chromosomal integrated in C41(DE3) (11 copies)	This study
HEME-S11-BM3$_{mut}$	HEME-S11 harboring plasmid pRSF-BM3$_{mut}$; Km^R	This study
HEME-S12-BM3$_{mut}$	HEME-S12 harboring plasmid pRSF-BM3$_{mut}$; Km^R	This study
HEME-S13-BM3$_{mut}$	HEME-S13 harboring plasmid pRSF-BM3$_{mut}$; Km^R	This study
HEME-S14-BM3$_{mut}$	HEME-S14 harboring plasmid pRSF-BM3$_{mut}$; Km^R	This study
HEME-S15-BM3$_{mut}$	HEME-S15 harboring plasmid pRSF-BM3$_{mut}$; Km^R	This study
HEME-R1	C41(DE3) harboring plasmids pCDF-P$_{J23117}$-ChuA, pACYC-R1; Sm^R, Cm^R	This study
HEME-R2	C41(DE3) harboring plasmids pCDF-P$_{J23117}$-ChuA, pACYC-R2; Sm^R, Cm^R	This study
HEME-R3	C41(DE3) harboring plasmids pCDF-P$_{J23117}$-ChuA, pACYC-R3; Sm^R, Cm^R	This study
HEME-R4	C41(DE3) harboring plasmids pCDF-P$_{J23117}$-ChuA, pACYC-R4; Sm^R, Cm^R	This study

HEME-R1/H149R	C41(DE3) harboring plasmids pCDF-P_{J23117}-ChuA, pACYC-R1/H149R; Sm^R, Cm^R	This study
HEME-R1/H149 K	C41(DE3) harboring plasmids pCDF-P_{J23117}-ChuA, pACYC-R1/H149K; Sm^R, Cm^R	This study
HEME-R1/H149I	C41(DE3) harboring plasmids pCDF-P_{J23117}-ChuA, pACYC-R1/H149I; Sm^R, Cm^R	This study
HEME-R1/H149F	C41(DE3) harboring plasmids pCDF-P_{J23117}-ChuA, pACYC-R1/H149F; Sm^R, Cm^R	This study
HEME-R1/H149L	C41(DE3) harboring plasmids pCDF-P_{J23117}-ChuA, pACYC-R1/H149L; Sm^R, Cm^R	This study
HEME-R1/H149 W	C41(DE3) harboring plasmids pCDF-P_{J23117}-ChuA, pACYC-R1/H149W; Sm^R, Cm^R	This study
HEME-R1/H149 A	C41(DE3) harboring plasmids pCDF-P_{J23117}-ChuA, pACYC-R1/H149A; Sm^R, Cm^R	This study
HEME-R1/H149 M	C41(DE3) harboring plasmids pCDF-P_{J23117}-ChuA, pACYC-R1/H149M; Sm^R, Cm^R	This study
HEME-R1/H149P	C41(DE3) harboring plasmids pCDF-P_{J23117}-ChuA, pACYC-R1/H149P; Sm^R, Cm^R	This study
HEME-R1/H149C	C41(DE3) harboring plasmids pCDF-P_{J23117}-ChuA, pACYC-R1/H149C; Sm^R, Cm^R	This study
HEME-R1/H149 N	C41(DE3) harboring plasmids pCDF-P_{J23117}-ChuA, pACYC-R1/H149N; Sm^R, Cm^R	This study
HEME-R1/H149 V	C41(DE3) harboring plasmids pCDF-P_{J23117}-ChuA, pACYC-R1/H149V; Sm^R, Cm^R	This study
HEME-R1/H149 G	C41(DE3) harboring plasmids pCDF-P_{J23117}-ChuA, pACYC-R1/H149G; Sm^R, Cm^R	This study
HEME-R1/H149 Q	C41(DE3) harboring plasmids pCDF-P_{J23117}-ChuA, pACYC-R1/H149Q; Sm^R, Cm^R	This study
HEME-R1/H149 Y	C41(DE3) harboring plasmids pCDF-P_{J23117}-ChuA, pACYC-R1/H149Y; Sm^R, Cm^R	This study
HEME-R1/H149E	C41(DE3) harboring plasmids pCDF-P_{J23117}-ChuA, pACYC-R1/H149E; Sm^R, Cm^R	This study
HEME-R1/H149T	C41(DE3) harboring plasmids pCDF-P_{J23117}-ChuA, pACYC-R1/H149T; Sm^R, Cm^R	This study
HEME-R1/H149S	C41(DE3) harboring plasmids pCDF-P_{J23117}-ChuA, pACYC-R1/H149S; Sm^R, Cm^R	This study
HEME-R1/H149 D	C41(DE3) harboring plasmids pCDF-P_{J23117}-ChuA, pACYC-R1/H149D; Sm^R, Cm^R	This study
HEME-R1/H72R	C41(DE3) harboring plasmids pCDF-P_{J23117}-ChuA, pACYC-R1/H72R; Sm^R, Cm^R	This study
HEME-R1/H72K	C41(DE3) harboring plasmids pCDF-P_{J23117}-ChuA, pACYC-R1/H72K; Sm^R, Cm^R	This study
HEME-R1/H72I	C41(DE3) harboring plasmids pCDF-P_{J23117}-ChuA, pACYC-R1/H72I; Sm^R, Cm^R	This study

HEME-R1/H72F	C41(DE3) harboring plasmids pCDF-P_{J23117}-ChuA, pACYC-R1/H72F; Sm^R, Cm^R	This study
HEME-R1/H72L	C41(DE3) harboring plasmids pCDF-P_{J23117}-ChuA, pACYC-R1/H72L; Sm^R, Cm^R	This study
HEME-R1/H72W	C41(DE3) harboring plasmids pCDF-P_{J23117}-ChuA, pACYC-R1/H72W; Sm^R, Cm^R	This study
HEME-R1/H72A	C41(DE3) harboring plasmids pCDF-P_{J23117}-ChuA, pACYC-R1/H72A; Sm^R, Cm^R	This study
HEME-R1/H72M	C41(DE3) harboring plasmids pCDF-P_{J23117}-ChuA, pACYC-R1/H72M; Sm^R, Cm^R	This study
HEME-R1/H72P	C41(DE3) harboring plasmids pCDF-P_{J23117}-ChuA, pACYC-R1/H72P; Sm^R, Cm^R	This study
HEME-R1/H72C	C41(DE3) harboring plasmids pCDF-P_{J23117}-ChuA, pACYC-R1/H72C; Sm^R, Cm^R	This study
HEME-R1H72N	C41(DE3) harboring plasmids pCDF-P_{J23117}-ChuA, pACYC-R1/H72N; Sm^R, Cm^R	This study
HEME-R1/H72V	C41(DE3) harboring plasmids pCDF-P_{J23117}-ChuA, pACYC-R1/H72V; Sm^R, Cm^R	This study
HEME-R1/H72G	C41(DE3) harboring plasmids pCDF-P_{J23117}-ChuA, pACYC-R1/H72G; Sm^R, Cm^R	This study
HEME-R1/H72Q	C41(DE3) harboring plasmids pCDF-P_{J23117}-ChuA, pACYC-R1/H72Q; Sm^R, Cm^R	This study
HEME-R1/H72Y	C41(DE3) harboring plasmids pCDF-P_{J23117}-ChuA, pACYC-R1/H72Y; Sm^R, Cm^R	This study
HEME-R1/H72E	C41(DE3) harboring plasmids pCDF-P_{J23117}-ChuA, pACYC-R1/H72E; Sm^R, Cm^R	This study
HEME-R1/H72T	C41(DE3) harboring plasmids pCDF-P_{J23117}-ChuA, pACYC-R1/H72T; Sm^R, Cm^R	This study
HEME-R1/H72S	C41(DE3) harboring plasmids pCDF-P_{J23117}-ChuA, pACYC-R1/H72S; Sm^R, Cm^R	This study
HEME-R1/H72D	C41(DE3) harboring plasmids pCDF-P_{J23117}-ChuA, pACYC-R1/H72D; Sm^R, Cm^R	This study
HEME-R5	C41(DE3) harboring plasmids pACYC-$sRNA_{EGFP}$-R1, pET-P_{J23101}-EGFP; Ap^R, Cm^R	This study
HEME-R6	C41(DE3) harboring plasmids pACYC-$sRNA_{EGFP}$-R2, pET-P_{J23101}-EGFP; Ap^R, Cm^R	This study
HEME-R7	C41(DE3) harboring plasmids pACYC-$sRNA_{EGFP}$-R3, pET-P_{J23101}-EGFP; Ap^R, Cm^R	This study
HEME-R8	C41(DE3) harboring plasmids pACYC-$sRNA_{EGFP}$-R4, pET-P_{J23101}-EGFP; Ap^R, Cm^R	This study
HEME-R9	C41(DE3) harboring plasmids pACYC-$sRNA_{EGFP}$-R5, pET-P_{J23101}-EGFP; Ap^R, Cm^R	This study
C41(DE3)-P_{J23101}-EGFP	C41(DE3) harboring plasmid pET-P_{J23101}-EGFP; Ap^R	This study

HEME-R10	HEME-S13 harboring plasmid pACYC-sRNA$_{HemB}$-HrtR; Cm^{R}	This study
HEME-R11	HEME-S13 harboring plasmid pACYC-sRNA$_{HemB}$-HrtR$_{H149K}$; Cm^{R}	This study
HEME-R10-BM3$_{mut}$	HEME-S13 harboring plasmids pACYC-sRNA$_{HemB}$-HrtR, pRSF-BM3$_{mut}$; Km^{R}, Cm^{R}	This study
HEME-R11-BM3$_{mut}$	HEME-S13 harboring plasmids pACYC-sRNA$_{HemB}$-HrtR$_{H149K}$, pRSF-BM3$_{mut}$; Km^{R}, Cm^{R}	This study
HEME-R11-Sca-2$_{mut}$	HEME-S13 harboring plasmids pACYC-sRNA$_{HemB}$-HrtR$_{H149K}$, pRSF-Sca-2$_{mut}$-CAB; Km^{R}, Cm^{R}	This study
HEME-R11-105D7	HEME-S13 harboring plasmids pACYC-sRNA$_{HemB}$-HrtR$_{H149K}$, pRSF-105D7-CAB; Km^{R}, Cm^{R}	This study

Supplementary Notes

Heterologous gene sequences used in this study.

1. Gene sequence of BM3 (A82F/A328F)

ATGACCATCAAAGAAATGCCGCAGCCGAAGACCTTTGGCGAGCTGAAAAA
CCTGCCGCTGCTGAACACCGACAAGCCGGTGCAAGCGCTGATGAAAATCG
CGGATGAACTGGGCGAGATTTTCAAGTTTGAGGCGCCGGGCCGTGTTACCC
GTTACCTGAGCAGCCAGCGTCTGATCAAAGAGGCGTGCGACGAAAGCCGT
TTCGATAAGAACCTGAGCCAAGCGCTGAAATTTGTGCGTGACTTCTTTGGT
GATGGCCTGTTCACCAGCTGGACCCACGAAAAGAACTGGAAGAAAGCGC
ACAACATCCTGCTGCCGAGCTTCAGCCAGCAAGCGATGAAGGGTTATCACG
CGATGATGGTGGACATTGCGGTGCAGCTGGTTCAAAAATGGGAGCGTCTG
AACGCGGATGAACACATCGAGGTTCCGGAAGACATGACCCGTCTGACCCT
GGATACCATTGGCCTGTGCGGTTTTAACTACCGTTTCAACAGCTTTTATCGT
GACCAGCCGCACCCGTTCATCACCAGCATGGTTCGTGCGCTGGATGAAGCG
ATGAACAAGCTGCAACGTGCGAACCCGGACGATCCGGCGTACGACGAAAA
CAAGCGTCAGTTTCAAGAGGATATTAAAGTGATGAACGACCTGGTTGATAA
GATCATTGCGGACCGTAAAGCGAGCGGCGAGCAGAGCGACGATCTGCTGA
CCCACATGCTGAACGGTAAAGATCCGGAGACCGGCGAACCGCTGGACGAT
GAAAACATCCGTTACCAAATCATTACCTTCCTGATTGCGGGTCATGAGACC
ACCAGCGGTCTGCTGAGCTTCGCGCTGTATTTTCTGGTGAAGAACCCGCAC
GTGCTGCAAAAGGCGGCGGAGGAAGCGGCGCGTGTGCTGGTTGACCCGG
TGCCGAGCTACAAGCAGGTTAAACAACTGAAGTATGTGGGTATGGTTCTGA
ACGAAGCGCTGCGTCTGTGGCCGACCTTCCCGGCGTTTAGCCTGTACGCGA
AAGAGGACACCGTGCTGGGTGGCGAGTATCCGCTGGAAAAAGGTGACGA
GCTGATGGTTCTGATCCCGCAGCTGCACCGTGATAAGACCATTTGGGGCGA

CGATGTGGAGGAGTTCCGTCCGGAGCGTTTTGAAAACCCGAGCGCGATCC
CGCAGCACGCGTTCAAACCGTTTGGTAACGGCCAACGTGCGTGCATTGGTC
AGCAATTTGCGCTGCACGAAGCGACCCTGGTTCTGGGCATGATGCTGAAGC
ACTTCGACTTTGAGGATCACACCAACTACGAACTGGACATCAAGGAGACC
CTGACCCTGAAACCGGAGGGTTTCGTGGTTAAGGCGAAAAGCAAGAAAAT
CCCGCTGGGTGGCATTCCGAGCCCGAGCACCGAACAGAGCGCGAAGAAA
GTGCGTAAGAAAGCGGAGAACGCGCACAACACCCCGCTGCTGGTTCTGTA
CGGCAGCAACATGGGCACCGCGGAGGGCACCGCGCGTGACCTGGCGGAC
ATCGCGATGAGCAAAGGCTTTGCGCCGCAAGTGGCGACCCTGGACAGCCA
TGCGGGTAACCTGCCGCGTGAGGGTGCGGTGCTGATTGTTACCGCGAGCTA
TAACGGTCACCCGCCGGATAACGCGAAGCAGTTCGTTGACTGGCTGGATCA
AGCGAGCGCGGACGAAGTGAAAGGCGTTCGTTACAGCGTGTTTGGTTGCG
GCGATAAGAACTGGGCGACCACCTATCAGAAAGTTCCGGCGTTCATTGATG
AGACCCTGGCGGCGAAGGGTGCGGAAAACATTGCGGACCGTGGCGAGGC
GGATGCGAGCGACGATTTTGAAGGCACCTACGAGGAATGGCGTGAGCACA
TGTGGAGCGATGTGGCGGCGTATTTCAACCTGGACATCGAGAACAGCGAA
GATAACAAGAGCACCCTGAGCCTGCAATTTGTTGACAGCGCGGCGGATATG
CCGCTGGCGAAGATGCACGGTGCGTTCAGCACCAACGTGGTTGCGAGCAA
AGAGCTGCAACAACCGGGCAGCGCGCGTAGCACCCGTCACCTGGAAATCG
AGCTGCCGAAAGAAGCGAGCTACCAAGAGGGTGACCACCTGGGCGTGATC
CCGCGTAACTATGAAGGTATTGTGAACCGTGTTACCGCGCGTTTTGGCCTG
GATGCGAGCCAGCAAATTCGTCTGGAGGCGGAGGAAGAGAAGCTGGCGC
ACCTGCCGCTGGCGAAAACCGTGAGCGTTGAAGAGCTGCTGCAATACGTG
GAGCTGCAAGACCCGGTTACCCGTACCCAGCTGCGTGCGATGGCGGCGAA
GACCGTGTGCCCGCCGCACAAAGTTGAACTGGAGGCGCTGCTGGAAAAAC
AGGCGTACAAGGAGCAAGTTCTGGCGAAGCGTCTGACCATGCTGGAGCTG
CTGGAAAAGTATCCGGCGTGCGAAATGAAATTCAGCGAGTTTATCGCGCTG
CTGCCGAGCATTCGTCCGCGTTACTATAGCATCAGCAGCAGCCCGCGTGTG
GACGAAAAGCAGGCGAGCATTACCGTTAGCGTGGTTAGCGGTGAAGCGTG
GAGCGGTTACGGCGAGTATAAAGGCATCGCGAGCAACTATCTGGCGGAGCT
GCAAGAGGGTGACACCATCACCTGCTTCATTAGCACCCCGCAAAGCGAATT
TACCCTGCCGAAAGATCCGGAGACCCCGCTGATTATGGTTGGTCCGGGCAC
CGGTGTTGCGCCGTTCCGTGGCTTTGTGCAGGCGCGTAAACAACTGAAGG
AACAGGGTCAAAGCCTGGGCGAGGCGCACCTGTATTTCGGTTGCCGTAGC
CCGCACGAGGACTACCTGTATCAGGAAGAGCTGGAAAACGCGCAAAGCGA
GGGCATCATTACCCTGCACACCGCGTTCAGCCGTATGCCGAACCAGCCGAA
GACCTATGTGCAGCACGTTATGGAACAAGACGGTAAGAAACTGATCGAGCT
GCTGGATCAGGGCGCGCACTTCTACATTTGCGGTGATGGTAGCCAAATGGC
TCCGGCGGTGGAAGCGACCCTGATGAAAAGCTATGCGGACGTGCACCAAG
TTAGCGAGGCGGATGCGCGTCTGTGGCTGCAACAACTGGAAGAGAAAGGT
CGTTACGCGAAGGATGTTTGGGCGGGCCACCACCACCACCACCACTAA

2. **Gene sequence of sca-2 (G52S/T85F/F89I/T119S/P159A/V194N/D269E/T323A/N363Y/E370V)**

ATGACCGAGATGACCGAAAAGGCGACCACCTTCCTGACCAGCCAGGAAGC
TCCGGCGTTTCCGGCGGACCGTACCTGCCCGTACCAACTGCCGACCGCGTA
TAGCCGTCTGCGTGACGAACCGGATGCGCTGCGTCCGGTGACCCTGTACGA
CAGCCGTCGTGCGTGGGTGGTTACCAAGCACGAGGCGGCGCGTCGTCTGC
TGGCGGACCCGCGTCTGAGCAGCGACCGTCTGCACGCGGATTTCCCGGCG
TTTAGCCCGCGTATCAAAGCGTTCCGTCAGGGTAGCCCGGCGTTTATTGGCA
TGGACCCGCCGGAGCATGGCACCCGTCGTCGTATGACCATCAGCGAGTTCA
GCGTTAAGCGTATTAAAGGCATGCGTCCGGACGTGGAGCGTATCGTTCACG
GTTTCATTGATGATATGCTGGCGGCGGGTCCGACCGCGGATCTGGTTAGCC
AATTTGCGCTGCCGGTGGCGAGCATGGTTATCTGCCACATGCTGGGTGTTC
CGTACGCGGACCACGAGTTCTTTCAGGATGCGAGCAAACGTCTGGTGCAA
GCGGTTGACGCGGATAGCGCGAACGCGGCGCGTGACGATTTTGAACGTTAT
CTGGACGGCCTGATTACCAAGCTGGAGAGCGAACCGGGCACCGGTCTGCT
GGGTAAACTGGTGACCCATCAGCTGGCGGATGGTGAAATCGACCGTGCGG
AACTGATTAGCACCGCGCTGCTGCTGCTGGTTGCGGGTCACGAAACCACC
GCGAGCATGACCAGCCTGAGCGTTATCACCCTGCTGGAGCACCCGGAACA
ACATGCGGCGCTGCGTGCGGACCCGAGCCTGGTGCCGGGTGCGGTTGAGG
AACTGCTGCGTGTTCTGGCGATTGCGGATATTGCGGGTGGCCGTATTGCGA
CCGCGGACATCGAGATTGATGGCCAACTGATTCGTGCGGGTGAAGGCGTGA
TCGTTGCGAACAGCATTGCGAACCGTGACAGCAGCGTGTTCGAGAACCCG
GACCGTCTGGATGTTCACCGTAGCGCGCGTCACCACCTGAGCTTTGGTTAC
GGCGTGCACCAGTGCCTGGGTCAATATCTGGCGCGTCTGGAACTGGTGGTT
ATCCTGACCGTTCTGTTCGACCGTATCCCGACCCTGCGTCTGGCGGTGCCG
GTTGAACAGCTGACCCTGCGTCCGGGCACCACCATCCAAGGCGTGAACGA
ACTGCCGGTTACCTGGCACCACCACCACCACCACTAA

3. Gene sequence of CYP105D7

ATGGAAATCGGCAAGAGCAAAAGCAGCCCGGCGCGTAGCTTCATGACCGA
GCCGGGCACCAGCGTGAGCGCGCCGGTTGCGTTTCCGCAGGATCGTACCT
GCCCGTACGATCCGCCGACCGCGTATGACCCGCTGCGTGAAGGTCGTCCGC
TGAGCCGTGTTAGCCTGTACGACGGTCGTAGCGTGTGGGTGGTTACCGGTC
ATGCGGCGGCGCGTGCGCTGCTGAGCGATCAACGTCTGAGCAGCGACCGT
ACCCTGCCGCGTTTCCCGGCGACCACCGAGCGTTTTGAAGCGGTGCGTACC
CGTCGTGTTGCGCTGCTGGGCGTGGACGATCCGGAACACCGTACCCAACG
TCGTATGCTGGTTCCGAGCTTCACCCTGAAGCGTGCGGCGGCGCTGCGTCC
GCGTATCCAGGAGACCGTTGACGGTCTGCTGGATGCGATGGAAGCGCAGG
GTCCGCCAGCGGAGCTGGTTAGCGCGTTTGCGCTGCCGCTGCCGAGCATG
GTTATCTGCGCGCTGCTGGGTGTGCCGTATGCGGACCACGATTTCTTTGAA
AGCCAAAGCCGTCGTCTGCTGCGTGGTCCGGGCATTGCGGAAGTGCAGGA
TGCGCGTGCGCAACTGGACGATTACCTGTATGCGCTGATCGACCGTAAGCG
TAAAGAACCGGGTGACGGCCTGCTGGACGATCTGATTCAGGAACAACTGA
ACCGTGGCACCGTTGACCGTGCGGAGCTGGTGAGCCTGGCGACCCTGCTG
CTGATTGCGGGTCACGAGACCACCGCGAACATGATTAGCCTGGGCACCTTC
ACCCTGCTGCGTCACCCGGAACAACTGGCGGAACTGCGTGCGGAGCCGGG

TCTGATGCCAGCGGCGGTGGAGGAACTGCTGCGTTTTCTGAGCATCGCGGA
TGGCCTGCTGCGTGTTGCGACCGAGGACATCGAAGTGGCGGGCACCACCA
TTCGTGCGGATGAAGGCGTGGTTTTCGCGACCAGCGTTATTAACCGTGATG
CGGCGGGTTTTGCGGAACCGGATGCGCTGGATTGGCATCGTAGCGCGCGTC
ACCATGTGGCGTTCGGTTTTGGCATCCACCAGTGCCTGGGCCAAAACCTGG
CGCGTGCGGAGATGGAAATTGCGCTGGGCACCCTGTTCGAACGTCTGCCG
GGCCTGCGTCTGGCGGCTCCGGCGGACGAGATCCCGTTTAAACCGGGTGA
CACCATTCAAGGCATGCTGGAGCTGCCGGTGACCTGGCACCACCACCACC
ACCACTAA

4. Gene sequence of CamA

ATGGGCAACGCGAACGACAACGTGGTTATTGTTGGCACCGGTCTGGCGGG
TGTTGAGGTGGCGTTTGGTCTGCGTGCGAGCGGTTGGGAGGGTAACATTCG
TCTGGTTGGCGATGCGACCGTGATTCCGCACCACCTGCCGCCGCTGAGCAA
GGCGTACCTGGCGGGTAAAGCGACCGCGGAGAGCCTGTACCTGCGTACCC
CGGATGCGTATGCGGCGCAGAACATCCAACTGCTGGGTGGCACCCAGGTTA
CCGCGATTAACCGTGACCGTCAGCAAGTGATCCTGAGCGATGGCCGTGCGC
TGGACTATGATCGTCTGGTTCTGGCGACCGGTGGCCGTCCGCGTCCGCTGC
CGGTTGCGAGCGGCGCGGTGGGCAAGGCGAACAACTTTCGTTACCTGCGT
ACCCTGGAGGACGCGGAATGCATTCGTCGTCAACTGATCGCGGATAACCGT
CTGGTGGTTATTGGTGGCGGTTATATCGGTCTGGAAGTGGCGGCGACCGCG
ATTAAAGCGAACATGCATGTGACCCTGCTGGACACCGCGGCGCGTGTTCTG
GAACGTGTGACCGCTCCGCCGGTTAGCGCGTTCTATGAGCACCTGCACCGT
GAAGCGGGCGTTGACATCCGTACCGGCACCCAGGTGTGCGGTTTTGAGAT
GAGCACCGATCAGCAAAAGGTGACCGCGGTTCTGTGCGAAGATGGCACCC
GTCTGCCAGCGGACCTGGTTATCGCGGGTATTGGTCTGATTCCGAACTGCG
AGCTGGCGAGCGCGGCGGGCCTGCAAGTTGACAACGGTATCGTGATTAAC
GAACACATGCAAACCAGCGACCCGCTGATTATGGCGGTGGGTGATTGCGCG
CGTTTCCACAGCCAGCTGTACGACCGTTGGGTTCGTATCGAGAGCGTGCCG
AACGCGCTGGAACAAGCGCGTAAGATTGCGGCGATCCTGTGCGGTAAAGT
TCCGCGTGACGAGGCGGCGCCGTGGTTTTGGAGCGATCAGTACGAGATCG
GTCTGAAGATGGTTGGCCTGAGCGAAGGTTATGACCGTATCATTGTGCGTG
GCAGCCTGGCGCAGCCGGATTTCAGCGTGTTCTACCTGCAAGGTGACCGTG
TGCTGGCGGTTGATACCGTGAACCGTCCGGTGGAGTTCAACCAGAGCAAA
CAAATCATTACCGACCGTCTGCCGGTTGAACCGAACCTGCTGGGTGATGAG
AGCGTGCCGCTGAAGGAAATCATTGCTGCGGCGAAAGCGGAACTGAGCAG
CGCGTAA

5. Gene sequence of CamB

ATGAGCAAGGTGGTTTACGTGAGCCATGATGGCACCCGTCGTGAGCTGGAC
GTGGCGGATGGTGTTAGCCTGATGCAGGCGGCGGTGTCTAACGGTATCTAC
GACATTGTTGGTGATTGCGGTGGCAGCGCGAGCTGCGCGACCTGCCACGT
GTATGTTAACGAGGCGTTCACCGACAAGGTTCCGGCGGCGAACGAGCGTG
AAATCGGTATGCTGGAGTGCGTTACCGCGGAACTGAAACCGAACAGCCGT

CTGTGCTGCCAGATCATTATGACCCCGGAACTGGATGGCATTGTGGTTGAC
GTTCCGGATCGTCAATGGTAA

6. Gene sequence of ChuA

ATGTCACGTCCGCAATTTACCTCGTTGCGTTTGAGTTTGTTGGCTTTGGCTG
TTTCTGCCACCTTGCCAACGTTTGCTTTTGCTACTGAAACCATGACCGTTAC
GGCAACGGGGAATGCACGTAGTTCCTTCGAAGCGCCTATGATGGTCAGCGT
TATCGACACTTCCGCTCCTGAAAATCAAACTGCTACTTCAGCCACTGATTTG
CTGCGTCATGTTCCTGGAATTACTCTTGATGGTACCGGACGAACCAACGGT
CAGGATGTAAATATGCGTGGCTATGATCATCGCGGCGTGCTGGTTCTTGTCG
ATGGTGTTCGCCAGGGAACGGATACCGGACACCTGAATGGCACTTTTCTCG
ATCCGGCGCTGATCAAGCGTGTTGAGATTGTTCGCGGACCTTCAGCATTAC
TGTATGGCAGTGGCGCGCTGGGTGGAGTGATCTCCTACGATACGGTCGATG
CAAAAGATTTATTGCAGGAAGGACAAAGCAGTGGTTTTCGTGTCTTTGGTA
CTGGCGGCACGGGGGACCATAGCCTGGGATTAGGCGCGAGCGCGTTTGGG
CGAACTGAAAATCTGGATGGTATTGTGGCCTGGTCCAGTCGCGATCGGGGT
GATTTACGCCAGAGCAATGGTGAAACCGCGCCGAATGACGAGTCCATTAAT
AACATGCTGGCGAAAGGGACCTGGCAAATTGATTCAGCCCAGTCTCTGAG
CGGTTTAGTGCGTTACTACAACAACGACGCGCGTGAACCAAAAAATCCGC
AGACCGTTGAAGCTTCTGATAGCAGCAACCCGATGGTCGATCGTTCAACAA
TTCAACGCGATGCGCAGCTTTCTTATAAACTCGCCCCGCAGGGTAACGACT
GGTTAAATGCAGATGCAAAAATTTACTGGTCGGAAGTCCGTATTAATGCGC
AAAACACGGGGAGTTCAGGCGAGTATCGTGAACAGATAACAAAAGGAGC
AAGGCTGGAGAACCGTTCCACTCTATTTGCCGACAGTTTCGCTTCTCACTT
ACTGACATATGGCGGTGAGTATTATCGTCAGGAACAACATCCGGGTGGCGC
GACGACGGGCTTCCCGCAAGCAAAAATCGATTTTAGCTCTGGCTGGCTACA
AGATGAGATCACCTTACGCGATCTGCCGATTACCCTGCTTGGCGGAACCCG
CTATGACAGTTATCGCGGTAGCAGCGACGGCTACAAAGATGTTGATGCCGA
CAAATGGTCATCTCGTGCGGGGATGACTATCAACCCGACCAACTGGCTGAT
GTTATTTGGCTCATATGCTCAGGCATTCCGCGCCCCGACGATGGGCGAAATG
TATAACGATTCTAAACACTTCTCGATTGGTCGCTTCTATACCAACTATTGGGT
GCCAAACCCGAACTTACGTCCGGAAACTAACGAAACTCAGGAGTACGGTT
TTGGGCTGCGTTTTGATGACCTGATGTTGTCCAATGATGCTCTGGAATTTAA
AGCCAGCTACTTTGATACCAAAGCGAAAGATTATATCTCCACGACCGTCGAT
TTCGCGGCGGCGACAACTATGTCGTATAACGTCCCGAACGCCAAAATCTGG
GGCTGGGATGTGATGACGAAATATACCACTGATCTGTTTAGCCTTGATGTGG
CCTATAACCGTACCCGCGGCAAAGACACCGATACCGGGGAATATATCTCCA
GCATTAACCCGGATACCGTTACCAGTACCCTGAATATTCCGATCGCTCACAG
CGGCTTCTCTGTTGGTTGGGTCGGTACGTTTGCCGATCGCTCAACACATATC
AGCAGCAGCTACAGCAAACAACCTGGCTATGGTGTGAATGATTTCTACGTC
AGTTATCAAGGGCAGCAGGCGCTCAAAGGCATGACCACTACTCTGGTATTG
GGCAACGCCTTCGATAAAGAGTACTGGTCGCCGCAAGGCATCCCACAGGA
TGGTCGTAACGGAAAAATTTTCGTGAGTTATCAATGGTAA

7. Gene sequence of PhuR

ATGCCGCTCTCCCCGCCCTTCGCCCTGCGCCCCTGCCTGGCCCTGCTGTTG
CTCAGCCCTTCCCTGGCCCTGGCGGGGAACGCCGTCCCGCTGACCCCGAC
CACCATCACCGCCACCCGTACCGAGCAGGCAGTGGATTCGGTGCCAAGCA
CCGTCAGCGTGCAGACCCGCGAACAACTGGACCGGCAGAACGTCAACAA
CATCAAGGAACTGGTGCGCTACGAACCGGGAGTCTCGGTCGGCGGCGCCG
GCCAGCGTGCCGGGATCACCGGCTACAACATCCGCGGCATCGACGGGAAC
CGCATCCTTACGCAGATCGACGGGGTCGAACTGCCCAACGACTTCTTCAGC
GGCCCCTACGCGCAGACCCACCGCAACTACGTCGATCCGGACATCGTAAAG
CGCGTGGAGATCCTTCGCGGCCCGGCCTCGGCGCTGTACGGCAGCAACGC
CATCGGCGGCGCGGTGAGCTACTTCACCCTCGACCCGTCGGACATCATCAA
GGACGGCAAGGACGTCGGCGCCCGGCTGAAGGCCGGCTACGAGTCGGCC
AGCCACTCCTGGTTGACCTCGGCCACCGTCGGCGGCCGCGCCGACGACTT
CGACGGCCTGCTGCATTATGGCTACCGCCAGGGCCACGAGACCGAATCCAA
CGGCGGCCACGGCGGCACCGGGCTCTCGCGCAGCGAAGCCAACCCGGAA
GACGCCGACAGCTACAGCCTGCTCGGCAAGCTGGGCTGGAACTACGCCGA
GGGCAGCCGCTTCGGGCTGGTCTTCGAGAAGTACAAGAGCGACGTCGATA
CCGACCAGAAGAGCGCCTATGGCGGCCCGTACGACAAGGGCAAGCCGGCC
ATCCCGCCGAGCATGCTGCCGGGCGGCATGTACCAGTGGCGCAAGGGCAA
CGACGCCCTGACTCGCGAGCGCTACGGCCTGGAGCACCATTTCCTGCTCGA
CAGCCAGGTCGCCGATCGCATCCAGTGGAGCCTGAACTACCAGTTGGCGA
AGACCGACCAGGCGACCCGCGAGTTCTACTACCCGATCACCCGCAAGGTC
CTGCGCACCCGCGACACTACCTACAAGGAACGCCTGTGGGTCTTCGACAG
CCAGTTGGACAAGAGCTTCGCCATCGGCGAGACCGAGCACCTGCTGAGCT
ACGGGATCAATCTCAAGCACCAGAAGGTCACCGGCATGCGCAGCGGCACC
GGCACCAACCTGGACACCGGCGCGGACAGCCCGCGCGATGCCCTGGAACG
CAGCAGCGACTTTCCCGATCCGACGGTGAAGACCTACGCCCTGTTCGCCCA
GGACAGCATCAGCTGGAACGACTGGACCTTCACTCCCGGCCTGCGTTACG
ACTACACGCGCATGGAGCCGCACATCACCGACGAGTTCCTGCGCACCATGA
AGCAGAGCCAGAACACCGCGGTCGACGAGTCGGACAAGAAATGGCACCG
GGTTTCGCCCAAGTTCGGCGTGACCTACGACTTCGCCCAGCACTACACCTG
GTACGGCCAATACGCCCAGGGCTTCCGCACGCCCACCGCCAAGGCGCTGT
ACGGTCGATTCGAGAACCTGCAGGCGGGCTACCACATCGAGCCTAACCCC
AACCTCAAGCCGGAAAAGAGCCAGAGCTTCGAGACCGGGTTGCGCGGCA
AGTTCGACGAAGGCAGCTTCGGTGTAGCGGTGTTCTACAACAAATATCGCG
ACTTCATCGACGAAGACGCCCTGAATACCGATAGCACCGGCGGCAACGGC
CAGACCTTCCAGTCCAACAACATCGAGCGGGCGGTGATCAAGGGCGTCGA
GCTCAAGGGCCGCCTGGAGCTGGGCGCCTTCGGCGCGCCGCAGGGGCTCT
ACACCCAGGGCAGCGTGGCCTACGCCTACGGTCGCAACAAGGACAACGGC
GAGCCGATCAACAGCGTCAACCCACTCACCGGAGTGTTCGGCCTGGGCTA
CGACGAAGCAGACGGCAACTACGGCGGGCTGCTCAGCTGGACCCTGGTCA
AACGCAAGGATCGCGTCGACGACAGCACCTTCCACGCCCCGGATGGCACC
GCCAGCCAGTTCAAGACCCCGGGCTTCGGCGTCCTCGACCTCAGCGCCTA
CTACAGGCTGAGCAAGGACCTGACCCTCAACGCCGGTCTCTACAACCTGA

CCGACAAGAAATACTGGCTGTGGGATGACGTGCGCGGCTACGACAGCGTC
GGCGAGGCTTCGGCGCTGGCCCCGGCCAACATCGACCGACTGTCCCAGCC
AGGCCGCAATTTCGCGGTCAACCTGGTCTGGGACATCTAA

8. Gene sequence of ADB1

CGTAGCAATCGTCGTGATCATACCGTGAGCACCCGTCAGAGCAATATT

9. Gene sequence of ADB2

GTGAGCAGCCGTCGTAGCCATCGTCGTAGCAATCGCTGCAGCAAT

10. Gene sequence of ADB3

CAGAGCAGCCGTCGTAGCCATCGTCGTAGCCACCGTCAGACCCATCAG

11. Gene sequence of $HrtR_L$

ATGCCCAAATCTACATATTTTAGTCTATCAGACGAGAAACGTAAACGTGTTT
ACGATGCGTGCCTGCTGGAGTTTCAGACCCACTCGTTCCATGAGGCTAAAA
TCATGCACATCGTGAAAGCACTGGACATCCCGCGTGGTAGCTTCTATCAGT
ACTTTGAAGATCTTAAGGACAGCTATTACTACATTCTGTCCCAAGAAACGG
TTGAAATTCATGATCTGTTTTTCAACCTGTTGAAGGAGTACCCGCTGGAGG
TTGCCCTCAACAAGTACAAGTATCTGTTACTGGAAAATTTGGTTAATTCTCC
ACAGTATAACCTGTATAAGTACCGCTTTCTGGACTGGACTTATGAACTGGAA
CGTGATTGGAAACCGAAAGGTGAAGTAACCGTGCCGGCGAGAGAACTGG
ACAACCCGATTTCCCAAGTGCTGAAGAGCGTTATTAGTAATCTGGTCTATCG
CATGTTCAGCGAAAACTGGGATGAGCAAAAATTCATCGAGACGTACGACA
AGGAGATCAAACTGTTGACCGAGGGCTTGTTGAACTACGTGACCGAGAGC
AAAAAGTAA

12. Gene sequence of FhtR

ATGGAAAAACCCAATAAAAGGAAGGCTGATTTAGAACGTACTCGCCAGATC
ATCCTGTCTGTGGCCAGCGAATTGTTCATGACCAAAGGTTTTAAGAACACG
TCGACCAGAGAAATTGCGCTGAAGGCTAATATCACCCAGCCGAATTTGTAC
CACCACTTTAAAAACAAGAAAGAATTGTACCTGGCGGTTATTGAAGAACTT
ACCAGCCGTGTCCAGGAGGAGTTGGTTCCGATTGTCAGCGGTAATGCATCC
GTGGAAGAGAAGCTGTATCAACTGATTAAAGTTTTGCTGGATGAACATCCA
ACGAACCTGTTCCTGATGCTGAATGATATGTTTCAGGAGATGGGTCCGGATT
ATAACCGCACCCTGTATCAAATCTTCAAGAAGACCTATATCAACAACATTGC
GGCAATTTTCGAGAGCGAGCCGGAGACAAACTGCCTGCAGGAGGGCATCA
GCGTGGACGACACCACCCGTTTCATCCTGTACAACGTGTCCGCGCTGCTGT
CTATCGAGAAGACGTACCAACGTAAAACCGTTGATGACGACGTGAAAAAG
TTCATCCAATTTATGCTGCACGGCGTTCTCAAAAGCCATAAATAA

13. Gene sequence of hssSs

ATGTTTAAAACCCTGTACGCGCGTATCGCGATTTATAGCATCACCGTGATTCT
GTTTAGCGCGCTGATCAGCTTCGTGCTGACCAACGTTTACTATCACTACAAC

CTGAAGGCGAGCAACGACGCGAAGATCATGAAAACCCTGAAAGAGGCGC
GTCAGTATGAACAAAGCGCGAAACCGACCCACATTCAGCAATACTTCAAG
CACCTGGGTCAAATGAACTATCAGATCATGACCATTGACCAGAAAGGTCAC
AAGACCTTTTACGGCGAGCCGTTCCGTGAAGATACCCTGAGCCAAAACGC
GATCAACAACGTGCTGAACAACCAGGACTATCACGGTATTAAGGATAAACC
GTTCGCGCTGTTTGTTACCGGCTTCTTTGACAACGTGACCGATAACACCGT
TGGTATCAACTTTAAGACCAAAGACGGCAGCATTGCGGTGTTCATGCGTCC
GGATATCGGCGAGACCTTCAGCGAATTTCGTACCTTCCTGGCGGTTCTGCT
GATGCTGCTGCTGTTTATCAGCATTAGCCTGGTGATCGCGAGCACCTACAGC
ATCATTCGTCCGGTTAAGAAACTGAAACTGGCGACCGAGCGTCTGATTGAC
GGCGATTTCGAAACCCCGATCAAACAAACCCGTAAGGACGAGATTGGCAC
CCTGCAATATCACTTTAACAAGATGCGTGAAAGCCTGGGCCAGGTTGATCA
AATGCGTCAGCACTTCGTGCAGAACGTTAGCCACGAGATTAAAACCCCGCT
GACCCACATCCACCACCTGCTGAGCGAGCTGCAACAAACCAGCGACAAGA
CCCTGCGTCAGCAATACATCAACGATATTTATACCATCACCACCCAACTGAG
CGGTCTGACCACCGAGCTGCTGCTGCTGAGCGAACTGGACAACCACCAGC
ACCTGCTGTTTGACGATAAAATTCAAGTGAACCAGCTGATCAAGGATATCA
TTCGTCACGAGCAGTTTGCGGCGGACGAGAAGAGCCTGATCATTCTGGCG
GATCTGGAGAGCATCAACTTCCTGGGCAACCAACGTCTGCTGCACCAGGC
GCTGAGCAACCTGCTGATTAACGCGATCAAATACACCGACGTTGGTGGCGC
GATCGATATTGCGCTGCAACACAGCCACAACAACATCATTTTCACCATTAGC
AACGACGGTAGCCCGATCAGCCCGCAGGCGGAGGCGCGTCTGTTTGAACG
TTTCTACAAAGTGAGCAAGCACGATAACAGCAACGGTCTGGGCCTGGCGA
TCACCAAAAGCATCATTGAGCTGCACCACGGCACCATTCAATTTACCCAGA
GCAACGAATATGTTACCACCTTCACCATCACCCTGCCGAACAACAGCCTGT
AA

14. Gene sequence of hssR$_S$
ATGGTGCAGTGCCTGGTGGTTGACGATGACCCGCGTATCCTGAACTACATT
GCGAGCCACCTGCAAATCGAACACATTGATGCGTATACCCAGCCGAGCGGC
GAGGCGGCGCTGAAGCTGCTGGAAAAACAGCGTGTGGATATCGCGGTGGT
TGACATTATGATGGATGGTATGGACGGCTTCCAACTGTGCAACACCCTGAA
GAACGATTACGACATCCCGGTTATTATGCTGACCGCGCGTGATGCGCTGAG
CGACAAAGAGCGTGCGTTTATCAGCGGCACCGATGACTATGTGACCAAGCC
GTTCGAGGTTAAAGAACTGATCTTTCGTATTCGTGCGGTGCTGCGTCGTTAC
AACATCAACAGCAACAGCGAGATGACCATTGGCAACCTGACCCTGAACCA
GAGCTATCTGGAACTGCAAGTTAGCAACAAGACCATGACCCTGCCGAACA
AAGAGTTCCAACTGCTGTTTATGCTGGCGGCGCGTCCGAAGCAGATTTTCA
CCCGTGAGCAAATCATCGAAAAAATTTGGGGTTACGATTATGAGGGCGACG
AACGTACCGTGGACGTTCACATCAAGCGTCTGCGTCAGCGTCTGAAGAAA
CTGAACGCGACCCTGACCATTGAGACCGTTCGTGGTCAAGGCTACAAAGT
GGAAAACCACGTTTAA

15. Gene sequence of hssS$_B$

ATGAAAAGCCTGTATAGCCGTTTCGTGTTTATGACCGTTGGCATCATGCTGC
TGAGCAGCATCATTGGTTTCCTGCTGACCAACGTTTACTACCAAGTTAAGCT
GAAACCGTACAACAGCGAAAAGATTCTGAAATATGCGGAGGAAGTGAAAA
GCCTGTACGAAAAGCAGAGCGAGGAAAACCAGGAAGCGTACCTGCAAAG
CATCGCGAAGCTGGGCTACGAAATCTACATTGTTGACGATCAGAAGAACGG
CAAACGTATTGGTAACGCGTTCCGTAAAACCACCATCAGCGACGATACCAT
TCGTAAGGTGCTGAACGGCGAAACCTTCAACGGTGTTAGCACCTATCCGAC
CCGTCTGTTTATCACCGGTTTCTTTGACAACGAGCTGATTAACAGCGTGGG
CGTTCCGCTGAAACACGGTGACAAGCAATACGCGCTGTTTATCCGTCCGGA
TATTCAGAACCAATTCGGCGAAATGCGTATCTTTCTGGCGGTGCTGCTGGGT
TTCATTGTTCTGCTGAGCATCATTTTTATCGCGATTGCGGCGGGTTATATCGT
GCGTCCGATTCGTAAATTCACCAACGCGACCCAGAAGATCGCGAGCGGCG
AGTACGAAATTGAGCTGGACGTTAAGCGTAAAGATGAGATCGGCACCCTG
AGCACCAGCTTTCAGAAGATGACCAAAAGCATTAAGGAACTGGATGAGAT
GCGTCAAGAGTTCGTGAGCAACGTTAGCCACGAGTTTCAAAGCCCGCTGA
GCAGCATCCAGGGCTTCAGCAAAACCCTGCAAACCGAAAAGATGAGCGTG
GAGGAACGTAACCACTACCTGCAAATCATTGAAGGCGAGAGCAAACGTAT
GAGCAGCCTGTGCAAGCAACTGCTGACCCTGGCGAGCCTGGACAAAGAG
GAAAAGGTGCTGCAAATCAAAGAGTTCAACCTGCAAAAGCAAATCAAGGA
TGTTATCTTCATGCTGGAATGGAAATGGCGTGAGAAGAACATCGCGATTGA
ATTTGACGTGCCGGATATCACCATTAAAGGCGACGAGAACCTGCTGCACCA
AGTTTGGAGCAACATCTTCACCAACAGCATTAAGTTTAGCAACGAAGGTGG
CACCATCGAGTTCTTTGTGGAGGAACTGGAAAGCAGCGTTATCATTAGCAT
CAGCGACAACGGTATTGGCATGGAGAAAGAGGAAATGGACCGTATCTTCG
ATCGTTTTTATAAGGTGGATACCGCGCGTGCGCGTAACGTTGAAGGTAGCG
GCCTGGGTCTGAGCATCGTGCAGAAGATTGTTGAGCTGCACAACGGTAAC
GTGAGCGTTTACAGCACCAAAGGCGAAGGCACCACCGTGCGTGTTGAGCT
GCCGAAGTAA

16. Gene sequence of hssR$_B$

ATGAAGATGATCCACATTCTGCTGGCGGACGATGACAAACACATCCGTGAG
CTGCTGCACTACCACCTGCAAAAGGAAGGCTTCAAAGTTTTTGAGGCGGA
AGATGGCAAGGTTGCGCAAGAGGTGCTGGAGAAAGAAAACATCCACCTG
GCGATCGTTGATATTATGATGCCGTTTGTGGACGGCTACACCCTGTGCGAGG
AAATTCGTAAGTATCACGACATCCCGGTTATTCTGCTGACCGCGAAAGATCA
GCTGGTTGACAAGGAAAAAGGTTTCATCAGCGGCACCGATGACTACATTGT
TAAGCCGTTCGAGCCGGCGGAAGTGATCTTTCGTATGAAAGCGCTGCTGCG
TCGTTATCAAATGCTGAGCGCGGATATCATTACCCTGCACGGCACCACCATC
GACCGTAAGGGTGTTGAGGTGAAATGCAACGGTCAGACCATTCTGCTGCC
GCTGAAGGAGTTCGAACTGCTGAGCCAACTGGCGAGCTACCCGGGTCGTA
CCTTTAGCCGTGAGGAACTGATCGAACTGGTTTGGGGTATGGATTTCGAGG
GCGACGAACGTACCGTGGATGTTCACGTGAAGCGTCTGCGTGACCGTTTCA
GCAAACGTACCGATGACTTTCAGATTACCACCGTGCGTGGTCTGGGCTATA
AGCTGGAGCTGAAATAA

Supplementary References

[1] D. A. Hanna, R. M. Harvey, O. Martinez-Guzman, X. Yuan, B. Chandrasekharan, G. Raju, F. W. Outten, I. Hamza, A. R. Reddi, *Proc. Natl. Acad. Sci. U. S. A.* **2016**, *113*, 7539.

[2] D. A. Hanna, O. Martinez-Guzman, A. R. Reddi, *Biochemistry* **2017**, *56*, 1815.

[3] Z. Weissman, M. Pinsky, R. K. Donegan, A. R. Reddi, D. Kornitzer, *Cell. Microbiol.* **2021**, *23*, e13282.

[4] H. Zhou, B. Wang, F. Wang, X. Yu, L. Ma, A. Li, M. T. Reetz, *Angew. Chem. Int. Ed. Engl.* **2019**, *58*, 764.

[5] L. Ba, P. Li, H. Zhang, Y. Duan, Z. Lin, *Biotechnol. Bioeng.* **2013**, *110*, 2815.

[6] B. P. Pandey, C. Roh, K. Y. Choi, N. Lee, E. J. Kim, S. Ko, T. Kim, H. Yun, B. G. Kim, *Biotechnol. Bioeng.* **2010**, *105*, 697.

[7] K. Y. Choi, E. Jung, D. H. Jung, B. R. An, B. P. Pandey, H. Yun, C. Sung, H. Y. Park, B. G. Kim, *Microb. Cell Fact.* **2012**, *11*, 81.

[8] X. R. Zhao, K. R. Choi, S. Y. Lee, *Nat. Catal.* **2018**, *1*, 720.

[9] Q. Li, B. Sun, J. Chen, Y. Zhang, Y. Jiang, S. Yang, *Acta biochim. biophys. Sin.* **2021**, *53*, 620.

[10] Y. Jiang, B. Chen, C. Duan, B. Sun, J. Yang, S. Yang, *Appl. Environ. Microbiol.* **2015**, *81*, 2506.

[11] Y. Zhang, J. Yang, S. Yang, J. Zhang, J. Chen, R. Tao, Y. Jiang, J. Yang, S. Yang, *CRISPR J.* **2021**, *4*, 350.
